# An integrative framework to reevaluate the Neotropical catfish genus *Guyanancistrus* (Siluriformes: Loricariidae) with particular emphasis on the *Guyanancistrus brevispinis* complex

**DOI:** 10.1371/journal.pone.0189789

**Published:** 2018-01-03

**Authors:** Sonia Fisch-Muller, Jan H. A. Mol, Raphaël Covain

**Affiliations:** 1 Natural History Museum, Department of Herpetology and Ichthyology, Geneva, Switzerland; 2 Center for Agricultural Research in Suriname (CELOS) and Department of Biology, Anton de Kom University of Suriname, Paramaribo, Suriname; SOUTHWEST UNIVERSITY, CHINA

## Abstract

Characterizing and naming species becomes more and more challenging due to the increasing difficulty of accurately delineating specific bounderies. In this context, integrative taxonomy aims to delimit taxonomic units by leveraging the complementarity of multiple data sources (geography, morphology, genetics, etc.). However, while the theoretical framework of integrative taxonomy has been explicitly stated, methods for the simultaneous analysis of multiple data sets are poorly developed and in many cases different information sources are still explored successively. Multi-table methods developed in the field of community ecology provide such an intregrative framework. In particular, multiple co-inertia analysis is flexible enough to allow the integration of morphological, distributional, and genetic data in the same analysis. We have applied this powerfull approach to delimit species boundaries in a group of poorly differentiated catfishes belonging to the genus *Guyanancistrus* from the Guianas region of northeastern South America. Because the species *G*. *brevispinis* has been claimed to be a species complex consisting of five species, particular attention was paid to taxon. Separate analyses indicated the presence of eight distinct species of *Guyanancistrus*, including five new species and one new genus. However, none of the preliminary analyses revealed different lineages within *G*. *brevispinis*, and the multi-table analysis revealed three intraspecific lineages. After taxonomic clarifications and description of the new genus, species and subspecies, a reappraisal of the biogeography of *Guyanancistrus* members was performed. This analysis revealed three distinct dispersals from the Upper reaches of Amazonian tributaries toward coastal rivers of the Eastern Guianas Ecoregion. The central role played by the Maroni River, as gateway from the Amazon basin, was confirmed. The Maroni River was also found to be a center of speciation for *Guyanancistrus* (with three species and two subspecies), as well as a source of dispersal of *G*. *brevispinis* toward the other main basins of the Eastern Guianas.

## Introduction

Species identification, characterization, and naming remain fundamental and critical steps in biological science. Since the establishment of the Linnean system [1], species have been described mainly on the basis of morphological and phenotypic characteristics. Through time, however, morphology alone has been shown to be limited in its ability to delineate species boundaries, and led to a proliferation of names and nomenclatural instability [2]. In addition, cryptic diversity (reviewed in [3]) remained hidden from traditional morphological approaches (see e.g. [4, 5]). Modern technological developments, including DNA sequencing, provide new tools allowing the detection of hidden diversity. In particular, the DNA barcoding approach [6], quickly appeared to be an efficient methodology for detecting cryptic biodiversity (e.g. [7–15]), even though in certain cases, such as recent divergence [16, 17] or mitochondrial introgression [18], barcoding may fail to discriminate between species (e.g. [19, 20]). Morphological data (used in systematics) and molecular data such as DNA barcodes (used in biodiversity studies) are not mutually exlusive, and often are complementary means of delineating species. Indeed, combining multiple data sources is the most efficient way to support robust species hypotheses [21–23]. Formalized under the designation “*integrative taxonomy*” [2, 24] (reviewed in [25, 26]), this approach tries to use the complementarity of the different fields of study (e.g. morphology, genetics, biogeography, ecology, ethology, etc.) to delineate, describe and name species. Various protocols have been proposed to integrate these different data sources [2, 25–27], but most of them correspond to guidelines that explore the different datasets successively to corroborate taxonomic hypotheses, or only focus on a given type of data (e.g. [28]). The way that results of the different analyses are interpreted, i.e. in a cumulative or a congruent way [25], also has an impact on the results, leading to an over-estimation of the number of species and lower confidence in species identity in the former case, and to an underestimation of the number of species and higher confidendence in species identity in the latter. Moreover, comparing results of different analyses, which can be based on qualitatively different data (e.g. linear measurements for morphometric analyses, sequence alignments for phylogenetic trees or distances matrices, GPS coordinates for distributional data, etc.), in the same descriptive framework remains a challenge.

Community ecologists, confronted by the same issue of combined analysis of various data types, developed multi-table methods (e.g. [29–34]). Based on the co-inertia criterion [29], multi-table analyses look for common structures present in different data sets, and include them in a common analysis. The link between all tables is defined by row, since all different observations (e.g. the abundances, the distributions, the life traits, etc.) rely on the same statistical units (e.g. the specimens, the stations, etc.). These analyses are particularly flexible and allow the inclusion of multiple data types, and have already been used in different fields including e.g. ecology, medical research, agronomy, evolutionary biology, and genomics [19, 29, 31, 35–40]. In addition, these methods allow evaluation of the statistical significance of the congruence between data types and the amount of common information present in the different tables. We consider the integrative approach of multi-table methods highly appropriate for the resolution of species delineation in a group of poorly-differentiated catfishes from the Guianese Region.

The northeastern part of the Guiana Shield, including Suriname and French Guiana, overlaps the Eastern Guianas Neotropical freshwater ecoregion [41]. This region ranges between the Demerara River in the west and the Oyapock River in the east and probably supports more than 500 described species, of which 169 are considered endemic (freshwater ecoregions of the world: http://www.feow.org/ecoregions/details/311, accessed 31th Jan. 2017), making the Eastern Guianas a region of high biodiversity importance [42, 43]. The Eastern Guianas’ river system comprises about a dozen important catchments, including, from west to east, the Corantijn, Nickerie, Coppename, Saramacca, Suriname, Maroni (= Marowijne in Suriname), Mana, Sinnamary, Comté-Orapu, Approuague, and Oyapock rivers. All these catchments are independant and flow from south to north into the Atlantic, making the Eastern Guianas rather isolated from the rest of the Guiana Shield, out of the direct influence of the Amazon and Orinoco basins. Le Bail et al. [44] listed 416 species of freshwater and estuarine fishes in French Guiana and Mol et al. [45] 481 freshwater fish species in Suriname. Among this tremendous diversity, the Characiformes was the most important group, representing about 40% of all species, followed by the Silurifomes at around 35%. Among the latter, the Loricariidae is the most diversified catfish family with more than 80 species distributed in French Guiana and Suriname.

The Loricariidae is a strictly Neotropical catfish family comprising 937 valid species and an estimated 300 undescribed species distributed in more than 100 genera [46–48], making it the most species rich family of the Siluriformes. Loricariids are primarily characterized by a depressed body covered by bony plates, and by an important modification of the mouth into a sucker disk. Among Loricariidae, the subfamily Hypostominae represents half of the familial diversity, comprising 465 valid species [48] distributed in more than 40 valid genera [49]. In French Guiana and Suriname nine genera are recorded [44, 45], including hyperendemic and monotypic representatives such as *Hemiancistrus medians* or *Pseudoqolus koko* [20, 50, 51], both restricted to the Maroni Basin. The other genera are more widely distributed in South America, with the exception of *Guyanancistrus*, restricted to the northeastern part of the Guiana Shield.

Isbrücker [52] described the genus *Guyanancistrus*, designating *Lasiancistrus brevispinis* Heitmans, Nijssen & Isbrücker 1983, a species present in Suriname and French Guiana, as the type species. *Guyanancistrus* was originally diagnosed on the basis of its similarity to *Lasiancistrus* Regan 1904 while differing from the latter in the absence of the characteristic bristles, or whisker-like odontodes, that are found among the hypertrophied odontodes on their evertible cheek plates. *Guyanancistrus* was placed in the synonymy of *Pseudancistrus* Bleecker 1962 by Armbruster [53, 54], based on a phylogenetic analysis of morphological characters that included most of the genera then placed in the subfamilies Hypostominae and Ancistrinae. However, a molecular phylogenetic analysis of the group using mitochondrial and nuclear sequence data revealed *Pseudancistrus sensu lato* to be a paraphyletic assemblage of five unrelated lineages [50]. One of the lineages uncovered corresponded to the genus *Guyanancistrus*. As well as *G*. *brevispinis*, two other species were included in the genus: *G*. *niger* (Norman 1926) and *G*. *longispinis* (Heitmans, Nijssen & Isbrücker 1983), both described from French Guiana and restricted to the Oyapock River Basin [44]. Additionally, a possibly new dwarf species collected in mountain streams flowing to the Marowijne River in Suriname was placed as a member of *Guyanancistrus*, and constituted the sister species of *G*. *brevispinis* [50]. This small species (<6 cm) nicknamed Bigmouth due to its particular morphology [55] was already suspected to be new by Mol [56] who collected it during a Rapid Assessment Program (RAP) survey to the Nassau Mountains. This revealed a highly endemic fauna now threatened with extinction by a bauxite mining project and illegal gold mining [19, 45, 57].

Unlike its congeners, *Guyanancistrus brevispinis* is known to be widespread, common and abundant, its area of distribution covering all the main Guianese river systems of Suriname and French Guiana, from the Corantijn in the west to the Oyapock in the east [44, 55, 58, 59]. Cardoso and Montoya-Burgos [60] analysed the species based on several of its populations, including Amazonian ones (from northern tributaries of the Paru de Oeste and Jari rivers), in order to decipher its historical biogeography, and found that it was genetically highly diversified, with six distinct allopatric lineages (five Guianese and one Amazonian). It was thus considered as a species complex, with the true *G*. *brevispinis* possibly restricted to the Nickerie River system (see [55]). However, additional sources of information from genetic markers were deemed necessary to confirm their taxonomic status. The five Atlantic coastal *G*. *brevispinis* lineages of the Guianas were found to form a monophyletic group that originated from an ancestral colonization event from the Amazonian Basin, hypothesized to have been through river capture between northern Amazon tributaries and the upper Maroni River Basin. In the Guianas, subsequent dispersal would mainly have resulted from temporary connections between adjacent rivers when sea levels were low, and subsequent diversification of isolated populations during periods with high sea levels [60].

Considering the recent genus revalidation and questions about the type species, and the potential existence of new and/or endangered species, the present work uses an integrative approach combining morphology, genetics and spatial data to reappraise *Guyanancistrus*, focusing on the enigmatic *Guyanancistrus brevispinis* species complex. Most known populations were included in this analysis, principally based on material collected by the authors and their collaborators in the past 15 years. After a comparative diagnosis of the genus, the type species is redefined and its morphological and genetic variation delineated. Several new species revealed by the study are also described. Detailed descriptions and morphological comparisons of *Guyanancistrus niger* and *G*. *longispinis* are already available [59, 61, 62] and will not be repeated, but a practical key to all *Guyanancistrus* species is provided. After this taxonomic clarification, the biogeography of all *Guyanancistrus* members is re-evaluated to investigate dispersal processes, putative local extinctions, and speciation events.

## Materials and methods

### Ethics statement

No protected species (local restrictions, IUCN or CITES listed species) were examined in the study. Most specimens and tissue samples were obtained from museum collections and/or by local populations or fishermen. No experimentation was conducted on live specimens. For specimens and associated tissue samples obtained from the field, specimens were collected and exported with appropriate permits: Préfecture de la Région Guyane, Arrété 03/17/PN/EN to collect in the Réserve Naturelle des Nouragues in 2003; Ministry of Agriculture, Animal Husbandry and Fisheries to export fishes from Suriname in 2005, 2007, 2008, 2012, and 2014. Material obtained from the Parc Amazonien de Guyane in 2014 was collected under the direct supervision of PAG authorities. When collecting occurred in non protected areas of French Guiana, sampled specimens were declared to the French DEAL (French environmental protection ministry) before export. Immediately after collection, fish were anesthetized and sacrificed using water containing a lethal dose of eugenol (clove oil). Fin clips were taken after death and specimens fixed for long term preservation in museum collections. All work was conducted in accordance with relevant national and international guidelines, and conforms to the legal requirements (Directive 2010/63/EU of the European Parliament and of the Council on the protection of animals used for scientific purposes, the Swiss ordinance OPAn 455.1 of OSAV, and recommendations and regulations of DETA-DGNP permit number 20160422/01 AS).

### Materials

Materials examined for this study are deposited in the following institutions and collections: Auburn University Museum of Natural History (AUM); The Natural History Museum, London (BMNH); Museum of Comparative Zoology, Cambridge (MCZ); Muséum d’histoire naturelle, Genève (MHNG); Muséum national d’histoire naturelle, Paris (MNHN); Museu de Zoologia da Universidade de Sao Paulo (MZUSP); National Zoological Collection of Suriname (NZCS); National Museum of Natural History-Naturalis, Leiden (RMNH), presently holding the former Zoological Museum Amsterdam (ZMA) collection; Museum für Naturkunde, Berlin (ZMB); Zoologisches Staatssammlung, Munich (ZSM). Specimens included in morphometric analyses are indicated by an asterisk in specific lists of materials in main text and supplementary file (S1 Text), followed by number when needed.

### Morphology

Measurements and counts (S1 Table) were obtained from a total of 269 specimens, and were only carried out on one side in cases of paired characters. Specimens were measured with a digital calliper to the nearest 0.01 mm following Fisch-Muller et al. [5]. Measurements are presented in tabular form as percentages of standard length (SL) except for subunits of the head, which are expressed as percentages of head length (HL). Counts are the following: premaxillary and mandibular teeth were counted for the emergent row, adding obviously missing teeth shown by gaps in tooth rows. Dermal plate counts included: 1) lateral plates in the median series of rows, according to Schaefer [63], 2) plates bordering the supraoccipital, 3) predorsal plates, counted dorsally along a median line between supraoccipital and nuchal plate, 4) lateral plates of dorsal series along the dorsal-fin base, 5) lateral plates in dorsal series between end of dorsal-fin base and adipose-fin insertion, 6) lateral plates in dorsal series between adipose-fin insertion and caudal fin, 7) lateral plates in ventral series between the anal and the caudal fins, 8) lateral plates in dorsal series between end of dorsal fin when adpressed and adipose-fin spine insertion, and 9) lateral plates in dorsal series along unpaired median plate(s) preceeding adipose fin. All plate counts are whole numbers except (8) and (9) that were counted to the nearest half-plate. Dorsal-fin and anal-fin branched rays were counted; other fin-ray counts do not vary among Hypostominae species and were only obtained for type specimens and part of the non-type material.

### Morphometry

Morphometric and meristic data were subjected to multivariate analyses to reveal the morphological structure of the different species and populations of *Guyanancistrus* under study, with the addition of the possibly congeneric *Pseudancistrus megacephalus* to resolve taxonomic uncertainties. Prior to the analyses, all specimens smaller than 20 mm were excluded to minimize the bias introduced by allometric growth. Missing data, due to broken fin rays, were estimated for specimens belonging to a given population using the least squares method with the standard length (SL) used as the explanatory variable. Then, all morphometric data were standardized by SL and log transformed to control for size effect, to preserve and linearize allometric growth, and to prevent spurious correlations in the use of simple ratios [64]. Meristic data were used raw. The final table included data from 269 specimens belonging to 27 different morphs and populations, and contained 38 variables (24 morphometric and 14 meristic). This table was centered and reduced to allow comparison of variables expressed in different units, and submitted to a principal component analysis (PCA) using the correlation matrix to reveal its structuring. Because *Guyanancistrus* members appeared morphologically very close, and given the large number of variables in relation to the number of groups, a between group analysis (BGA) was secondarily performed on PCA results. To prevent artificial groupings, the different populations and morphs collected in different places for a given species were considered independently and used as a grouping factor (n = 27 groups). Prior to the BGA, a Monte Carlo permutation test on the value of between-group inertia was conducted using 9,999 random permutations to test against the absence of group effect. Multivariate analyses were performed using the ade4 1.7–4 [65] and ade4TkGUI 0.2–9 [66] packages in R 3.3.2 [67].

### Genetics

To estimate the genetic diversity and species boundaries of *Guyanancistrus* members, the 5’ region of the cytochrome *c* oxidase I (COI) mitochondrial gene was amplified for a DNA barcode analysis. In addition, a molecular phylogeny was reconstructed for 77 putative *Guyanancistrus* members and 12 outgroup species based on the analysis of mitochondrial and nuclear gene fragments (Table 1). Outgroup representatives were chosen from other genera, clades, and subfamilies of the Loricariidae following results of Covain & Fisch-Muller [50] and Lujan et al. [49]. The samples analyzed came from the tissue collection of MHNG. Markers were selected for their ability to resolve between- and within-species relationships, as well as deeper relationships at the intra-familial rank. For this we selected fast evolving markers such as the mtCOI, and the intronic regions of the nuclear *Fish Reticulon-4 receptor* (*f-rtn4r*) gene, whereas more conserved exonic regions of *f-rtn4r* and *recombination activating gene 1* (*rag1*) provided information for deeper relationships. Total genomic DNA was extracted with the DNeasy Tissue Kit (Qiagen) following the instructions of the manufacturer. The PCR amplifications were carried out using the Taq PCR Core Kit (Qiagen). To amplify and sequence in a single run the standard 650 bp barcode region with high quality, fragment size was increased to 900 bp using two newly designed primers: 5COI-F (5’-CTC GGC CAT CCT ACC TGT G-3’) and 5COI-R2 (5’-CGG GTG TCT ACG TCC ATT CCA ACT G-3’). The amplifications were performed in a total volume of 50 μl, containing 5 μl of 10x reaction buffer, 1 μl of dNTP mix at 10mM each, 1 μl of each primer at 10 μM, 0.2 μl of *Taq* DNA Polymerase equivalent to 1 unit of Polymerase per tube, and 1 μl of DNA. Cycles of amplification were programmed with the following profile: (1) 3 min. at 94°C (initial denaturing), (2) 35 sec. at 94°C, (3) 30 sec. at 53°C, (4) 55 sec. at 72°C, and (5) 5 min. at 72°C (final elongation). Steps 2 to 4 were repeated 39 times. Amplifications of the nuclear *rag1* and *f-rtn4r* genes followed Sullivan et al. [68] and Covain et al. [69] respectively. PCR products were purified with the High Pure PCR Product Purification Kit (Roche). Sequencing reactions were performed with the Big Dye Terminator Cycle Sequencing Ready Reaction 3.1 Kit (Applied Biosystems) following instructions of the manufacturer, and were loaded on an automatic sequencer 3100-Avant Genetic Analyzer (Applied Biosystems, Perkin-Elmer). Newly generated sequences were deposited in GenBank and BOLD with accession numbers provided in Table 1, while complementary sequences from previously published studies were obtained from GenBank (accession numbers and corresponding references in Table 1). The DNA sequences were edited and assembled using BioEdit 7.0.1 [70], aligned using ClustalW [71], and final alignment was optimized by eye.

**Table 1:** Taxa list, specimen and sequence data for *Cryptancistus similis* gen. nov. sp. nov., 76 *Guyanancistrus* members, and 12 outgroup representatives analyzed in this study. The acronyms of institutions are provided in the text.

Species	Catalog number	Type status	Field number	Locality	COI			*f-rtn4r*		*rag1*	
					BOLD	GenBank	Ref	GenBank	Ref	GenBank	Ref
*Cryptancistrus similis*	MZUSP 117150	H	SU07-672	Brazil, Paru de Oeste River	GBOL736-14	MG282930	This study	MG283003	This study	MG283064	This study
*Guyanancistrus brevispinis bifax*	MHNG 2725.099		GF00-103	French Guiana, Marouini River	GBOL089-13	MG282922	This study	JN855772	Covain & Fisch-Muller, 2012	MG283057	This study
*Guyanancistrus brevispinis bifax*	MHNG 2722.068		GF03-031	French Guiana, Sinnamary River	GBOL714-14	MG282939	This study	MG283012	This study	MG283070	This study
*Guyanancistrus brevispinis bifax*	MHNG 2734.090	P	GFSU12-141	French Guiana, Mana River	GBOL708-14	MG282945	This study	MG283016	This study	MG283076	This study
*Guyanancistrus brevispinis bifax*	MNHN 2017–0448	H	GFSU12-140	French Guiana, Mana River	GBOL707-14	MG282946	This study	MG283017	This study	MG283077	This study
*Guyanancistrus brevispinis bifax*	MHNG 2683.050		GF06-530	French Guiana, Mana River	GBOL705-14	MG282947	This study	FJ264188	Cardoso & Montoya-Burgos, 2009	MG283078	This study
*Guyanancistrus brevispinis bifax*	MHNG 2683.043		GF06-481	French Guiana, Maroni River	GBOL704-14	MG282948	This study	FJ264179	Cardoso & Montoya-Burgos, 2009	MG283079	This study
*Guyanancistrus brevispinis bifax*	MHNG 2683.043		GF06-480	French Guiana, Maroni River	GBOL703-14	MG282949	This study	FJ264178	Cardoso & Montoya-Burgos, 2009	MG283080	This study
*Guyanancistrus brevispinis bifax*	MHNG 2722.071		RV-19	French Guiana, Sinnamary River	GBOL723-14	MG282960	This study	NA	-	NA	-
*Guyanancistrus brevispinis bifax*	MHNG 2722.071		RV-13	French Guiana, Sinnamary River	GBOL722-14	MG282961	This study	NA	-	NA	-
*Guyanancistrus brevispinis bifax*	MHNG 2722.071		RV-12	French Guiana, Sinnamary River	GBOL721-14	MG282962	This study	MG283024	This study	NA	-
*Guyanancistrus brevispinis bifax*	MHNG 2722.071		RV-11	French Guiana, Sinnamary River	GBOL717-14	MG282963	This study	NA	-	NA	-
*Guyanancistrus brevispinis bifax*	MHNG 2699.060		GF07-107	French Guiana, Mana River	GBOL719-14	MG282968	This study	MG283027	This study	MG283095	This study
*Guyanancistrus brevispinis bifax*	MHNG 2683.029		GF06-467	French Guiana, Maroni River	GBOL718-14	MG282969	This study	MG283028	This study	MG283096	This study
*Guyanancistrus brevispinis bifax*	MHNG 2722.071		RV-11B	French Guiana, Sinnamary River	GBOL720-14	MG282971	This study	MG283030	This study	MG283098	This study
*Guyanancistrus brevispinis bifax*	MHNG 2683.050		GF06-531	French Guiana, Mana River	GBOL706-14	MG282975	This study	FJ264188	Cardoso & Montoya-Burgos, 2009	MG283101	This study
*Guyanancistrus brevispinis bifax*	MHNG 2757.027		GFSU14-043	French Guiana, Marouini River	GBOL1061-16	MG282985	This study	MG283042	This study	MG283109	This study
*Guyanancistrus brevispinis bifax*	MHNG 2757.027		GFSU14-044	French Guiana, Marouini River	GBOL1062-16	MG282986	This study	MG283043	This study	MG283110	This study
*Guyanancistrus brevispinis brevispinis*	MHNG 2621.073		SU01-121	Suriname, Nickerie River	GBOL090-13	MG282923	This study	JN855773	Covain & Fisch-Muller, 2012	MG283058	This study
*Guyanancistrus brevispinis brevispinis*	MHNG 2717.049		SU08-272	Suriname, Marowijne River	GBOL702-14	MG282950	This study	MG283018	This study	MG283081	This study
*Guyanancistrus brevispinis brevispinis*	MHNG 2717.049		SU08-271	Suriname, Marowijne River	GBOL701-14	MG282951	This study	MG283019	This study	MG283082	This study
*Guyanancistrus brevispinis brevispinis*	MHNG 2673.034		SU05-210	Suriname, Suriname River	GBOL699-14	MG282952	This study	FJ264197	Cardoso & Montoya-Burgos, 2009	MG283083	This study
*Guyanancistrus brevispinis brevispinis*	MHNG 2673.034		SU05-211	Suriname, Suriname River	GBOL700-14	MG282953	This study	FJ264196	Cardoso & Montoya-Burgos, 2009	MG283084	This study
*Guyanancistrus brevispinis brevispinis*	MHNG 2704.008		SU07-587	Suriname, Corantijne River	GBOL695-14	MG282956	This study	FJ264193	Cardoso & Montoya-Burgos, 2009	MG283087	This study
*Guyanancistrus brevispinis brevispinis*	MHNG 2704.008		SU07-586	Suriname, Corantijne River	GBOL694-14	MG282957	This study	FJ264192	Cardoso & Montoya-Burgos, 2009	MG283088	This study
*Guyanancistrus brevispinis brevispinis*	MHNG 2736.039		GFSU12-442	Suriname, Corantijne River	GBOL693-14	MG282958	This study	MG283022	This study	MG283089	This study
*Guyanancistrus brevispinis brevispinis*	MHNG 2736.039		GFSU12-441	Suriname, Corantijne River	GBOL692-14	MG282959	This study	MG283023	This study	MG283090	This study
*Guyanancistrus brevispinis brevispinis*	MHNG 2767.075		SU01-410	Suriname, Nickerie River	GBOL745-14	MG282964	This study	FJ264191	Cardoso & Montoya-Burgos, 2009	MG283091	This study
*Guyanancistrus brevispinis brevispinis*	MHNG 2767.075		SU01-409	Suriname, Nickerie River	GBOL744-14	MG282965	This study	FJ264190	Cardoso & Montoya-Burgos, 2009	MG283092	This study
*Guyanancistrus brevispinis brevispinis*	MHNG 2767.074		SU01-398	Suriname, Nickerie River	GBOL743-14	MG282966	This study	MG283025	This study	MG283093	This study
*Guyanancistrus brevispinis brevispinis*	MHNG 2767.074		SU01-397	Suriname, Nickerie River	GBOL742-14	MG282967	This study	MG283026	This study	MG283094	This study
*Guyanancistrus brevispinis brevispinis*	MHNG 2722.044		SU01-401	Suriname, Nickerie River	GBOL698-14	MG282976	This study	MG283034	This study	MG283102	This study
*Guyanancistrus brevispinis brevispinis*	MHNG 2753.091		GFSU14-999	Suriname, Nickerie River	GBOL1471-16	MG282987	This study	MG283044	This study	MG283111	This study
*Guyanancistrus brevispinis brevispinis*	MHNG 2753.091		GFSU14-1000	Suriname, Nickerie River	GBOL1472-16	MG282988	This study	MG283045	This study	MG283112	This study
*Guyanancistrus brevispinis brevispinis*	MHNG 2753.091		GFSU14-1001	Suriname, Nickerie River	GBOL1473-16	MG282989	This study	MG283046	This study	MG283113	This study
*Guyanancistrus brevispinis brevispinis*	MHNG 2753.091		GFSU14-1002	Suriname, Nickerie River	GBOL1474-16	MG282990	This study	MG283047	This study	MG283114	This study
*Guyanancistrus brevispinis brevispinis*	MHNG 2758.058		GFSU14-1626 (1)	Suriname, Saramacca River	GBOL1480-16	MG282995	This study	MG283052	This study	MG283119	This study
*Guyanancistrus brevispinis brevispinis*	MHNG 2758.058		GFSU14-1626 (2)	Suriname, Saramacca River	GBOL1481-16	MG282996	This study	MG283053	This study	MG283120	This study
*Guyanancistrus brevispinis brevispinis*	MHNG 2758.058		GFSU14-1626 (3)	Suriname, Saramacca River	GBOL1482-16	MG282997	This study	MG283054	This study	MG283121	This study
*Guyanancistrus brevispinis orientalis*	MHNG 2723.014		GF99-183	French Guiana, Oyapock River	GBOL740-14	MG282927	This study	MG283001	This study	MG283062	This study
*Guyanancistrus brevispinis orientalis*	MHNG 2723.014		GF99-184	French Guiana, Oyapock River	GBOL741-14	MG282928	This study	FJ264176	Cardoso & Montoya-Burgos, 2009	MG283063	This study
*Guyanancistrus brevispinis orientalis*	MHNG 2662.092		GF03-167	French Guiana, Approuague River	GBOL716-14	MG282937	This study	MG283010	This study	MG283068	This study
*Guyanancistrus brevispinis orientalis*	MHNG 2662.092		GF03-166	French Guiana, Approuague River	GBOL715-14	MG282938	This study	MG283011	This study	MG283069	This study
*Guyanancistrus brevispinis orientalis*	MNHN 2017–0450	H	GF06-183	French Guiana, Oyapock River	GBOL713-14	MG282940	This study	MG283013	This study	MG283071	This study
*Guyanancistrus brevispinis orientalis*	MHNG 2682.074		GF06-411	French Guiana, Comté River	GBOL711-14	MG282942	This study	MG283015	This study	MG283073	This study
*Guyanancistrus brevispinis orientalis*	MHNG 2682.047		GF06-304	French Guiana, Comté River	GBOL710-14	MG282943	This study	FJ264177	Cardoso & Montoya-Burgos, 2009	MG283074	This study
*Guyanancistrus brevispinis orientalis*	MHNG 2682.047		GF06-302	French Guiana, Comté River	GBOL709-14	MG282944	This study	FJ264178	Cardoso & Montoya-Burgos, 2009	MG283075	This study
*Guyanancistrus brevispinis orientalis*	MHNG 2621.099		SU01-156	French Guiana, Approuague River	GBOL696-14	MG282955	This study	MG283021	This study	MG283086	This study
*Guyanancistrus brevispinis orientalis*	MHNG 2757.035		GFSU14-1379	French Guiana, Approuague River	GBOL1475-16	MG282991	This study	MG283048	This study	MG283115	This study
*Guyanancistrus brevispinis orientalis*	MHNG 2757.035		GFSU14-1380	French Guiana, Approuague River	GBOL1476-16	MG282992	This study	MG283049	This study	MG283116	This study
*Guyanancistrus brevispinis orientalis*	MHNG 2757.035		GFSU14-1381	French Guiana, Approuague River	GBOL1477-16	MG282993	This study	MG283050	This study	MG283117	This study
*Guyanancistrus brevispinis orientalis*	MHNG 2757.035		GFSU14-1382	French Guiana, Approuague River	GBOL1478-16	MG282994	This study	MG283051	This study	MG283118	This study
*Guyanancistrus brownsbergensis*	MHNG 2724.009	P	SU01-286	Suriname, Saramacca River	GBOL697-14	MG282954	This study	MG283020	This study	MG283085	This study
*Guyanancistrus brownsbergensis*	MHNG 2724.008	P	SU01-285	Suriname, Saramacca River	GBOL689-14	MG282977	This study	MG283035	This study	MG283103	This study
*Guyanancistrus brownsbergensis*	MHNG 2723.037	P	SU01-280	Suriname, Saramacca River	GBOL690-14	MG282978	This study	MG283036	This study	MG283104	This study
*Guyanancistrus brownsbergensis*	NZCS F 7094	P	SU01-291	Suriname, Saramacca River	GBOL691-14	MG282979	This study	MG283037	This study	MG283105	This study
*Guyanancistrus brownsbergensis*	MHNG 2745.066	P	JM14-003	Suriname, Saramacca River	GBOL726-14	MG282980	This study	MG283038	This study	MG283106	This study
*Guyanancistrus brownsbergensis*	NZCS F 7093	P	JM14-002	Suriname, Saramacca River	GBOL725-14	MG282981	This study	MG283039	This study	MG283107	This study
*Guyanancistrus brownsbergensis*	MHNG 2745.065	H	JM14-001	Suriname, Saramacca River	GBOL724-14	MG282982	This study	MG283040	This study	MG283108	This study
*Guyanancistrus longispinis*	MHNG 2725.100		GF99-204	French Guiana, Oyapock River	GBOL091-13	MG282924	This study	JN855757	Covain & Fisch-Muller, 2012	MG283059	This study
*Guyanancistrus longispinis*	MHNG 2725.100		GF99-205	French Guiana, Oyapock River	GBOL729-14	MG282970	This study	MG283029	This study	MG283097	This study
*Guyanancistrus longispinis*	MHNG 2681.067		GF06-153	French Guiana, Oyapock River	GBOL728-14	MG282973	This study	MG283032	This study	MG283099	This study
*Guyanancistrus longispinis*	MHNG 2681.067		GF06-152	French Guiana, Oyapock River	GBOL727-14	MG282974	This study	MG283033	This study	MG283100	This study
*Guyanancistrus megastictus*	MNHN 2002–3508	H	Mit-01	French Guiana, Mitaraka Mountains	GBOL897-15	MG282983	This study	FJ264171	Cardoso & Montoya-Burgos, 2009	NA	-
*Guyanancistrus megastictus*	MHNG 2745.068	P	Mit-02	French Guiana, Mitaraka Mountains	GBOL898-15	MG282984	This study	MG283041	This study	NA	-
*Guyanancistrus nassauensis*	MHNG 2679.099	P	MUS 300	Suriname, Nassau Mountains	GBOL093-13	MG282926	This study	JN855774	Covain & Fisch-Muller, 2012	MG283061	This study
*Guyanancistrus nassauensis*	MHNG 2679.099	P	MUS 299	Suriname, Nassau Mountains	GBOL732-14	MG282936	This study	MG283009	This study	MG283067	This study
*Guyanancistrus niger*	MHNG 2722.089		GF99-185	French Guiana, Oyapock River	GBOL092-13	MG282925	This study	JN855759	Covain & Fisch-Muller, 2012	MG283060	This study
*Guyanancistrus niger*	MHNG 2682.037		GF06-295	French Guiana, Oyapock River	GBOL730-14	MG282941	This study	MG283014	This study	MG283072	This study
*Guyanancistrus niger*	MHNG 2727.049		RV-075	French Guiana, Oyapock River	GBOL731-14	MG282972	This study	MG283031	This study	NA	-
*Guyanancistrus niger*	MHNG 2753.072		GFSU14-1429	French Guiana, Oyapock River	GBOL1479-16	MG282998	This study	MG283055	This study	MG283122	This study
*Guyanancistrus tenuis*	MHNG 2745.067	P	Mit-05	Brazil, Mitaraka Mountains	GBOL739-14	MG282929	This study	MG283002	This study	NA	-
*Guyanancistrus tenuis*	MHNG 2745.067	P	Mit-04	Brazil, Mitaraka Mountains	GBOL738-14	MG282931	This study	MG283004	This study	NA	-
*Guyanancistrus tenuis*	MHNG 2745.067	P	Mit-03	Brazil, Mitaraka Mountains	GBOL737-14	MG282932	This study	MG283005	This study	NA	-
*Guyanancistrus teretirostris*	MZUSP 117149	H	SU07-654	Brazil, Paru de Oeste River	GBOL735-14	MG282933	This study	MG283006	This study	NA	-
*Guyanancistrus teretirostris*	MHNG 2723.004	P	SU07-653	Brazil, Paru de Oeste River	GBOL734-14	MG282934	This study	MG283007	This study	MG283065	This study
*Guyanancistrus teretirostris*	MHNG 2723.004	P	SU07-652	Brazil, Paru de Oeste River	GBOL733-14	MG282935	This study	MG283008	This study	MG283066	This study
Ancistrus macrophthalmus*	AUM 54994		T09397	Venezuela, Orinoco River	NA	NA	-	MG283056	This study	KP959934	Lujan *et al*., 2015
Corymbophanes kaiei*	ROM 89856		T12637	Guyana, Potaro River	NA	NA	-	NA	-	KP959937	Lujan *et al*., 2015
Dekeyseria scaphirhyncha*	AUM 43874		V5528	Venezuela, Orinoco River	NA	KP772574	Collins *et al*., 2015	JN855756	Covain & Fisch-Muller, 2012	KP959923	Lujan *et al*., 2015
Harttia guianensis*	MHNG 2662.091		GF03-160	French Guiana, Approuague River	GBOL003-12	JF292265	Covain *et al*., 2012	KR478219	Covain et al., 2016	NA	-
Hemiancistrus medians*	MHNG 2717.005		SU08-173	Suriname, Marowijne River	GBOL044-12	JF746999	Fisch-Muller *et al*., 2012	JF747012	Fisch-Muller *et al*., 2012	KP959995	Lujan *et al*., 2015
Hopliancistrus tricornis*	MHNG 2588.051		MUS 146	Brazil, Xingu River	NA	NA	-	JN855765	Covain & Fisch-Muller, 2012	KP960019	Lujan *et al*., 2015
Lasiancistrus schomburgkii*	MHNG 2651.068		GY04-308	Guyana, Essequibo River	GBOL129-13	MG283000	This study	JN855783	Covain & Fisch-Muller, 2012	MG283125	This study
Lithoxancistrus orinoco*	AUM 43725		V5246	Venezuela, Orinoco River	NA	NA	-	JN855766	Covain & Fisch-Muller, 2012	KP960027	Lujan *et al*., 2015
peckoltia sabaji*	MHNG 2651.016		GY04-029	Guyana, Essequibo River	GBOL051-12	JF747006	Fisch-Muller *et al*., 2012	JF747019	Fisch-Muller *et al*., 2012	KP959964	Lujan *et al*., 2015
Peckoltia simulata*	MHNG 2681.058	P	GF06-119	French Guiana, Oyapock River	GBOL048-12	JF747002	Fisch-Muller *et al*., 2012	JF747015	Fisch-Muller *et al*., 2012	NA	-
Pseudacanthicus leopardus*	MHNG 2651.024		GY04-025	Guyana, Essequibo River	GBOL052-12	JF746997	Fisch-Muller *et al*., 2012	JF747010	Fisch-Muller *et al*., 2012	KP960005	Lujan *et al*., 2015
Pseudancistrus barbatus*	MHNG 2653.059		GF00-074	French Guiana, Maroni River	GBOL130-13	MG282999	This study	JN855761	Covain & Fisch-Muller, 2012	MG283123	This study

* Outgroup

H = holotype

P = paratype

NA = data not available

- = absence of published reference

For the barcode analysis, the aligned COI sequences were converted into a distance matrix to evaluate sequence divergences using the Kimura 2 Parameter (K2P) metrics [72] with pairwise deletion for missing data as implemented in spider 1.3.0 [73] in R. This K2P matrix was used to compute between- and within-species distances to allow threshold optimization and evaluate the existence of a barcoding gap for correct species identification. A levelplot graph allowing a graphical representation of the distance matrix was also computed using the lattice 0.20–34 [74] and colorspace 1.2.7 [75] packages in R.

For the phylogenetic reconstruction, four partitions were created corresponding to the COI, *rag1*, exonic regions of *f-rtn4r*, and intronic regions of *f-rtn4r* genes. Two phylogenetic reconstruction methods allowing the analysis of partitioned data were used. First, a maximum likelihood (ML) reconstruction was performed with RAxML 7.2.6 [76] and raxmlGUI 1.0 [77] using the GTRGAMMA model [78, 79] with each partition assigned its own parameters. Robustness of the results was estimated by rapid bootstrapping [80, 81] with 1,000 pseudoreplicates. Second, a Bayesian inference analysis was conducted in MrBayes 3.2.6 [82, 83]. Two runs of four chains (one cold, three heated) were conducted simultaneously for 2 x 10^7^ generations using the same model as the ML analysis (nst = 6, rates = gamma, and each partition assigned its own parameters), with the tree space sampled each 1000th generation. After a visual representation of the evolution of the likelihood scores, and checking for the stationarity of all model parameters using Tracer 1.5 [84] (i.e. potential scale reduction factor (PSRF), uncorrected roughly approached 1 as runs converged [85], and Effective Sample Size (ESS) of all parameters above 200), the 2 x 10^6^ first generations were discarded as burn-in. The remaining trees were used to compute the consensus tree. Bayesian inference was performed using the CIPRES Science Gateway 3.3 [86].

### Distribution

To explore the distributional patterns of the different species of *Guyanancistrus*, georeferenced data were recorded for the localities of specimens deposited in official collections. These collections were selected for housing a large sampling of Guianese species, i.e. MHNG, MNHN, RMNH, and ZMA (the two latter now grouped in the Naturalis Biodiversity Center, Leiden). In addition, relative abundances were computed according to the number of specimens in each batch for each species and locality. Relative abundances per species and per locality were then ploted as pie charts onto a geographic map. Because of the heterogeneity of samples (i.e. comprising a collection of small and large numbers of specimens), a constant was added for each abundance estimate for readability (i.e. occurrence + abundance). The map was reconstructed using raster images and shapefiles obtained from the HydroSHEDS [87] project website (http://www.worldwildlife.org/pages/hydrosheds) in conjunction with the shapefiles 0.7 [88], mapplots 1.5 [89], raster 2.5.8 [90], and colorRamps 2.3 [91] packages in R. Pie charts were computed with the mapplots package.

### Multi-table analysis

Because *Guyanancistrus brevispinis* has been claimed to contain a species complex comprising at least five species [60], and that morphological characteristics were ambiguous, an integrative approach appeared necessary to clarify species boundaries. Because preliminary analyses provided information about the morphometric, phylogenetic, and spatial structures of *G*. *brevispinis*, the three types of information were united in a multiple co-inertia analysis (MCOA) [30] to identify the possible common structures of all datasets. For this, the three datasets were restricted to the subset of individuals and populations (n = 51 individuals distributed in 15 populations) for which all three types of information were available. Each of the three reduced tables was reanalysed separately. The morphometric data table was reanalysed by a PCA but with within-population variability eliminated by the computation of average values of the morphometric variables for each population. For the phylogenetic data table, a patristic distances matrix was computed from the branch lengths of the phylogenetic tree using ape 3.5 [92] package in R. Then, a principal coordinate analysis (PCoA) [93] using Cailliez [94] correction for non-Euclidian distance matrices was performed, to reveal its structuring. This analysis provided a tree-free representation of the distance matrix, where the pairwise distances between individuals on the axes are equal to the genetic pairwise distances of the matrix. The spatial structure was revealed by a PCoA performed on pairwise geographic distances computed from GPS coordinates using the geosphere 1.5.5 [90] package in R. A first assessment of a possible link between the three tables was obtained by performing pairwise Monte-Carlo permutation tests on the value of the RV coefficient [95] using 9,999 random permutations. Preliminary analyses, permutation tests, and multi-tables analyses were performed using the ade4 package in R.

### Biogeography

Finally, after clarification of all systematic issues, a reappraisal of the phylogeography of *Guyanancistrus* members was performed to elucidate the different routes of dispersal, and extinction and speciation events which occurred in the Eastern Guianas (*sensu* [41]). For this a Dispersal Extinction Cladogenesis (DEC) analysis [96, 97] was performed using the BioGeoBEARS 0.2.1 [98] package in R. The DEC model possesses two free parameters (*d* = dispersal and *e* = extinction) and allows maximum likelihood estimates of ancestral areas along branches and nodes of a phylogenetic tree. The DEC model implemented in BioGeoBEARS is equivalent to the one implemented in LAGRANGE [97] but with the possibility of adding an additional parameter *j* for founder effect events as an additional explanation for cladogenesis. For ancestral area reconstructions, the phylogenetic tree was reduced to the ingroup, polytomies resolved using ape in R, and 11 areas were defined that corresponded to the present catchment areas of the coastal rivers of the Guianas where *Guyanancistrus* members were collected, i.e. from west to east: the Corantijn, Nickerie, Saramacca, Suriname, Maroni, Mana, Sinnamary, Comté-Orapu, Approuague, and Oyapock rivers, with the addition of the Amazon Basin (including Paru de Oeste and Jari rivers) for species distributed outside of the Guianas. For the calculation, the maximum number of ancestral areas allowed to be reconstructed to a given node was set to four. The best model for the maximum likelihood reconstruction was evaluated by likelihood ratio test (LRT) since DEC and DEC + *j* were nested models differing in a single parameter (*j*).

### Nomenclatural acts

The electronic edition of this article conforms to the requirements of the amended International Code of Zoological Nomenclature, and hence the new names contained herein are available under that Code from the electronic edition of this article. This published work and the nomenclatural acts it contains have been registered in ZooBank, the online registration system for the ICZN. The ZooBank LSIDs (Life Science Identifiers) can be resolved and the associated information viewed through any standard web browser by appending the LSID to the prefix “http://zoobank.org/”. The LSID for this publication is: urn:lsid:zoobank.org:pub:72F36C36-69D7-4E59-AF6A-8A88C78CFD86. The electronic edition of this work was published in a journal with an ISSN, and has been archived and is available from the following digital repositories: PubMed Central, LOCKSS.

## Results

### Morphometry

**Fig 1:**
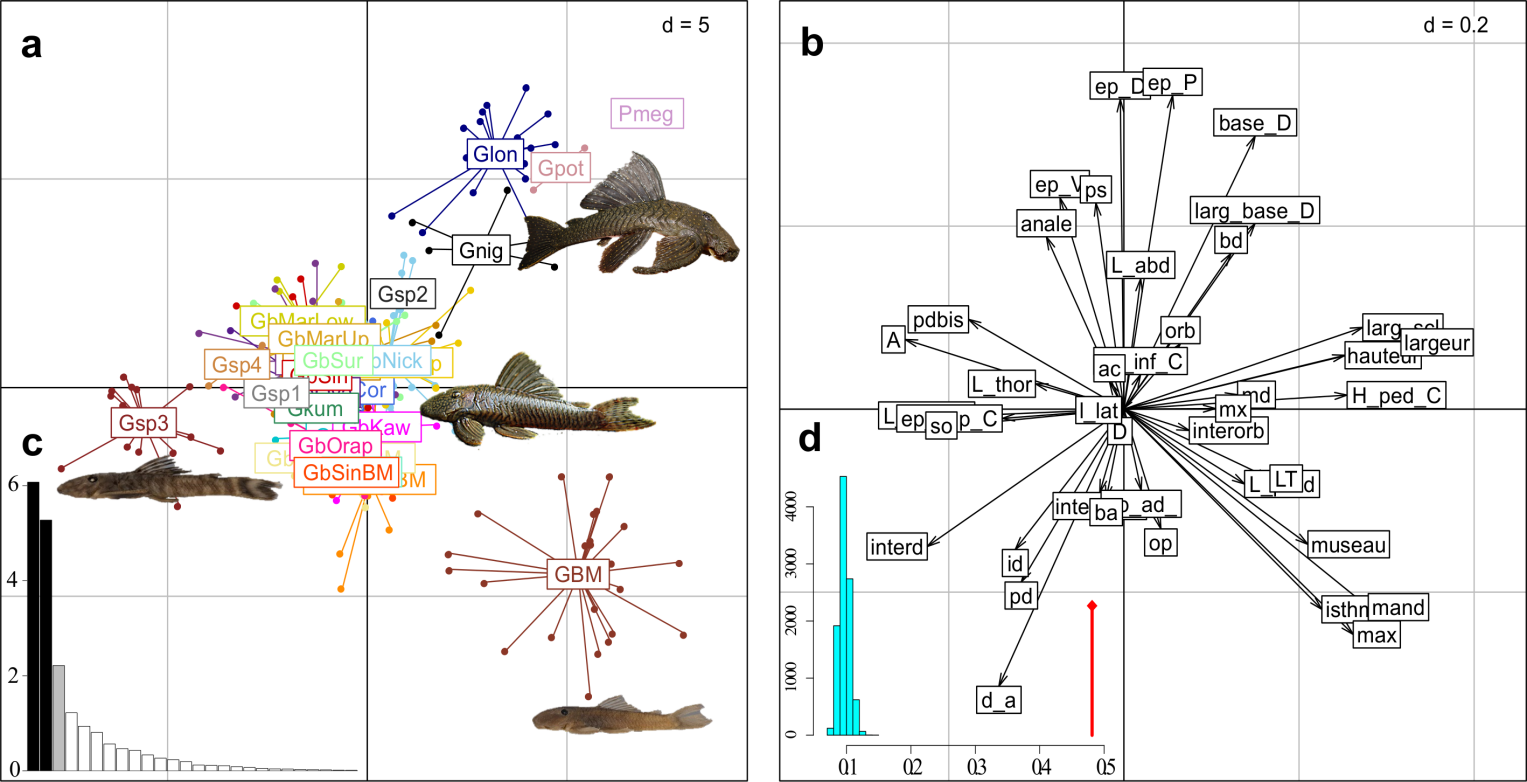
Between group analysis (BGA) of the different morphs and populations of putative *Guyanancistrus* members. **a**: projection of 269 specimens distributed in 27 groups onto the first factorial plane of the BGA (axis 1 horizontal, axis 2 vertical); GbKab: *G*. *brevispinis* Kabalebo River; GbCor: *G*. *brevispinis* Corantijn River; GbNick: *G*. *brevispinis* Nickerie River; GbSar: *G*. *brevispinis* Saramacca River; GbSur: *G*. *brevispinis* Suriname River; GbMarTap: *G*. *brevispinis*, Marowijne, Tapanahony River; GbMarUp: *G*. *brevispinis*, Upper Maroni River; GbMarLow: *G*. *brevispinis* Lower Maroni River; GbMarLowBM: *G*. *brevispinis* Lower Maroni River, big mouth morph; GbMana: *G*. *brevispinis* Mana River; GbManaBM: *G*. *brevispinis* Mana River, big mouth morph; Gbsin: *G*. *brevispinis* Sinnamary River; GbsinBM: *G*. *brevispinis* Sinnamary River, big mouth morph; GbOrap: *G*. *brevispinis* Orapu River; GbKaw: *G*. *brevispinis* Kaw River; GbApp: *G*. *brevispinis* Approuague River; GbOya: G. *brevispinis* Oyapock River; Gsp1: *G*. *teretirostris*; Gsp2: *Cryptancistrus similis* n. gen. n. sp.; Gsp3: *G*. *tenuis* n. sp.; Gsp4: *G*. *megastictus* n. sp.; GBM: *G*. *nassauensis* n. sp.; Gkum: *G*. *brownsbergensis* n. sp.; Glon: *G*. *longispinis*; Gnig: *G*. *niger*; Pmeg: `*Pseudancistrus*´ *megacephalus*; Gpot: *G*. sp. Potaro River. **b**: projection of the morphometric (n = 24) and meristic (n = 14) variables onto the first factorial plane of the BGA; variables labelled as in Tables 4 and 5. **c**: eigenvalues of the BGA. **d**: randomization test performed on the value of between-group inertia (9,999 replicates).

The between-group inertia recorded by the BGA represented 48.13% of the total inertia of the preliminary PCA (sum of eigenvalues of the BGA / sum of eigenvalues of the PCA: 19.73155 / 41 = 0.4812573). The permutation test (Fig 1D) was highly significant, with none of the null hypothesis sampling distribution of randomized values greater than the observed value of between-group inertia (X_obs_ = 0.4812573; p_Xrand_ ≥ p_Xobs_ = 0.0001). A significant group effect was thus present in the data, and observed between-group differences were not due to chance. Morphometric data were mainly structured on the first two axes of BGA (Fig 1C) which explained 57.52% of the total between-class inertia (30.78% for axis 1 and 26.74% for axis 2). The first morphometric plane (axes 1 and 2) of individuals split the different populations and species of *Guyanancistrus* into four main groups (Fig 1A). On the negative side of the first axis, the first group corresponded to a population originating from the northern slope of the Mitaraka Mountains (Gsp3; Jari River slope), followed by a mix of mostly *G*. *brevispinis* populations, thereafter called *brevispinis* group. In decreasing order of negative scores of variables (Fig 1B), species and population were rather characterized by higher values for number of branched anal-fin rays, interdorsal distance, number of plates bordering the supraoccipital, number of predorsal plates, caudal peduncle length, and upper caudal spine length. On the positive side, different species were aligned along the axis including *G*. *niger*, *G*. *longispinis*, the holotype of *Pseudancistrus megacephalus*, a population of *Guyanancistrus* from the Potaro River in Guyana also identified as *P*. *megacephalus* by Eigenmann in 1908 (Gpot), and a population from the Nassau Mountains (GBM). These species and populations were rather characterized by higher values of cleithral width, supracleithral width, caudal peduncle depth, and head depth at supraoccipital. Along the second axis, on the positive side, *G*. *niger*, *G*. *longispinis*, *P*. *megacephalus*, and the population from Potaro River were split from all other populations and species and constituted the *longispinis* group. Higher values of pectoral spine length, dorsal spine length, and dorsal-fin base length split the *longispinis* group from other *Guyanancistrus* members. On the negative side of Axis 2, the population from the Nassau Mountains was separated by higher values of premaxillary tooth cup length, interbranchial distance, and dentary tooth cup length. The *brevispinis* group remained poorly characterized morphologically, with all of its members grouped at the center of principal axes.

The morphometric approach was thus insufficient to delimit species boundaries of the *brevispinis* group members, and only six species could be characterized: *G*. *niger*, *G*. *longispinis*, *P*. *megacephalus*, a putatively three new species Gpot from Potaro River (= *P*. *megacephalus* sensu Eigenmann, 1908), Gsp3 from Mitaraka Mountains, and GBM from Nassau Mountains.

### DNA barcodes

**Fig 2:**
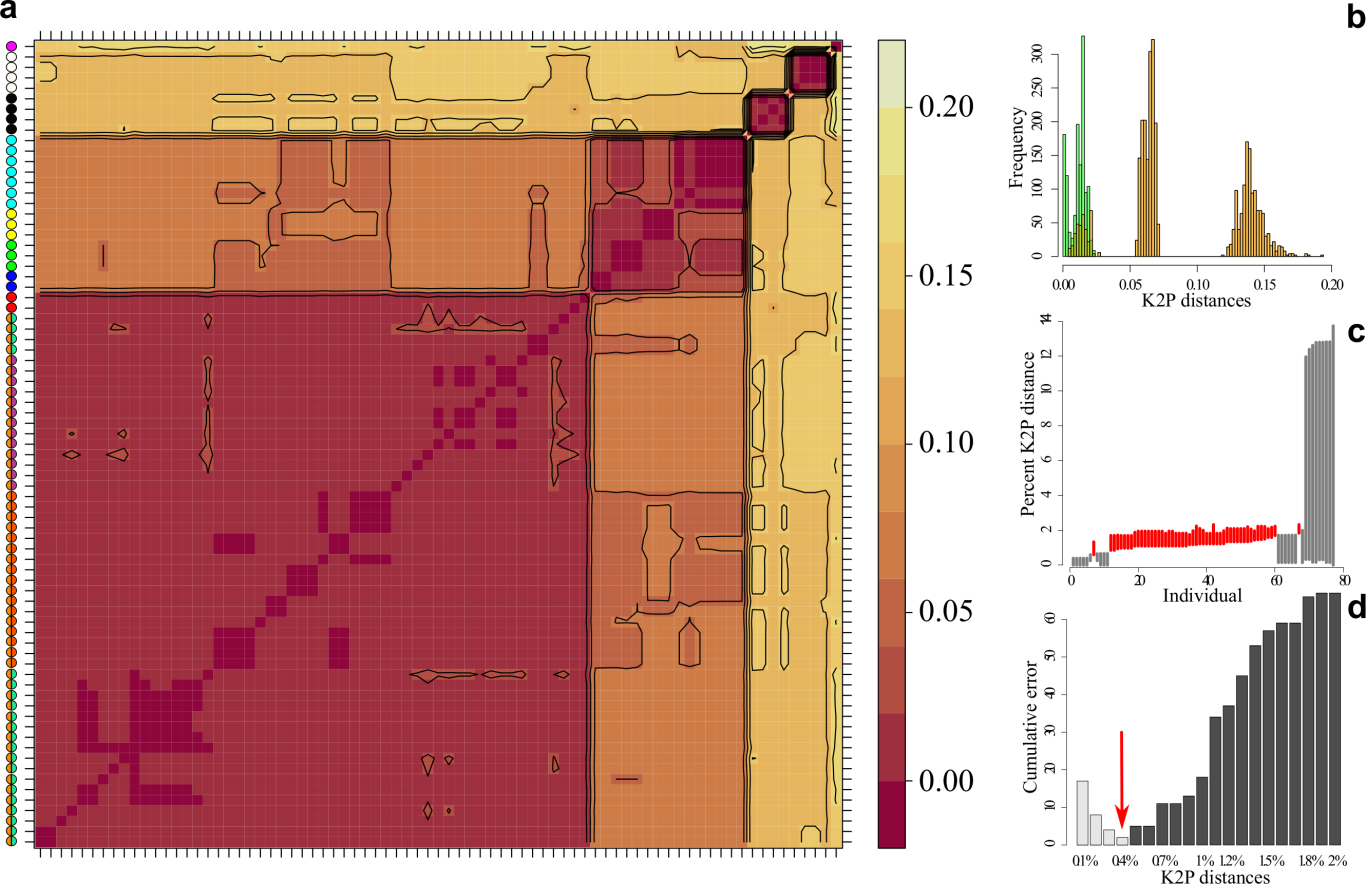
Analysis of the 77 DNA barcodes of *Guyanancistrus* spp. and *Cryptancistrus similis*. **a**: Levelplot of the K2P distances matrix computed on 889 bases of the mitochondrial COI gene; scale indicates the levels of variation in K2P distances; colored chips refer to species and subspecies following the color scheme of Fig 4**B**: Histogram of within (green) and between (orange) group variations in K2P distances (in abscise); scale (in ordinate) indicates the frequencies of pairwise comparisons in a definite range. **c**: Lineplot of the barcode gap for the 77 sequences of *Guyanancistrus spp*. and *C*. *similis*; for each individual, lines represent the difference between the furthest intraspecific distance (bottom of line value), and the closest interspecific distance (top of line value); positive differences (in grey) imply presence of barcoding gap whereas negative differences (in red) imply absence of barcoding gap. **d**: Barplot of threshold optimization; false positive rate of identification in light grey, and false negative in dark grey; red arrow indicates optimal threshold for the dataset.

The sequence alignment of 77 COI barcodes reached a total length of 889 positions. No insertions, deletions, or stop codons were observed in any sequence. Five lineages and three levels of variations were highlighted by the K2P distances heatmap (Fig 2A). The first lineage comprised all populations of *G*. *brevispinis* and the one from the Nassau Mountains. Within-group variations ranged between 0 and 0.023 (mean = 0.011) whereas between-group ones ranged between 0.174 and 0.003 (mean = 0.08). The second group was constituted by populations from the Mitaraka Mountains (Jari and Maroni sides), a population from Paru de Oeste River, and one from the Brownsberg Mountains. In this group, within-group distances ranged between 0 and 0.027 (mean = 0.012), and between-group distances between 0.055 and 0.156 (mean = 0.075). The third group was constituted by *G*. *niger* members (within-group K2P distances 0 < d < 0.003, mean = 0.002; between-group K2P distances 0.120 < d < 0.193, mean = 0.136), and the fourth one by *G*. *longispinis* members (within-group K2P distances 0 < d < 0.0016, mean = 0.0008; between-group K2P distances 0.120 < d < 0.193, mean = 0.136). The last group comprised a single representative from the Paru de Oeste River. This specimen displayed high between-group variations ranging between 0.13 and 0.17 (mean = 0.14) K2P distances. One level of variation was revealed in global within-group distances, whereas three levels were present in between-group distances (Fig 2B). Both global between- and within-group variations showed strong overlap with global within-group distances ranging between 0 and 0.023 (mean = 0.011), and global between-group distances ranging between 0.004 and 0.193 (mean = 0.088). The barcoding gap analysis revealed the absence of positive differences between the furthest intra-group distances and the closest non-specific for 51 individuals (Fig 2C). These individuals consisted mainly of representatives of *G*. *brevispinis*, the individuals from the Nassau Mountains, those from both sides of the Mitaraka Mountains, two specimens from Paru de Oeste and one from the Brownsberg Mountains, indicating that the barcoding approach was insufficient to discriminate all of the species of *Guyanancistrus* without ambiguity. The threshold optimization (Fig 2D) delivered a minimum of false positive matches and minimum cumulative error with a K2P distance of 0.004, much below the usually accepted threshold (around 1–2%).

The barcoding approach was thus unable to distinguish between intra and inter specific variations for several species, including members of the putative *G*. *brevispinis* complex, and only seven mitochondrial lineages could be identified (1: *G*. *brevispinis* including GBM from Nassau Mountains; 2: a mix of Gsp3 and Gsp4 from Mitaraka Mountains; 3: the weakly diferenciated Gsp1 from Paru de Oeste and 4: Gkum from Brownsberg Mountains; 5: *G*. *niger*, 6: *G*. *longispinis*, and 7: Gsp2 from Paru de Oeste).

### Molecular phylogeny

**Fig 3:**
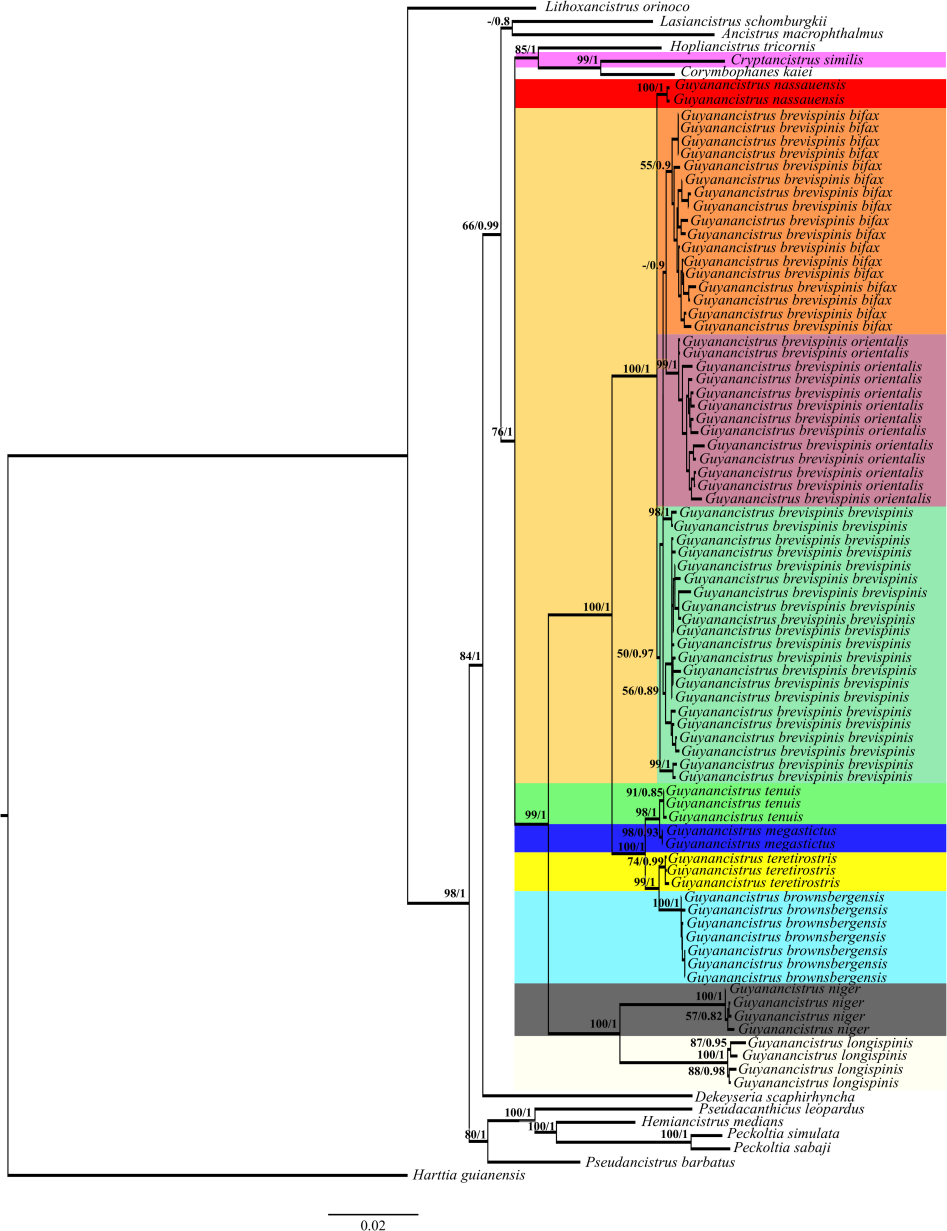
Maximum likelihood tree of the Ancistrini including *Guyanancistus* spp. and *Cryptancistrus* similis. Phylogenetic tree (ln L = -15182.6) inferred from the combined analysis of sequences of partial COI mitochondrial gene, and partial *f-rtn4r* and *rag1* nuclear genes. Numbers above branches correspond to bootstrap supports above 50 followed by posterior probabilities above 0.7. Background colors provide species boundries. When two colors are provided at the same level, left color refers to species limit whereas right color refers to subspecies limit; colors derived from Fig 4. Scale indicates the number of substitutions per site as expected by the model.

Given the poor results of the barcoding approach, nuclear marker sequences were added to the data set, and a molecular phylogeny reconstructed. In addition to the 77 mt COI gene fragments of *Guyanancistrus* members, 2 COI sequences of outgroup species, 56 sequences of the partial nuclear gene *f-rtn4r*, and 69 sequences of *rag1* were sequenced (Table 1). Forty three complementary sequences (6 of COI, 29 of *f-rtn4r*, and 8 of *rag1*) were obtained from GenBank using the accession numbers provided in Cardoso and Montoya-Burgos [60], Collins et al. [99], Covain and Fisch-Muller [50], Covain et al. [19], Covain et al. [69], Fisch-Muller et al. [20], and Lujan et al. [49]. Twenty one gene fragments did not amplify (4 of COI, 4 of *f-rtn4r*, and 13 of *rag1*) and were treated as missing data. The final sequence alignment included 3789 positions of which 889 corresponded to the mt COI gene, 1027 to the intronic and 864 to the exonic regions of *f-rtn4r*, and 1009 to the *rag1* gene. Maximum Likelihood and Bayesian phylogenetic reconstructions lead to identical tree topologies. The ML tree (Fig 3; Ln*L* = -15182.6) and Bayesian tree, both placed the specimen from the Paru de Oeste River, already distinct from all other *Guyanancistrus* in the barcoding approach, as a representative of a distinct genus, member of the outgroup, and sister genus of *Corymbophanes* with high statistical support [99 Bootstrap Probability (BP) and 1 Posterior Probability (PP)]. Both genera were nested in a clade comprising *Hopliancistrus tricornis* as sister group, and all three genera constituted the sister group of all *Guyanancistrus* members with high statistical support (76 BP, 1 PP). The *Guyanancistrus* clade was highly supported (99 BP, 1 PP) and split into two groups: one comprising *G*. *niger* with *G*. *longispinis* (100 BP, 1 PP), and a second comprising all other *Guyanancistrus* (100 BP, 1 PP). Within the latter, two new groups emerged: one comprising the population of the Nassau Mountains as the sister group of all *Guyanancistrus brevispinis* members (100 BP, 1PP) thus constituting a distinct species, and a second comprising all remaining species of *Guyanancistrus* (100 BP, 1 PP). Within the latter, two groups were highlighted, one comprising the population of the Jari side of the Mitaraka Mountains along with the one of the Maroni side (98 BP, 1 PP), and a second consisted in a population of the Paru de Oeste side of the Four Brothers Mountains, along with a population from the Brownsberg Mountains (99 BP, 1 PP). These four lineages constituted distinct species of *Guyanancistrus*. Within *G*. *brevispinis*, a first lineage originating from the Suriname River split from all other populations (50 BP, 0.97 PP). Then, with the exception of the polytomized population from the Tapanahony River (Marowijne Basin), three groups emerged: one comprising all populations from Corantijn, Nickerie, and Saramacca basins in Suriname (56 BP, 0.89 PP) sister to two sister groups, one constituted of all populations from Maroni (French Guiana), Mana, and Sinnamary rivers (55 BP, 0.90 PP), and the second comprising all populations of Comté-Orapu, Approuague, and Oyapock rivers in French Guiana (99 BP, 1 PP).

The phylogenetic analysis revealed the presence of seven species of *Guyanancistrus* and of a new genus and species in the data. No species complex was present within *G*. *brevispinis* but three lineages of infraspecific rank emerged.

### Distribution

**Fig 4:**
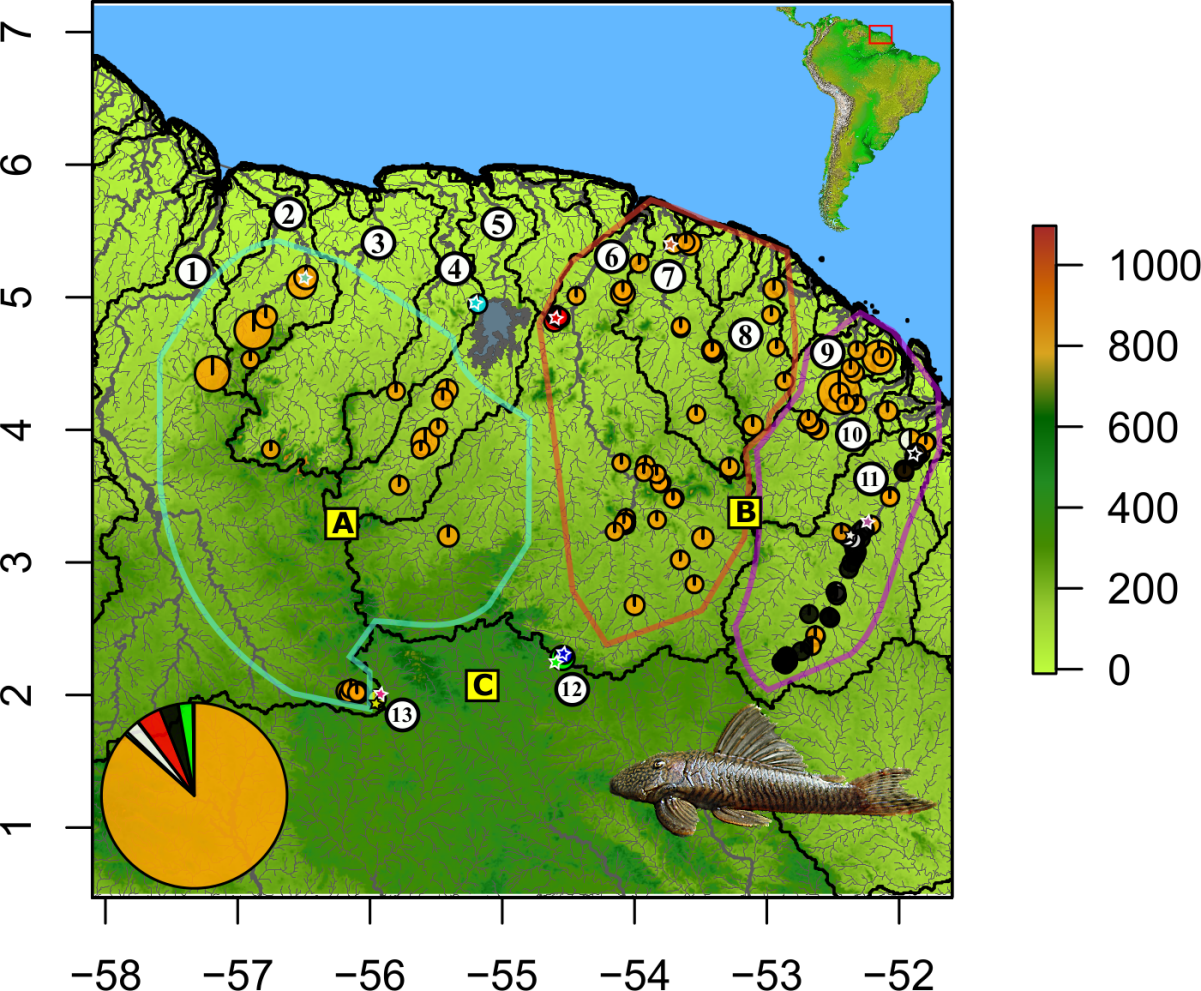
Geographic distribution and relative abundances of *Guyanancistrus* spp. and *Cryptancistrus similis*. Orange: *Guyanancistrus brevispinis*, red: *G*. *nassauensis*, light blue: *G*. *brownsbergensis*, yellow: *G*. *teretirostris*, green: *G*. *tenuis*, dark blue: *G*. *megastictus*, white: *G*. *longispinis*, black: *G*. *niger*, and pink: *Cryptancstrus similis*. Bold colored lines correspond to the areas of distribution of the three subspecies of *G*. *brevispinis*: water green: *G*. *b*. *brevispinis*; salmon: *G*. *b*. *bifax*; purple: G. *b*. *orientalis*. Pie charts represent the relative abundance of species per sampling locality (pie surface proportional to abundance). Stars refer to type localities. One star may overlap distinct localities. Pie legend represents the relative abundances of the different species for total sampling. Black lines represent limits of catchment areas; 1: Corantijn River, 2: Nickerie River; 3: Coppename River; 4: Saramacca River; 5: Suriname River; 6: Marowijne/Maroni River; 7: Mana River; 8: Sinnamary River; 9: Comté-Orapu River; 10: Approuague River; 11: Oyapock River; 12: Upper Jari River; 13: Upper Paru de Oeste River. Letters refer to countries; A: Suriname; B: French Guiana; C: Brazil. Horizontal axis: longitude in decimal degrees; left vertical axis: latitude in decimal degrees; right vertical axis: altitude in meters above mean sea level.

Different distribution patterns were present in the data (Fig 4). (1) Species could be widespread. This pattern characterized *G*. *brevispinis* which dominated the other species in terms of both occurrences and abundances. The species was distributed in all important drainages including the Corantijn, Nickerie, Saramacca, Suriname, Maroni, Mana, Comté-Orapu, Approuague, and Oyapock rivers, and represented 86.5% of all specimens collected. When *G*. *brevispinis* was co-distributed with other *Guyanancistrus* members, such as in the Oyapock Basin, it appeared less frequent and abundant. (2) Species could be restricted to a region including a few basins or a single basin. This pattern was observed for *G*. *niger* and *G*. *longispinis*, both endemic to the Oyapock River, but distributed throughout the Oyapock drainage. (3) Species could be hyperendemic and restricted to a single place. This pattern concerned species restricted to mountainous areas of the Nassau, Brownsberg, Four Brothers, and Mitaraka mountains. Three basins had different distribution patterns for different species; patterns 1 and 2 were present in the Oyapock, whereas patterns 1 and 3 were present in the Maroni and Saramacca rivers.

### Multi-tables analysis

**Fig 5:**
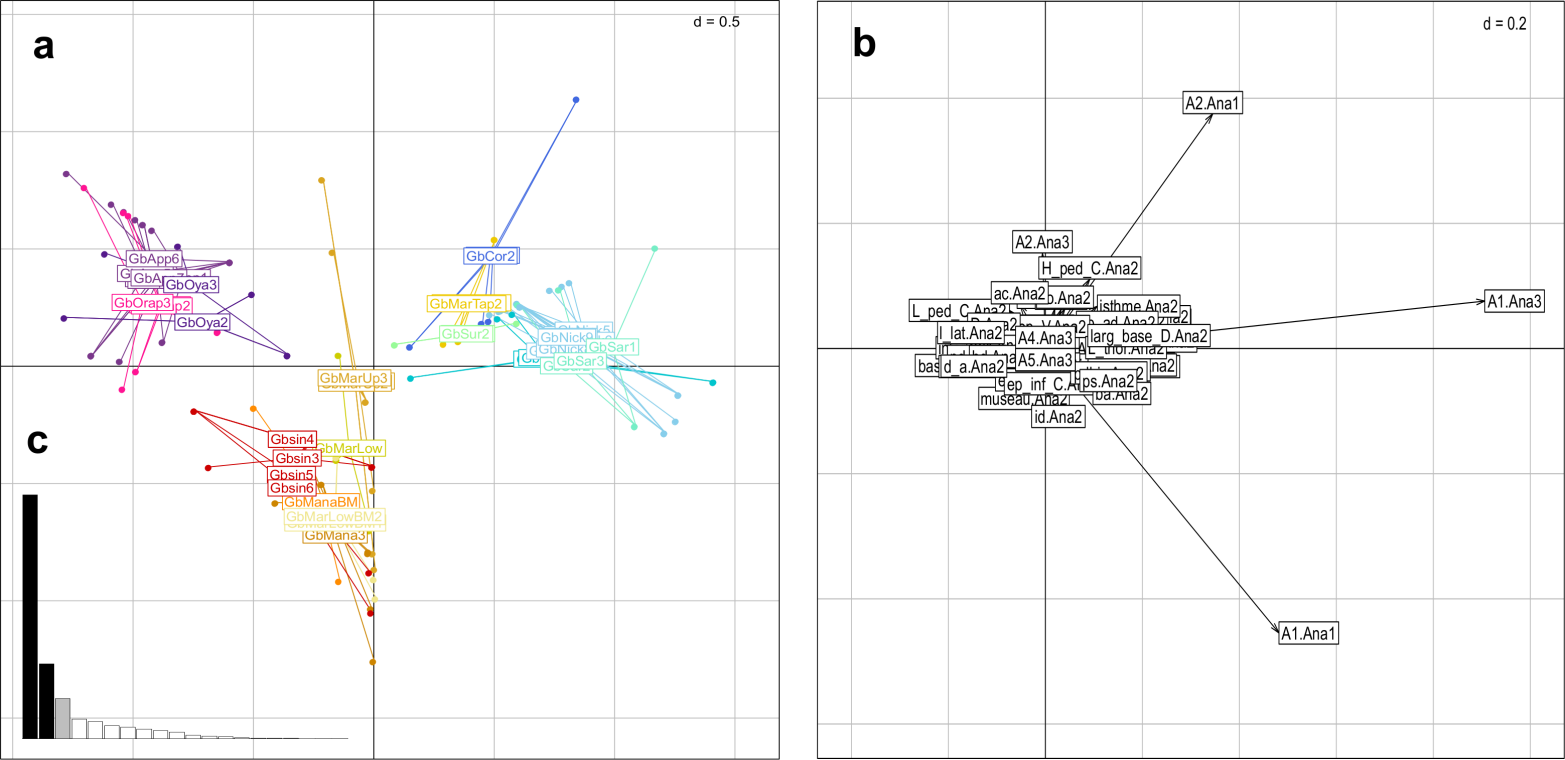
Multiple co-inertia analysis (MCOA) of *Guyanancistrus brevispinis* populations. Projection of data coordinates of preliminary analyses (PCoAs of geographic (Ana1) and phylogenetic (Ana3) data and PCA of morphometric (Ana2) data) onto axes 1 (horizontal) and 2 (vertical) of the MCOA. **a**: Compromise (labels) and superimposed normalized individuals’ scores of preliminary analyses (dots) in the multiple co-inertia plane; populations labelled as in Fig 1**B**: Coordinates of the variables in the first multiple co-inertia plane (labelled as in Table 4 for morphometric variables; PCos of geographic and phylogenetic data labelled in decreasing order, first PCos corresponding to greater distances). **c**: Eigenvalues of the MCOA.

Because only phylogenetic information was able to discriminate *G*. *brevispinis* among all other *Guyanancistrus* members (Table 2) and none of the five lineages claimed as putative new species [60] could be clearly delineated by the different analyses, the multi-table approach was applied. Prior to the analysis, the DNA barcode table was removed to minimize redundancy and avoid over weighting this information since the COI gene had been used to reconstruct the phylogeny. A first assessment of the relationships between genetics (i.e. the phylogeny), morphology, and geography was performed using pairwise RV tests between preliminary analyses of the reduced datasets (PCoA for genetics and geography, and PCA for morphometric data). All pairwise tests (Table 3) showed strong and significant vector correlations between tables (p-value = 0.0001), with the pairwise correlations indicating that the genetic data were slightly more correlated to the geography (RV = 0.566, p-value = 0.0001) and morphological data (RV = 0.532, p-value = 0.0001) than the latter were to the geography (RV = 0.491, p-value = 0.0001). However, all RV coefficients were globally comparable among pairwise comparisons with around 50% of common signal between tables. The first plane of MCOA accounted for 69.31% of the total co-structure (52.98% for axis 1 and 16.33% for axis 2) (Fig 5C). The amount of variation explained by MCOA axes was similar to those obtained in the separate analyses, but with a lesser contribution from morphology. Indeed, 99.86% ((0.396 + 0.338)/(0.457 + 0.278) = 0.734/0.735) of the genetic data structure, 69.2% of the morphological data structure, and 100% of the geographic data structure were represented on the first two axes of the MCOA (Co-Inertia/Inertia in Table 3). The contribution of each table to the quantity maximized by MCOA (i.e. sum of squared covariance between the linear combinations of the variables of each table and the compromise = Cov^2^ in Table 3) highlighted the relative importance of geography for the first axis, and of genetics for the second. Morphology contributed least to the compromise for both axes. The associated correlations (Cos^2^ in Table 3) showed that the first two axes of the compromise were strongly linked to each separated table except for the second axis derived from geographic data (0.890 and 0.962 for the genetic data, 0.865 and 0.758 for the morphometric data, and 0.965 and 0.157 for the geographic data). The first axis of the individuals’ plane of the MCOA (Fig 5A) aligned three groups of *G*. *brevispinis*. In negative scores, a first group comprised populations distributed in the Oyapock, Approuague and Comté-Orapu rivers in eastern French Guiana followed by a second group comprising populations in the Sinnamary, Mana and Maroni basins in central and western French Guiana but excluding those from the Tapanahony River, a western tributary of Maroni River in Suriname. In positive scores, a third group comprised populations from the Corantijn, Nickerie, Saramacca, Suriname, and Tapanahony rivers in Suriname. The second axis split the representatives of *G*. *brevispinis* from central and western French Guiana in negative scores from those from Suriname and eastern French Guiana. Correlations of variables of the preliminary analyses with MCOA axes (Fig 5B) showed high scores, in decreasing order of positive scores on axis 1, for the first principal coordinate of the PCoA of the geographic data table (i.e. the longitude), first and second principal coordinates of the PCoA of the phylogenetic data table (i.e. deeper structures of the phylogenetic tree restricted to *G*. *brevispinis* corresponding to the splitting of the different populations from Suriname and French Guiana, see Fig 3), opercle length, interorbital width, cleithral width, supracleithral width and interbranchial distance. For negative scores (in decreasing values of absolute values of negative scores) these variables corresponded to the: number of lateral plates, number of predorsal plates, dorsal–fin base length, number of plates between adpressed dorsal fin and adipose fin, caudal peduncle length and interdorsal distance. On the second axis the variables with greater scores corresponded, in decreasing order of positive scores, to the second principal coordinate of the PCoA of the phylogenetic table, second principal coordinate of the PCoA of the geographic table (i.e. the latitude), and caudal peduncle depth. In negative values, these variables corresponded, in decreasing order of absolute values, to the: first principal coordinate of the PCoA of the phylogenetic table, number of plates between dorsal-fin base and adipose-fin spine, number of plates along adipose-fin base (median platelets), snout length, and number of anal to caudal plates.

**Table 2:** Ability of the different methods to delineate without ambiguity species limits in *Guyanancistrus* spp. At least two congruent sources of information are expected to support taxonomic decisions.

	*G*. *niger*	*G*. *longispinis*	*G*. *nassauensis*	*G*. *brownsbergensis*	*G*. *teretirostris*	*G*. *tenuis*	*G*. *megastictus*	*G*. *brevispinis*
Morphometry	**✓**	**✓**	**✓**	**✘**	**✘**	**✓**	**✘**	**✘**
DNA barcodes	**✓**	**✓**	**✘**	**✓**	**✓**	**✘**	**✘**	**✘**
Phylogeny	**✓**	**✓**	**✓**	**✓**	**✓**	**✓**	**✓**	**✓**
Distribution	**✘**	**✘**	**✘**	**✓**	**✓**	**✓**	**✓**	**✘**
∑	**✓**	**✓**	**✓**	**✓**	**✓**	**✓**	**✓**	**✘**

✘ = negative result

**✓** = positive result

∑ = sum of congruent information allowing taxonomic decision

**Table 3:** Main characteristics of the multi-table analysis computed on the restricted data set (n = 51). Phylogeny: phylogenetic data table; Morphology: morphometric data table; Geography: distribution data table. RV test: pairwise tests of congruence among preliminary analyses. Results reported as RV coefficient of correlation in upper diagonal, and as p-values for α = 0.05 in lower diagonal. NA: comparison of the data table to itself not performed. MCOA: multiple co-inertia analysis. Inertia: maximum inertia projected onto the first two axes of the simple analyses (eigenvalues of the PCoA for the phylogenetic and distributional data, and eigenvalues of PCA for the morphometric data tables). Co-inertia: inertia of the three tables projected onto the first two multiple co-inertia axes. Cos2: correlation between the scores of each table and the synthetic variable of same rank (axes 1 and 2). Cov2: squared covariance between the scores of each table and the synthetic variable of same rank (maximized by the analysis); note that Cov2 provides the contribution of each table to the compromise established by the multiple co-inertia analysis.

	Phylogeny	Morphology	Geography
**RV test**			
Phylogeny	NA	0.532	0.566
Morphology	1.00E-04	NA	0.491
Geography	1.00E-04	1.00E-04	NA
			
**MCOA**			
**Inertia**			
Axis 1	0.457	0.282	0.851
Axis 2	0.278	0.231	0.149
**Co-Inertia**			
Axis 1	0.396	0.250	0.851
Axis 2	0.338	0.105	0.149
**Cos2**			
Axis 1	0.890	0.865	0.965
Axis 2	0.962	0.758	0.157
**Cov2**			
Axis 1	0.352	0.217	0.821
Axis 2	0.325	0.079	0.024

Three groups of infra-specific rank showing significantly structured data concerning their distribution, genetics, and morphology were consequently recognized.

### Taxonomic account and descriptions

#### *Guyanancistrus* Isbrücker in Isbrücker et al., 2001

*Guyanancistrus* Isbrücker in Isbrücker et al., 2001 [52]: 19 (type species: *Lasiancistrus brevispinis* Heitmans, Nijssen & Isbrücker, 1983 [62]; type by original designation; masculine); Fisch-Muller, 2003 [100]: 384 (checklist, valid); Armbruster, 2004 [54] (synonymization with *Pseudancistrus* based on morphological phylogeny); Ferraris, 2007 [47]: 287 (checklist, synonym of *Pseudancistrus*); Covain & Fisch-Muller, 2012 [50]: 235 (generic revalidation based on molecular phylogeny); Silva et al., 2014 [101]: 12 (validity confirmed based on same data); Lujan et al., 2015 [49]: 276 (phylogenetic placement in redefined tribe Ancistrini).

**Diagnosis.** *Guyanancistrus* was shown to be monophyletic based on mitochondrial and nuclear DNA sequences. No unique morphological character was found to diagnose the genus which belongs to the Ancistrini tribe of the Hypostominae subfamily. The following combination of characters distinguishes *Guyanancistrus* from all other Hypostominae genera: head and body dorsoventrally depressed; head and body plates not forming prominent ridge or crest; snout rounded and flattened; snout covered with plates except tip region and, sometimes, a small area on each side of tip of snout; plates on all parts of snout forming a rigid armor covered with numerous short odontodes; presence of odontodes over a broad area on the opercle; presence of enlarged cheek odontodes supported by evertible plates; these odontodes straight with tips slightly curved, not strongly hook-shaped; absence of whisker-like cheek odontodes; absence of enlarged odontodes along snout margin; presence of a dorsal iris operculum; lips forming an oval disk; dentary and premaxillary with numerous viliform and bicuspid teeth; presence of a small buccal papilla, no enlarged dentary papilla; seven branched dorsal-fin rays; presence of an adipose fin; no membranous extension between end of dorsal fin and adipose fin; five series of lateral plates extending to caudal fin; lateral plates not keeled and not bearing enlarged odontodes; lateral plates of ventral series on caudal peduncle angular but not keeled; abdominal region entirely naked. *Guyanancistrus* is mostly similar to *Cryptancistrus* n. gen in external appearance. It is distinguished from *Cryptancistrus* primarily by the uniformity of its snout plates and odontodes (in *Cryptancistrus*. posterior part of lateral margin of snout do not form a rigid armor but rather a soft fleshy border, and bears slightly enlarged odontodes with small tentacules sensu Sabaj et al. [102]). It can additionally be distinguished from *Cryptancistrus* by the presence of a skin region bordering the exposed portion of opercle that is distinctly narrower than the latter (*vs* roughly as large as the latter).

**Etymology.** The name *Guyanancistrus* was originally explained as a contraction of “Guyana” and the generic name *Ancistrus* Kner, 1854. Gender: masculine.

**Distribution.** Endemic to the Atlantic coastal rivers and upper Amazonian tributaries of the north-eastern Guiana Shield.

#### *Guyanancistrus brevispinis* (Heitmans, Nijssen & Isbrücker, 1983)

**Fig 6:**
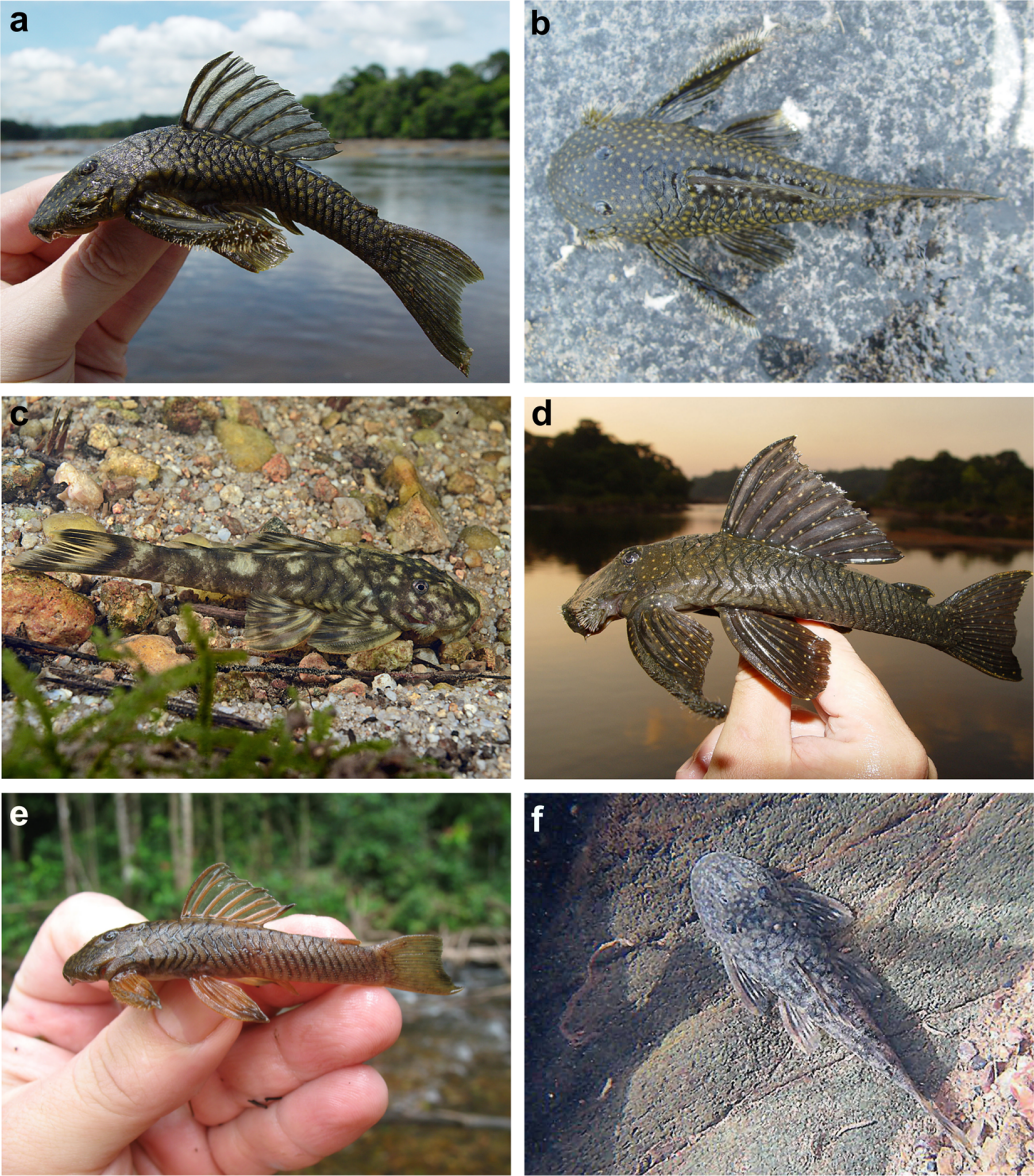
Live-color photographs of *Guyanancistus* spp. **a:** *G*. *longispinis*, MHNG 2680.100, French Guiana: Oyapock River at Alikoto (R. Covain); **b:** *G*. *longispinis*, MHNG 2680.100, French Guiana: Oyapock River at Alikoto (R. Covain); **c:** *G*. *megastictus*, French Guiana: Maroni River, Mitaraka Mountains, Alama Creek (F. Melki); **d:** *G*. *niger*, MHNG 2722.089, French Guiana: Oyapock River, Saut Maripa (R. Covain); **e**: *G*. *nassauensis*, Suriname, Marowijne River, Nassau Mountains, Paramaka Creek (J. W. Armbruster); **f:** *G*. *brownsbergensis*, Suriname: Saramacca River, Brownsberg Mountains, Irene Falls (K. Wan Tong You).

**Fig 7:**
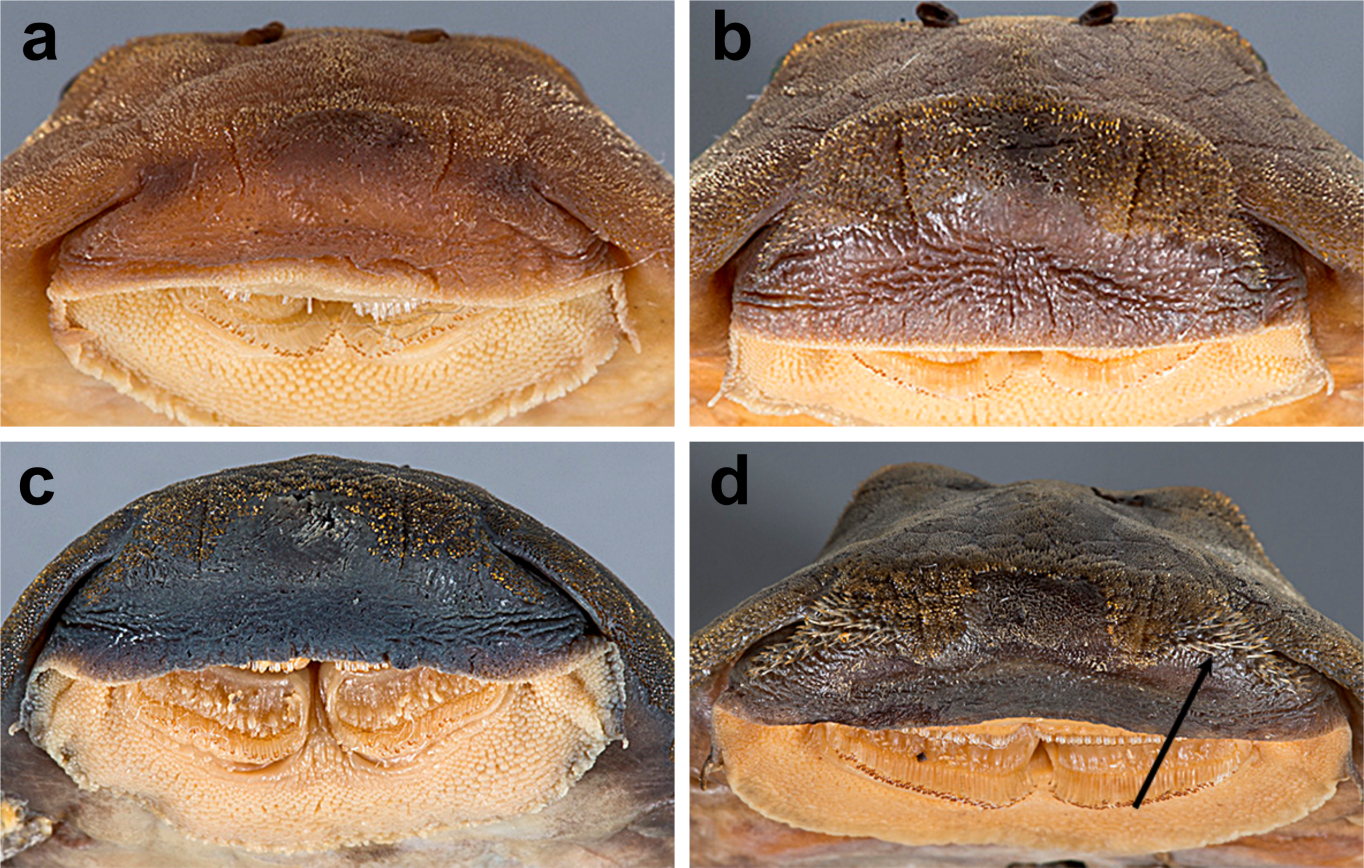
Anteroventral view of snout of *Guyanancistrus* members. *Guyanancistrus brevispinis*: **a:** MHNG 2108.014, paratype, 89.1 mm SL; **b:** MHNG 2673.034, 107.7 mm SL; **c:** MHNG 2723.007, 137.7 mm SL; *G*. *niger*: **d:** MHNG 2722.089, 158.5 mm SL. An arrow indicates enlarged odontodes on the anterodorsal edge of upper lip.

**Fig 8:**
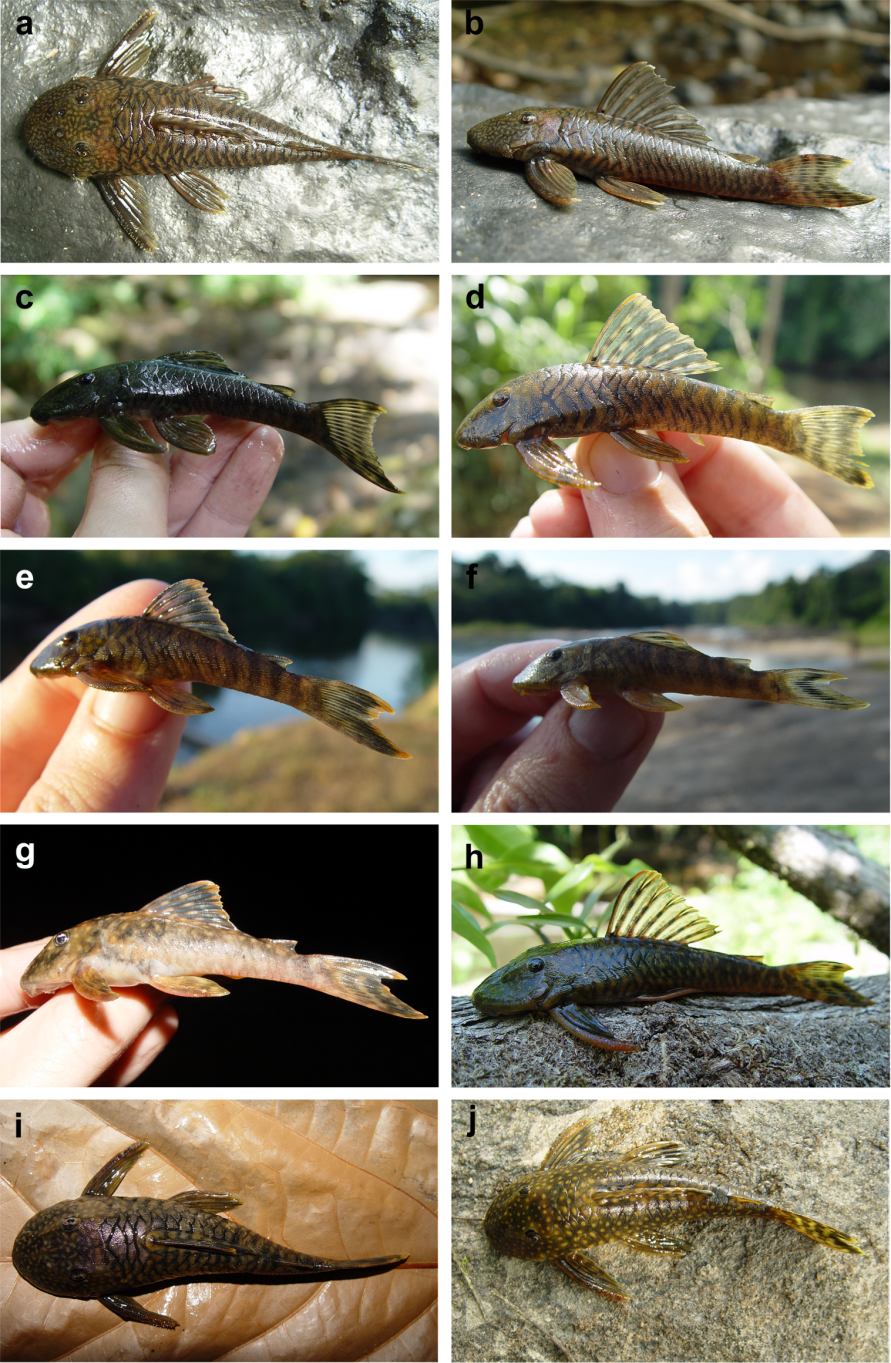
Live-color photographs of *Guyanancistrus brevispinis*. *G*. *b*. *brevispinis*: **a, b:** Upper Corantijn River, Sipaliwini River; **c:** Lower Corantijn River, Kabalebo River; **d:** Nickerie River, Moses Creek; **e:** Suriname River, Cajana Creek; **f:** Upper Marowijne River, Tapanahony River; *G*. *b*. *bifax*: **g:** Maroni River, Crique Voltaire; **h:** Mana River, Petit Laussat, Paratype MHNG 2734.090, GFSU12-145; *G*. *b*. *orientalis*: **i:** Orapu River, Crique Grillon; **j:** Oyapock River at Roche Mon Père; holotype MNHN 2017–0450, GF06-183. Photos R. Covain.

(Figs 6, 7 and 8; Table 4)

**Table 4:** Descriptive morphometrics and meristics of *Guyanancistrus brevispinis*. Morphometric data expressed as percents of standard length (SL) or head length (HL). Abbreviations of the different morphometric variables used in the multivariate and multi-table analyses are provided in square brackets. n: number of specimens measured. H: holotype. SD: standard deviation. Computed statistics included holotype.

	*Guyanancistrus brevispinis*						*Guyanancistrus b*. *brevispinis*						*Guyanancistrus b*. *bifax*						*Guyanancistrus b*. *orientalis*					
	(n = 195)						(n = 75)						(n = 76)						(n = 44)					
	H	Range			Mean	SD	H	Range			Mean	SD	H	Range			Mean	SD	H	Range			Mean	SD
Standard length [SL] (mm)	126.0	23.8	-	152.0	66.0	26.4	126.0	23.8	-	126.0	63.0	28.7	102.8	29.3	-	137.7	65.8	19.8	113.5	30.2	-	152.0	71.3	31.7
**Percents of standard length**																								
Total length [Ltot]	130.0	127.7	-	141.1	134.2	2.9	130.0	127.7	-	139.9	134.0	3.1	135.9	128.6	-	141.1	134.7	3.0	133.7	128.3	-	140.1	133.6	2.5
Predorsal length [Lpred]	42.5	39.2	-	47.2	43.1	1.7	42.5	39.6	-	47.2	43.3	1.6	41.4	40.4	-	46.7	43.3	1.6	39.6	39.2	-	46.3	42.6	1.9
Head length [HL]	32.2	29.8	-	39.6	34.7	2.0	32.2	30.0	-	39.6	34.9	2.2	32.7	31.3	-	38.9	35.0	1.7	31.0	29.8	-	38.0	34.1	2.3
Head depth at supraoccipital [hauteur]	17.9	14.1	-	19.6	16.3	0.9	17.9	14.1	-	19.6	16.3	1.1	15.8	14.7	-	18.7	16.3	0.7	15.1	14.7	-	17.7	16.2	0.7
Cleithral width [largeur]	32.8	29.1	-	34.2	31.2	1.0	32.8	29.5	-	34.2	32.0	0.9	30.3	29.2	-	33.2	30.7	0.7	29.9	29.1	-	32.4	30.8	0.8
Dorsal spine length [ep.D]	24.9	19.3	-	28.1	24.6	1.5	24.9	21.0	-	27.7	24.6	1.3	25.0	19.3	-	28.1	24.9	2.0	25.3	22.3	-	26.1	24.2	1.0
Dorsal–fin base length [base_D]	25.2	21.0	-	27.6	24.7	1.2	25.2	21.0	-	26.7	24.4	1.1	25.4	22.7	-	27.4	25.0	1.2	24.9	22.4	-	27.6	24.6	1.4
Interdorsal distance [interd]	21.4	14.9	-	21.4	17.7	1.2	21.4	14.9	-	21.4	17.5	1.3	19.6	15.5	-	20.0	17.8	1.0	20.6	15.6	-	20.8	18.0	1.2
Pectoral spine length [ep.P]	34.4	22.4	-	34.4	28.7	2.2	34.4	24.1	-	34.4	29.4	2.1	29.8	22.4	-	33.3	28.3	2.3	30.2	24.1	-	33.6	28.2	2.0
Pelvic spine length [ep.V]	26.6	21.8	-	27.4	24.7	1.1	26.6	22.3	-	26.9	24.8	1.1	25.2	21.8	-	27.4	24.5	1.2	25.6	22.9	-	27.2	24.8	1.0
Thoracic length [Lthor]	22.7	20.2	-	25.8	23.0	1.2	22.7	21.4	-	25.8	23.6	1.0	23.0	20.6	-	25.7	22.7	1.2	22.4	20.2	-	24.6	22.6	1.1
Abdominal length [Labd]	24.6	20.2	-	26.9	23.7	1.1	24.6	20.2	-	26.9	23.6	1.3	24.7	21.1	-	25.8	23.9	0.9	25.4	21.0	-	26.0	23.6	1.1
Caudal peduncle length [L_ped.C]	28.6	25.7	-	31.1	28.9	1.1	28.6	25.7	-	31.0	28.9	1.1	28.7	25.8	-	31.0	28.7	1.1	30.5	27.2	-	31.1	29.4	0.9
Caudal peduncle depth [H_ped.C]	11.0	8.6	-	11.3	10.0	0.6	11.0	9.7	-	11.3	10.5	0.4	9.3	8.6	-	10.7	9.4	0.5	10.3	9.2	-	11.0	10.0	0.4
Adipose spine length [ep.ad.]	8.8	5.5	-	10.9	9.0	0.8	8.8	5.5	-	10.9	9.2	0.9	9.0	6.2	-	10.3	8.8	0.8	9.1	6.8	-	10.2	8.9	0.7
Anal fin length [anale]	12.6	7.9	-	14.0	10.1	1.2	12.6	8.5	-	13.9	10.3	1.0	12.9	7.9	-	13.5	9.8	1.3	12.6	7.9	-	13.4	10.3	1.3
Upper caudal spine length [ep.sup.C]	24.2	21.6	-	29.8	25.3	1.7	24.2	21.6	-	29.1	25.1	1.8	28.1	22.3	-	28.4	25.6	1.6	26.6	21.9	-	29.8	25.1	1.6
Lower caudal spine length [ep.inf.C]	-	28.2	-	39.9	33.0	2.3	-	28.2	-	38.5	32.8	2.4	34.7	28.2	-	39.9	33.6	2.4	32.4	28.8	-	39.4	32.5	2.1
Body width at dorsal–fin origin [larg.base_D]	29.0	22.6	-	31.2	26.6	1.5	29.0	23.0	-	31.2	27.1	1.7	24.8	22.6	-	29.5	26.3	1.3	26.5	24.1	-	28.4	26.1	1.0
**Percents of head length**																								
Supracleithral width [larg.scl]	87.2	65.9	-	91.8	79.3	4.9	87.2	70.0	-	91.8	80.3	5.3	80.7	65.9	-	88.6	77.6	4.6	84.6	72.3	-	88.4	80.3	4.0
Snout length [museau]	65.0	52.9	-	68.5	62.1	2.7	65.0	52.9	-	68.5	61.1	3.2	64.0	55.4	-	68.4	62.2	2.1	65.7	58.0	-	66.9	63.3	2.2
Interorbital width [interorb]	35.0	27.7	-	38.6	32.3	2.0	35.0	29.2	-	36.9	32.8	1.9	34.7	27.7	-	36.1	31.8	1.7	34.7	28.6	-	38.6	32.4	2.5
Plated internostril distance [internostr]	9.4	7.5	-	14.1	10.8	1.3	9.4	8.7	-	14.1	10.9	1.3	12.6	8.9	-	14.0	11.0	1.1	10.8	7.5	-	12.8	10.2	1.2
Orbital diameter [orb]	15.8	14.8	-	22.8	18.6	1.3	15.8	15.3	-	21.3	18.5	1.5	17.5	15.3	-	22.8	18.8	1.2	18.2	14.8	-	21.2	18.3	1.3
Opercle length [op]	16.0	12.6	-	20.3	16.2	1.5	16.0	12.8	-	20.3	17.2	1.4	15.5	12.8	-	18.6	15.4	1.2	16.8	12.6	-	18.2	15.7	1.2
Dentary tooth cup length [isthme]	19.7	15.8	-	23.6	19.4	1.7	19.7	15.8	-	23.6	19.1	1.8	20.4	15.8	-	23.1	19.3	1.6	22.0	15.8	-	22.5	20.1	1.6
Premaxillary tooth cup length [mand]	19.7	16.2	-	24.5	19.9	1.7	19.7	16.2	-	23.3	19.5	1.9	20.0	16.3	-	24.5	19.9	1.7	21.8	17.7	-	24.5	20.6	1.3
Interbranchial distance [isthme]	71.4	54.4	-	73.3	64.4	3.5	71.4	54.4	-	73.3	65.4	4.0	64.5	56.4	-	67.9	62.8	2.7	66.7	60.2	-	71.7	65.6	2.8
**Counts**																								
Dentary teeth [md]	73	25	-	73	46	10	73	25	-	73	45	11	62	25	-	72	46	10	48	31	-	72	48	10
Premaxillary teeth [mx]	61	24	-	70	45	9.5	61	27	-	67	44	9.5	59	24	-	70	45	10	48	32	-	65	46	9
Lateral plates [l.lat]	24	22	-	26	24	10.5	24	22	-	25	24	0.5	24	23	-	25	24	0.5	24	23	-	26	24	0.5
Predorsal plates [pd]	3	2	-	4	3	0.5	3	2	-	4	3	0.5	3	2	-	4	3	0.5	3	2	-	4	3	0.5
Plates bordering supraoccipital [so]	3	2	-	3	3.0	0.5	3	3	-	3	3	0	3	2	-	3	3	0.5	3	2	-	3	3	0.5
Plates along dorsal–fin base [bd]	7	7	-	8	7.5	0.5	7	7	-	8	7.5	0.5	8	7	-	8	7	0.5	7	7	-	8	7.5	0.5
Plates between dorsal base and adipose fin [id]	8	5	-	8	6	0.5	8	5	-	8	6	0.5	6	5	-	7	6	0.5	6	5	-	7	6	0.5
Adipose to caudal plates [ac]	7	5	-	8	6.5	0.5	7	6	-	7	6	0.5	6	5	-	7	6	0.5	7	6	-	8	5	0.5
Plates between dorsal fin tip and adipose fin [d.a]	1.5	0.5	-	3	2	0.5	1.5	0.5	-	3	2	0.5	2	1	-	3	2	0.5	2	1	-	3	2	05
Plates along adipose-fin base [ba]	1	1	-	4	2	0.5	1	1	-	4	2	0.5	1.5	1.5	-	3	2	0.5	1	1	-	2.5	2	0.5
Anal to caudal plates [ps]	11	10	-	12	11	0.5	11	10	-	12	11	0.5	11	10	-	11	11	0.5	11	10	-	11	11	0.5
Branched dorsal-fin rays [D]	7	7	-	8	7	0.5	7	7	-	7	7	0	7	7	-	7	7	0	7	7	-	8	7	1
Branched anal-fin rays [A]	5	4	-	5	5	1	5	5	-	5	5	0	5	4	-	5	5	1	5	4	-	5	5	1

*Lasiancistrus brevispinis* Heitmans, Nijssen & Isbrücker, 1983[62]: 38, Figs 4–7 (type locality: Surinam, district Nickerie [Sipaliwini], Fallawatra River, rapid 5 km S.W. of Stondansie Fall, Nickerie River system; holotype: ZMA 107.740); Ouboter & Mol, 1993 [58]: 149 (distribution in Suriname); Boujard et al., 1997 [103]: 183; Le Bail et al., 2000 [59]: 236–237 (complementary description, distribution in French Guiana, illustration);

*Guyanancistrus brevispinis* (Heitmans, Nijssen & Isbrücker, 1983): Isbrücker in Isbrücker et al., 2001 [52]: 19 (original designation as type species of *Guyanancistrus*); Fisch-Muller, 2003 [100]: 385 (checklist, valid); Mol et al. 2007 [56]: 112 (Lely Mountains); Covain & Fisch-Muller, 2012 [50]: 244 (in molecular phylogeny of *Pseudancistrus sensu lato*, generic reassignation); Mol et al., 2012 [45]: 274 (distribution in Suriname); Le Bail et al., 2012 [44]: 303 (distribution in French Guiana); Mol, 2012 [55]: 448–449 (complementary description and illustration); Lujan et al., 2015 [49]: 278 (in a molecular phylogeny of Loricariidae); Melo et al., 2016 [104]: 134 (collected in Amapá, Brazil).

*Lasiancistrus niger* (not of Heitmans, Nijssen & Isbrücker, 1983): Montoya-Burgos et al., 1998 [105]: 367 (in a molecular phylogeny of Loricariidae)

*Pseudancistrus brevispinis* (Heitmans, Nijssen & Isbrücker, 1983): Armbruster, 2004a [53] (in a morphological phylogeny), 2004b [54] (illustration, Fig 2A), 2008 [106]; Ferraris, 2007 [47]: 287 (checklist); Cardoso & Montoya-Burgos, 2009 [60]: 947 (diversity and historical biogeography); Willink et al., 2010 [107]: 41 (comparison to *P*. *kwinti*).

**Diagnosis.** *Guyanancistrus brevispinis* is discriminated from all congeners except *G*. *nassauensis* n. sp. by specific barcode sequences (see BOLD numbers in Table 1) and by much shorter evertible cheek odontodes, longest ones usually only reaching the first half of the opercle, or, in some large specimens measuring more than 70 mm SL (probably adult males), surpassing the middle of the opercle, but not reaching its last quarter (*vs* reaching between last quarter up to far beyond posterior end of opercle except in very small specimens). Evertible cheek odontodes are shorter in *G*. *brevispinis* than in *G*. *nassauensis* with regard to size of specimens; in the latter, large specimens (likely adult males) that have odontodes reaching beyond the middle of the opercle measure only 40 mm. *Guyanancistrus brevispinis* is a larger species than *G*. *nassauensis* (maximum known SL: 152 mm *vs* 61 mm). It is further discriminated from *G*. *nassauensis* by smaller dentary and premaxillary tooth cusps (in % of head length, respectively: 15.8–23.6, mean 19.5, *vs* 24.2–31.9, mean 27.6; 16.3–24.5, mean 20.1, *vs* 25.4–31.4, mean 28.1) and by an anal fin with 5 branched rays (*vs* 4), apart exceptional individuals.

*Guyanancistrus brevispinis* is readily distinguished from *G*. *longispinis* and *G*. *niger* by color pattern (body and fins medium brown with paler yellow to orange medium-sized spots to transverse bands, *vs* brown-black with either small roundish yellow spots for *G*. *longispinis* (Fig 6A and 6B), or white dots for *G*. *niger* (Fig 6D)). *Guyanancistrus brevispinis* can also be distinguished from *G*. *brownsbergensis* and *G*. *tenuis* by a smaller number of plates between adpressed dorsal fin and adipose-fin spine (0.5–3, mean 2, *vs* respectively 3–3.5, mean 3, and 3–4, mean 3.5), from *G*. *brownsbergensis* by a lower peduncle depth (8.6–11.3, mean 10.0% of SL, *vs* 11.4–11.6, mean 11.5), and from *G*. *megastictus* by the lower number of plates bordering the supraoccipital (2–3, mean 3 *vs* 4) and by a color pattern with smaller roundish spots (not covering four plates) or straighter bands on posterior part of body and fins.

**Description.** Morphometric and meristic data in Table 4. Relatively large-sized species for *Guyanancistrus* (up to 152 mm SL). Head and body dorsoventrally depressed. Dorsal profile gently convex from snout tip to dorsal-fin origin, usually more flattened posterior to orbit, slightly convex and sloped ventrally from dorsal-fin origin to adipose fin, then slightly concave to procurrent caudal-fin rays, and rising to caudal fin. Ventral profile flat from snout to base of caudal fin.

Dorsal contour of head smooth, no ridge or keel, inconspicuous rounded elevations on the midline of the snout and anterior to orbits, supraoccipital nearly flat. Dorsal margin gently flattened from base of first branched dorsal-fin ray to base of adipose fin between very slight ridges formed with lateral plates of dorsal series. First lateral plates of mid-ventral series forming low lateral ridge. Caudal peduncle roughly ovoid in cross section, flattened ventrally, and more compressed posteriorly.

Snout rounded anteriorly. Eye moderately large. Lips forming an oval disk, covered with short papillae. Presence of a single narrow buccal papilla. Lower lip wide, not reaching pectoral girdle, upper lip narrower. Very short maxillary barbel. Teeth bicuspid, lateral lobe about half size of medial lobe.

Head and body plated dorsally, plates generally covered by short and uniformly distributed odontodes. Tip of snout always naked, and, except in some large specimens, small naked area meeting the dorsolateral edge of upper lip on each side of tip of snout; in large specimens, dorsolateral margin of the upper lip supporting from few odontodes up to several plates covered with small odontodes (Fig 7A, 7B and 7C). Lateral margin of snout covered with plates forming a rigid armor with short odontodes. Opercle supporting odontodes. A narrow unplated area bordering posterodorsal margin of opercle. Evertible cheek plates with enlarged odontodes in highly variable number, from less than ten up to approximately 40 in some large specimens. These cheek odontodes straight with tips curved, longest reaching from the first quarter (in small specimens) up to the third quarter (in large specimens) of the opercle. Usually three rows of plates and a curved nuchal plate between supraoccipital plate and dorsal-fin spinelet. Five series of lateral plates extending to caudal fin. Odontodes on lateral series of plates not forming keels. Odontodes on posterior part of pectoral-fin spine only slightly enlarged, except in large specimens (presumably males). Abdominal region totally naked. No platelike structure before the anal fin. Ventral part of caudal peduncle plated; presence of a large smooth area devoid of odontodes around anal fin.

Dorsal-fin origin slightly anterior to pelvic-fin origin. Dorsal fin relatively short; when adpressed, never reaching adipose fin, often very distant from it. Adipose fin roughly triangular, preceded by one (or two fused into one) median unpaired raised plate. Adipose spine straight or slightly convex dorsally, membrane posteriorly convex. Pectoral-spine tip reaching to approximately one-third of pelvic spine in most specimens, exceptionally extending over its middle in large specimens (presumably males). Anal fin with weak spine, its margin convex. Caudal fin concave, ventral lobe longer than dorsal lobe. Fin-ray formulae: dorsal II,7; pectoral I,6; pelvic i,5; anal i,5 (except 3 specimens with I,4); caudal i,14, i.

**Coloration.** Dorsal coloration pattern highly variable according to size of specimen and collection locality (see Fig 8 and discussion). In life, base color light grey-brown or orange-brown to dark brown, except whitish ventral region. Dorsal spotting pattern varies from presence of indistinct paler spots, to presence of numerous small to medium-sized, roundish to elongated, faded to brilliant yellow-orange spots on the whole body, or on its anterior part only; in that case spots form similarly colored transverse bands on posterior part of body. Such bands are observed on juveniles of all populations, and remain on larger specimens in some populations.

Spots of similar color to those of body are usually present on dorsal and caudal fins, centred on fin rays and often combined to form regular or irregular transverse bands, more generally on caudal fin. Pectoral and pelvic fins with less-distinct spots, sometimes with large pale areas. Fin margins can be of same color as spots, especially in small specimens. Fin membranes usually grayish-brown.

**Distribution and habitat.** *Guyanancistrus brevispinis* was found in the main Guianese river systems of Suriname and French Guiana, including the Corantijn at Guyanese-Surinamese border, the Nickerie, Coppename, Saramacca, Suriname, Maroni, Mana, Sinnamary, Mahury (Comté-Orapu), Kaw, Approuague and the Oyapock at the French-Brazilian border. These rivers all have a south-north orientation and flow into the Atlantic Ocean. It inhabits most clear-water rivers, streams and creeks, with running waters and rocky or sandy bottoms, over which specimens blend remarkably well. The species may be locally abundant. Its habitat is usually shadowed by primary rainforest, with measured water temperature varying from 23.7 to 28.4°C, conductivity from 13 to 42 us and pH from 5.96 to 7.06.

**Etymology.** The specific name *brevispinis*, derived from Latin and meaning short thorns, was originally given in reference to the short evertible cheek odontodes.

#### *Guyanancistrus brevispinis brevispinis* (Heitmans, Nijssen & Isbrücker, 1983)

(Fig 8A, 8B, 8C, 8D, 8E and 8F; Table 4)

**Holotype.** Same as nominal species: ZMA 107.740, 126 mm SL*; Surinam, Nickerie River system, district Sipaliwini [not Nickerie], Fallawatra River, rapid 5 km SW of Stondansie Fall; Nijssen, 6 April 1967.

**Non type material.** See S1 Text.

**Diagnosis.** The nominal subspecies of *G*. *b*. *brevispinis* is differentiated from the subspecies *bifax* and *orientalis* by characteristic barcode sequences (see BOLD numbers in Table 1). No morphometric variable strictly distinguishes *brevispinis* from the two other subspecies (see Table 4), but most mean values are significantly different from the other subspecies. These variables show that in comparison to them, on average, the body of *brevispinis* is significantly wider (cleithral width in % of SL: 31.98 ± 0.89% *vs* 30.75 ± 0.69 for *bifax* and 30.85 ± 0.75 for *orientalis*; p-value 3.498e^-16^) (width at dorsal-fin origin in % of SL: 27.11 ± 1.66% *vs* 26.26 ± 1.28 for *bifax* and 26.13 ± 1.00 for *orientalis*; p-value 0.0004472); on average, the interbranchial distance is larger (in % of SL: 22.73 ± 0.97% *vs* 21.91 ± 0.65 for *bifax* and 22.22 ± 0.95 for *orientalis*; p-value 1.624e^-7^), the interorbital wider (in % of SL: 11.41 ± 0.40 *vs* 11.09 ± 0.44 for *bifax* and 10.98 ± 0.35 for *orientalis*; p-value 3.15e^-7^), the exposed part of opercle longer (in% of SL: 6.00 ± 0.64 *vs* 5.39 ± 0.53 for *bifax* and 5.34 ± 0.50 for *orientalis*; p-value 3.482e^-10^), the thoracic length greater (in % of SL: 23.63 ± 1.01 *vs* 22.70 ± 1.15 for *bifax* and 22.59 ± 1.07 for *orientalis*; p-value 8.626e^-8^), and the caudal peduncle deeper (in % of SL: 10.50 ± 0.40 *vs* 9.45 ± 0.47 for *bifax* and 9.97 ± 0.40 for *orientalis*; p-value 2.2e^-16^). Also, *brevispinis* generally has fewer plates between adpressed dorsal-fin tip and adipose spine (1.5 ± 0.5 *vs* 2 ± 0.5 for *bifax* and 2 ± 0.5 for *orientalis*; p-value 3.484e^-6^).

**Distribution.** The nominate subspecies occurs from the Corantijn River basin in western Suriname to the upper Tapanahony River basin, a Marowijne River tributary in eastern Suriname. It was not found in French Maroni River tributaries (Fig 4).

**Etymology.** As for nominal species.

#### *Guyanancistrus brevispinis bifax* new subspecies

**Fig 9:**
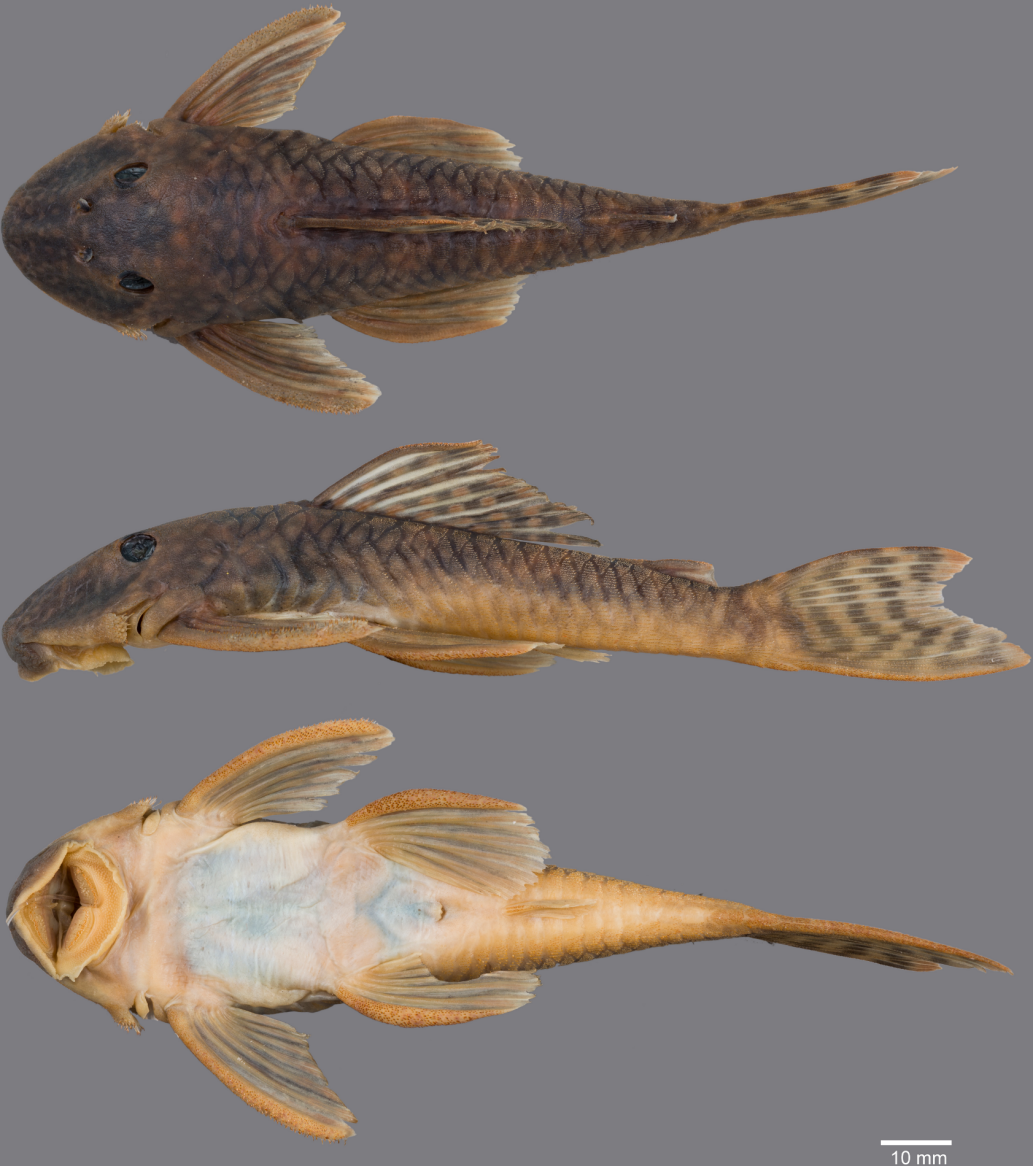
*Guyanancistrus brevispinis bifax*. MNHN 2017–0448, holotype, 102.8 mm SL; French Guiana: Crique Petit Laussat, right tributary of Mana River.

**Fig 10:**
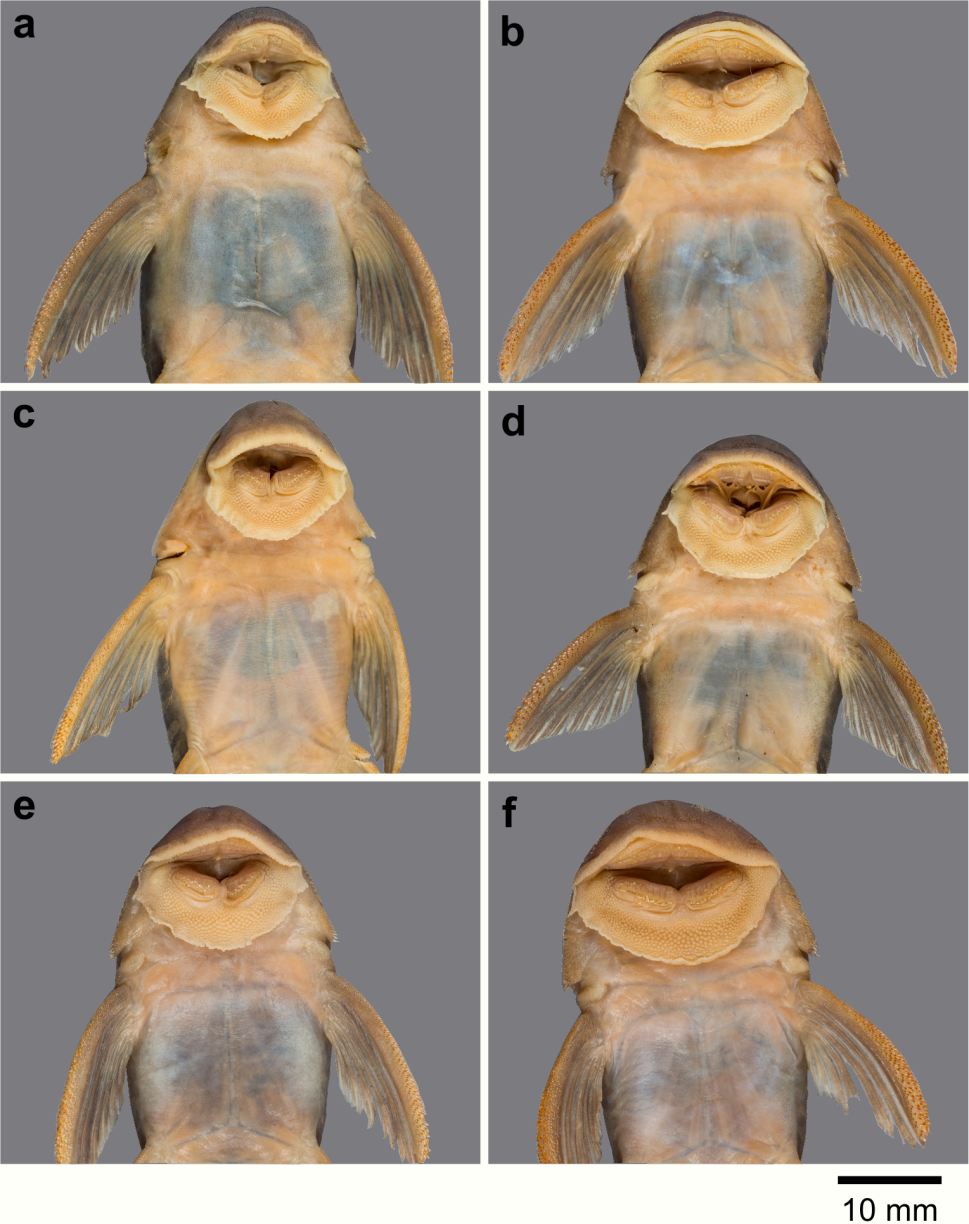
Mouth variation within *Guyanancistus brevispinis bifax*. **a**, MHNG 2683.029, female, 69.7 mm SL, and **b**, MHNG 2683.043, male, 70.8 mm SL, both specimens from Maroni River Basin, Crique Voltaire; **c**, MHNG 2683.050, female, 71.4 mm SL, and **d**, MHNG 2699.060, male, 68.4 mm SL, both specimens from Mana River Basin; **e**, MHNG 2723.009, female, 67.1 mm SL, and **f**, MHNG 2723.008, female, 71.5 mm SL, both specimens from Sinnamary River Basin, Crique Maïpouri.

urn:lsid:zoobank.org:act: 42111DB4-A0EA-4AC8-88DE-3A0BCE878091

(Figs 8G, 8H, 9 and 10; Table 4)

**Holotype.** MNHN 2017–0448 (ex MHNG 2734.090; GFSU12-140), 102.8 mm SL; French Guiana: Crique Petit Laussat, right tributary of Mana River (05°24'28.6"N 53°34'53.6"W); Covain & Fisch-Muller, 24 Oct. 2012.

**Paratypes.** MHNG 2734.090 (GFSU12-141), 21; MNHN 2017–0449, 4; (11 measured, 67.0–106.5 mm SL), same data as holotype. MNHN 2015–227, 3, Mana River, Saut Fracas; Le Bail et al., 21 Sep. 1999. MHNG 2593.087, 5; MNHN 2015–221, 3; (4 measured, 46.8–96.3 mm SL) Grand Inini River, Saut “S”, right tributary of Maroni River; Le Bail et al., 1 Oct. 1997.

**Non type material.** See S1 Text.

**Diagnosis.** *Guyanancistrus brevispinis bifax* is differentiated from other subspecies and species of *Guyanancistrus* by its barcode sequences (see BOLD numbers in Table 1), which are however not unique as they are partly shared by *Guyanancistrus nassauensis*. It is distinguished from the latter by the diagnostic characters listed under species diagnosis. No morphometric variable unambiguously distinguishes *bifax* from other subspecies (see Table 4), but several have significantly different mean values. Compared to both *brevispinis* and *orientalis*, on average, *bifax* has a smaller interbranchial distance (in % of SL: 21.91 ± 0.65 *vs* 22.73 ± 0.97 for *brevispinis* and 22.22 ± 0.95 for *orientalis*; p-value 1.624e^-8^), a more depressed caudal peduncle (in % of SL: 9.45 ± 0.47*vs* 10.50 ± 0.40 for *brevispinis* and 9.97 ± 0.40 for *orientalis*; p-value 2.2e^-8^), and a longer caudal fin (lower caudal-fin spine in % of SL: 33.63 ± 2.38 *vs* 32.79 ± 2.36 for *brevispinis* and 32.53 ± 2.11 for *orientalis*; p-value 0.00684). Additional differences of morphometric variables from either *brevispinis* or *orientalis* are statistically significant, some being listed under the respective diagnoses of these subspecies. On average, the number of plates between dorsal base and adipose fin is smaller for *bifax* (6. ± 0.5 *vs* 6 ± 1 for *brevispinis* and 6 ± 0.5 for *orientalis*; p-value 0.01807).

**Remark**: In several populations of the subspecies throughout its area of distribution, the head of some specimens (MHNG 2683.043; MHNG 2699.060; MHNG 2723.008) has a distinctive appearance, with an enlarged forehead part (Fig 10), usually coupled with a slightly longer snout, head and/or predorsal length, as well as an enlarged mouth. This difference between individuals appears not linked to sex, and apparently independant of the genetic data examined.

**Distribution.** *Guyanancistrus b*. *bifax* occurs from the Maroni River and its eastern tributaries up to the Mana and Sinnamary rivers basins in French Guiana (Fig 4).

**Etymology.** Named *bifax*, a noun in apposition, meaning two faces, for the different appearances of the head observed within the subspecies (see Remark).

#### *Guyanancistrus brevispinis orientalis* new subspecies

**Fig 11:**
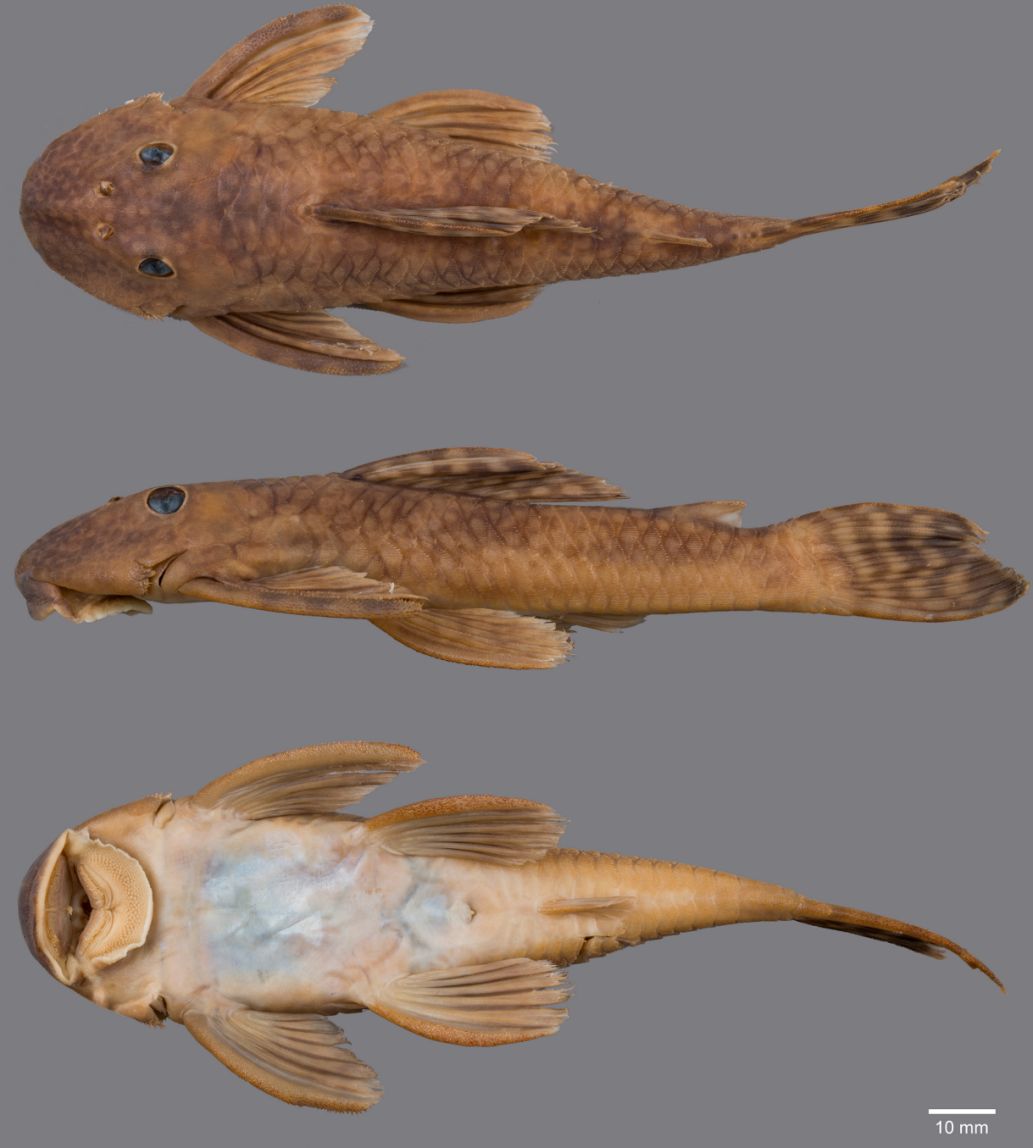
*Guyanancistrus brevispinis orientalis*. MNHN 2017–0450, holotype, 113.5 mm SL; French Guiana: forest creek, left tributary of Upper Oyapock River.

urn:lsid:zoobank.org:act: 116ABD70-36B3-4AB1-B35D-DDFC9ADC8CFE

(Figs 8I, 8J and 11; Table 4)

**Holotype.** MNHN 2017–0450 (ex MHNG 2681.098; GF06 183), 113.5 mm SL; French Guiana: forest creek, left tributary of Upper Oyapock River, in front of Roche Mon Père (03°16'56.3"N 52°12'36.6"W); Fisch-Muller et al., 6 Nov. 2006.

**Paratypes.** MHNG 2681.098, 1 juvenile (measured, 33.4 mm SL), same data as holotype; MHNG 2723.012, 5; MHNG 2723.013, 5; MNHN 2015–225, 5; (8 measured, 32.2–122.1mm SL) first rapids of Crique Gabaret, left tributary of Lower Oyapock River; Fisch-Muller et al. 21 Oct. 1999.

**Non type material.** See S1 Text.

**Diagnosis.** The subspecies *orientalis* is differentiated from other subspecies of *G*. *brevispinis* by characteristic barcode sequences (see BOLD numbers in Table 1). No morphometric variable unambiguously distinguishes *orientalis* from the two other subspecies, but several have significantly different mean values (see Table 4). On average, *Guyanancistrus brevispinis orientalis* has a smaller internostril distance (in % of SL: 3.47 ± 0.48 *vs* 3.82 ± 0.54 for *brevispinis* and 3.85 ± 0.46 for *bifax*; p-value = 8.527e^-5^), and a longer caudal peduncle (in % of SL: 29.42 ± 0.93 *vs* 28.87 ± 1.06 for *brevispinis* and 28.68 ± 1.09 for *bifax*; p-value = 0.001369). It also has fewer plates along adipose-fin base (mean 1.5 ± 0.5 *vs* 2 ± 0.5 for *brevispinis* and 2 ± 0.5 for *bifax*; p-value = 0.03897). Its interbranchial distance is larger than for *bifax* but smaller than for *brevispinis* (mean in % of SL: 22.22 ± 0.95 *vs* respectively 21.81 ± 0.65 and 22.73 ± 0.97; p-value = 1.624e^-7^), and its caudal peduncle is deeper than for *bifax* but lower than for the nominal subspecies (mean in % of SL: 9.97 ± 0.40 *vs* respectively 9.45 ± 0.47 and 10.50 ± 0.40; p-value = 2.2e^-16^). Additional differences of morphometric variables from either *brevispinis* or *bifax* are statistically significant, some being listed under the respective diagnoses of these subspecies.

**Distribution.** *Guyanancistrus brevispinis orientalis* is distributed in eastern French Guiana, from the Mahury (Comté-Orapu) River Basin to the Approuague and Oyapock river basins (Fig 4).

**Etymology.** The name *orientalis*, from the Latin name *oriens*, is given because of the eastern distribution of the subspecies.

#### *Guyanancistrus nassauensis* Mol, Fisch-Muller & Covain, new species

**Fig 12:**
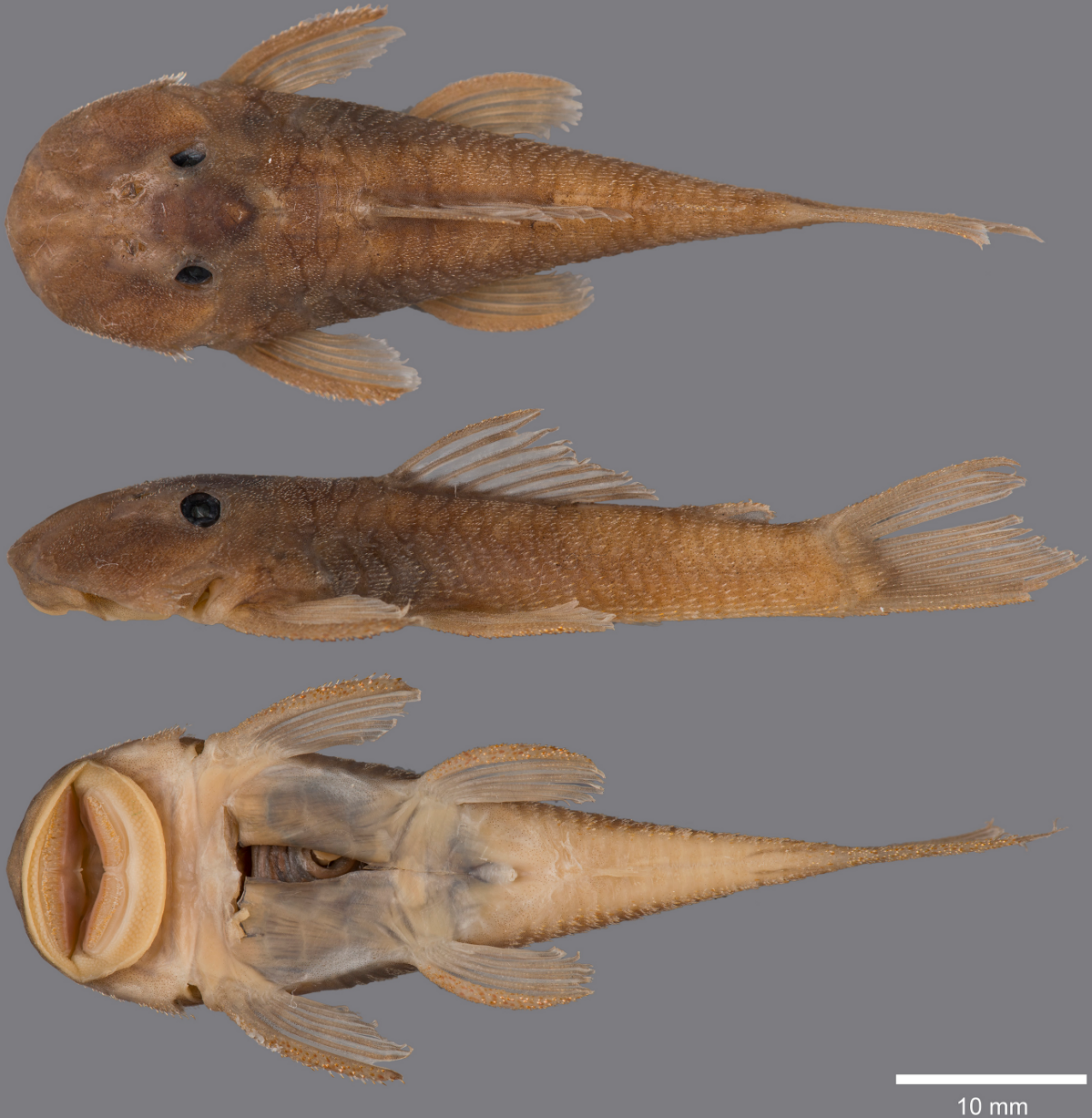
*Guyanancistrus nassauensis*. MHNG 2679.100, holotype, 42.0 mm SL; Suriname: Sipaliwini: Paramaka Creek, Nassau Mountains.

urn:lsid:zoobank.org:act: 523943AF-5A39-4A33-B783-0A7DA9A3AD0D

(Figs 6E and 12; Table 5)

**Table 5:** Descriptive morphometrics and meristics of *Guyanancistrus* spp. and *Cryptancistrus similis*. Morphometric data expressed as percents of standard length (SL) or head length (HL). Abbreviations of the different morphometric variables used in the multivariate and multi-table analyses are provided in square brackets. n: number of specimens measured. H: holotype. SD: standard deviation. Computed statistics included holotype.

	*Guyanancistrus nassauensis*						*Guyanancistrus brownsbergensis*						*Guyanancistrus teretisrostris*						*Guyanancistrus tenuis*						*Guyanancistrus megastictus*				*Cryptancistrus similis*
	(n = 23)						(n = 3)						(n = 3)						(n = 15)						(n = 2)				(n = 1)
	H	Range			Mean	SD	H	Range			Mean	SD	H	Range			Mean	SD	H	Range			Mean	SD	H	P	Mean	SD	H
Standard length [SL] (mm)	42.0	22.9	-	61.0	40.6	8.4	63.8	49.2	-	63.8	56.2	7.3	97.6	87.0	-	97.6	94.0	6.1	90.9	33.2	-	90.9	64.5	17.9	62.7	57.1	59.9	4.0	61.7
**Percents of standard length**																													
Total length [Ltot]	133.7	125.0	-	137.2	132.1	3.3	131.0	131.0	-	135.1	133.6	2.2	133.2	129.1	-	133.2	131.3	2.0	130.8	128.6	-	136.3	132.0	2.7	137.1	136.4	136.8	0.5	134.8
Predorsal length [Lpred]	44.1	40.3	-	47.0	44.3	1.5	42.0	41.5	-	42.6	42.0	0.6	41.5	39.8	-	41.5	40.8	0.8	40.2	39.1	-	45.3	41.5	1.7	42.0	40.6	41.3	1.0	43.7
Head length [HL]	36.1	32.2	-	40.7	36.4	2.1	33.4	33.2	-	33.6	33.4	0.2	32.3	31.7	-	32.3	31.9	0.3	31.4	30.8	-	35.8	32.5	1.5	32.4	31.8	32.1	0.4	35.0
Head depth at supraoccipital [hauteur]	16.0	16.0	-	18.1	17.1	0.6	15.2	15.2	-	15.7	15.5	0.3	15.3	13.8	-	15.3	14.7	0.8	13.5	13.5	-	14.8	14.0	0.4	15.1	13.9	14.5	0.8	16.0
Cleithral width [largeur]	34.0	32.2	-	36.6	34.3	1.1	31.5	31.5	-	31.7	31.6	0.1	31.1	29.7	-	31.1	30.5	0.7	29.4	27.9	-	31.7	30.1	0.8	32.7	31.8	32.2	0.6	33.0
Dorsal spine length [ep.D]	19.9	19.9	-	23.9	21.7	1.1	-	24.3	-	25.6	25.0	0.9	23.5	23.0	-	23.5	23.3	0.2	23.3	22.2	-	25.3	24.0	1.0	26.1	24.5	25.3	1.1	26.0
Dorsal–fin base length [base_D]	23.3	21.8	-	27.4	24.1	1.4	23.4	23.1	-	24.5	23.7	0.8	25.1	23.9	-	25.1	24.6	0.7	22.8	20.7	-	24.5	22.7	1.0	24.0	22.9	23.5	0.8	26.0
Interdorsal distance [interd]	16.2	13.5	-	20.4	16.9	1.7	20.0	19.4	-	20.9	20.1	0.7	17.8	17.8	-	19.4	18.6	0.8	20.7	18.3	-	21.5	20.1	1.0	19.8	19.8	19.8	0.0	16.5
Pectoral spine length [ep.P]	23.8	22.2	-	26.3	24.4	1.1	28.7	27.7	-	29.7	28.7	1.0	29.7	27.4	-	29.7	28.5	1.2	27.4	26.0	-	28.1	27.0	0.6	28.1	28.3	28.2	0.2	28.4
Pelvic spine length [ep.V]	22.7	20.0	-	24.6	22.7	1.2	26.2	25.0	-	26.7	26.0	0.9	23.5	21.5	-	23.5	22.5	1.0	24.5	23.5	-	26.1	24.8	0.9	26.3	24.6	25.5	1.2	25.3
Thoracic length [Lthor]	23.3	21.5	-	24.9	22.7	0.9	22.6	22.6	-	24.4	23.6	0.9	22.7	22.7	-	24.1	23.3	0.7	25.2	23.6	-	26.8	25.1	1.0	25.4	23.9	24.6	1.1	25.8
Abdominal length [Labd]	23.6	20.5	-	24.5	22.9	1.1	23.1	21.8	-	23.1	22.5	0.6	23.5	23.5	-	24.7	24.1	0.6	23.5	20.8	-	24.6	22.6	1.0	24.4	22.5	23.4	1.3	23.9
Caudal peduncle length [L_ped.C]	27.0	26.4	-	32.5	28.3	1.4	28.0	28.0	-	29.1	28.5	0.5	30.7	29.4	-	30.7	29.9	0.7	30.1	29.4	-	32.8	31.1	1.0	31.0	31.6	31.3	0.4	28.5
Caudal peduncle depth [H_ped.C]	11.2	10.5	-	12.6	11.5	0.5	11.4	11.4	-	11.6	11.5	0.1	10.5	10.5	-	10.8	10.6	0.2	8.9	8.9	-	9.6	9.3	0.2	10.9	10.4	10.7	0.3	11.5
Adipose spine length [ep.ad.]	9.0	7.1	-	10.3	9.1	0.7	7.9	7.9	-	8.1	8.0	0.1	8.4	7.3	-	8.5	8.1	0.7	6.3	6.3	-	8.6	8.0	0.6	9.5	9.0	9.3	0.4	8.8
Anal fin length [anale]	8.2	7.2	-	10.3	8.5	0.9	12.8	12.0	-	12.8	12.2	0.4	11.3	10.2	-	11.3	10.8	0.6	10.6	9.1	-	11.0	10.1	0.5	10.0	11.5	10.7	1.0	10.1
Upper caudal spine length [ep.sup.C]	25.0	19.2	-	25.5	23.3	1.9	26.4	26.4	-	27.2	26.8	0.4	23.9	23.5	-	23.9	23.7	0.2	24.4	24.2	-	27.0	25.5	0.8	27.4	25.8	26.6	1.1	-
Lower caudal spine length [ep.inf.C]	31.8	28.5	-	34.2	31.4	1.9	30.6	30.6	-	33.7	32.5	1.6	31.4	29.9	-	31.4	30.5	0.8	29.4	28.0	-	31.9	29.6	1.4	33.4	34.0	33.7	0.4	30.2
Body width at dorsal–fin origin [larg.base_D]	26.3	24.4	-	28.5	26.7	1.1	26.3	26.3	-	27.2	26.6	0.5	28.3	26.2	-	28.3	27.1	1.1	26.5	24.3	-	27.1	25.7	0.8	28.5	27.2	27.8	1.0	28.7
**Percents of head length**																													
Supracleithral width [larg.scl]	81.0	72.9	-	88.8	81.4	4.2	84.3	84.3	-	86.5	85.6	1.2	84.5	82.2	-	85.4	84.0	1.7	82.7	77.8	-	87.6	82.4	2.9	89.4	89.5	89.4	0.0	83.9
Snout length [museau]	65.0	60.2	-	66.7	64.1	1.4	63.0	61.3	-	63.0	62.4	1.0	65.9	65.2	-	65.9	65.6	0.4	64.5	59.5	-	64.5	61.5	1.4	62.9	61.2	62.1	1.2	59.0
Interorbital width [interorb]	30.7	29.0	-	34.4	31.3	1.2	36.4	36.4	-	38.3	37.4	1.0	37.3	35.9	-	37.3	36.5	0.7	34.4	31.3	-	35.7	33.4	1.3	37.2	36.7	37.0	0.3	36.4
Plated internostril distance [internostr]	9.3	8.2	-	10.9	9.7	0.7	10.7	10.3	-	10.7	10.6	0.2	10.8	9.9	-	10.8	10.4	0.5	11.4	9.1	-	12.4	10.5	0.9	12.0	11.2	11.6	0.5	10.8
Orbital diameter [orb]	15.7	15.1	-	17.4	16.4	0.7	15.1	15.1	-	17.0	16.0	0.9	15.6	15.6	-	16.1	15.8	0.3	16.7	16.7	-	19.7	17.8	0.7	16.6	16.5	16.6	0.0	19.0
Opercle length [op]	15.5	13.3	-	23.3	16.7	2.1	16.2	15.1	-	16.2	15.5	0.6	15.9	15.9	-	18.0	16.8	1.1	15.5	13.9	-	17.8	16.1	1.1	18.1	20.2	19.1	1.5	16.4
Dentary tooth cup length [isthme]	28.3	24.2	-	31.9	27.6	1.9	20.5	19.8	-	20.5	20.0	0.4	20.1	19.5	-	20.2	19.9	0.4	20.6	15.8	-	20.6	17.4	1.3	16.8	16.8	16.8	0.0	18.9
Premaxillary tooth cup length [mand]	29.3	25.4	-	31.4	28.1	1.6	22.5	21.2	-	22.5	21.6	0.7	21.2	18.1	-	21.2	19.9	1.6	21.1	15.1	-	21.1	18.0	1.5	17.4	17.8	17.6	0.3	17.7
Interbranchial distance [isthme]	76.8	63.6	-	79.0	72.2	3.5	68.2	68.2	-	71.7	69.7	1.8	66.6	66.6	-	69.6	67.8	1.6	67.3	63.7	-	69.4	65.7	1.8	69.6	68.9	69.3	0.5	60.2
**Counts**																													
Dentary teeth [md]	67	43	-	77	59	9	46	35	-	46	41	6	37	37	-	48	44	6	38	29	-	58	44	9	45	54	50	6	48
Premaxillary teeth [mx]	59	44	-	67	57	6	45	40	-	45	43	3	40	36	-	45	40	5	35	31	-	68	46	11	48	57	53	6	51
Lateral plates [l.lat]	24	23	-	25	24	0.5	25	24	-	25	25	0.5	25	25	-	25	25	0	24	24	-	26	25	0.5	24	23	24	1	24
Predorsal plates [pd]	3	2	-	4	3	0.5	3	3			3	0	3	3			3	0	3	3	-	4	3	0.5	3	3	3	0	3
Plates bordering supraoccipital [so]	3	2	-	4	3	0.5	3	3			3	0	4	3	-	4	3.5	0.5	5	3	-	5	4.5	0.5	4	4	4	0	3
Plates along dorsal–fin base [bd]	7	7	-	8	7.5	0.5	7	7			7	0	7	7	-	8	7.5	0.5	7	7			7	0	7	7	7	0	8
Plates between dorsal base and adipose fin [id]	6	5	-	7	6	0.5	7	7			7	0	6	6	-	7	6.5	0.5	6	6	-	7	7	0.5	6	6	6	0	6
Adipose to caudal plates [ac]	6	6	-	7	6.5	0.5	7	7			7	0	8	6	-	8	7	1	7	6	-	8	7	1	8	7	8	1	6
Plates between dorsal fin tip and adipose fin [d.a]	2.5	2	-	3.5	2.5	0.4	3	3	-	3.5	3	0.5	2	2	-	3	3	0.5	3.5	3	-	4	3.5	0.5	2.5	3	3	0.5	0.5
Plates along adipose-fin base [ba]	1.5	1.5	-	2.5	2	0.5	2	1.5	-	2	2	0.5	1	1	-	1.5	1.5	0.5	2	1	-	2	1.5	0.5	1.5	1.5	1.5	0	1.5
Anal to caudal plates [ps]	11	9	-	11	10.5	0.5	11	11			11	0	11	11	-	12	11.5	0.5	10	10	-	11	11	0.5	11	11	11	0	11
Branched dorsal-fin rays [D]	7	6	-	8	7	1	7	7			7	0	7	7			7	0	7	7			7	0	7	7	7	0	7
Branched anal-fin rays [A]	4	4	-	5	4	1	5	5			5	0	5	4	-	5	5	1	5	5	-	5	5	0	5	5	5	0	5

*Guyanancistrus* sp. « big mouth »: Mol et al., 2007 [56]: 112 (potentially new species; collection localities); Wan Tong You, 2007 [108]: 249 (behavior in aquarium)

*Guyanancistrus* sp. « Bigmouth »: Mol, 2012 [55]: 450–451 (short description, distribution and illustration)

*Pseudancistrus* sp. Nassau: Covain & Fisch-Muller, 2012 [50]: 233 (molecular phylogeny of *Pseudancistrus sensu lato*)

*Guyanancistrus* sp. Nassau: Covain & Fisch-Muller, 2012 [50]: 244 (generic placement); Mol et al., 2012 [45]: 274 (distribution in Suriname), 286 (threatened species)

**Holotype.** MHNG 2679.100, 42.0 mm SL; Suriname: Sipaliwini: Paramaka Creek (Na3 site), Marowijne River Drainage, Nassau Mountains (4°51’36” N 54°35’ 30” W); J. H. Mol et al., RAP expedition, 3 April 2006.

**Paratypes.** All from Suriname: Sipaliwini, Nassau Mountains, Paramaka Creek Basin, Marowijne River Drainage. MHNG 2745.064 (ex MHNG 2679.100), 2, 28.9–42.8 mm SL; collected with the holotype. MHNG 2679.099 (MUS 299–302), 4, 20.3–34.7 mm SL (1 measured, 34.7 mm SL); MHNG 2690.022, 1 postlarve; Paramaka Creek; J. H. Mol et al., RAP expedition, 29 March– 4 April 2006. AUM 50388, 14 (8 measured, 22.9–49.7 mm SL); NZCS F 7095 (ex AUM 50388), 1; NZCS F 7096 (ex AUM 50388), 1, IJs Creek, headwater tributary of Paramaka Creek (4°49‘14” N 54°36’19” W); J. W. Armbruster et al., 9 Sept. 2009. AUM 50396, 16 (5 measured, 38.8–49.6 mm SL); NZCS F 7097 (ex AUM 50396), 1; NZCS F 7098 (ex AUM 50396), 1, unnamed tributary of IJs Creek (4°51’04” N 54°35’24” W); J. W. Armbruster et al., 12 Sept. 2009. AUM 50740, 6 (3 measured, 37.2–47.0 mm SL); Creek entering Paramaka Creek below the mouth of IJs Creek; J. W. Armbruster & J. L. Wiley, 18 March 2010. AUM 50737, 4 (2 measured, 35.7–50.1 mm SL); Paramaka Creek (4°51’22” N 54°35’01” W); J. W. Armbruster & J. L. Wiley, 18 March 2010. AUM 50763, 2 (1 measured, 61.0 mm SL); Paramaka Creek just downstream of mouth of IJs Creek (4°51’39” N 54°353’59” W); J. W. Armbruster & J. L. Wiley, 19 March 2010.

**Diagnosis.** *Guyanancistrus nassauensis* is distinguished from all congeners except *G*. *brevispinis* by its specific barcode sequences (GBOL093-13 and GBOL732-14). It is morphologically discriminated from all congeners by a small adult size (largest specimen observed 61 mm SL; adult size likely reached around 40 mm SL), by a reduced number of anal-fin rays (4 branched rays *vs* 5, apart from exceptional specimens), and by a wide oval mouth with both large dentary and premaxillary tooth cups (in % of head length, respectively: 24.2–31.9, mean 27.6, *vs* 23.6 or less except in *G*. *niger*, and 25.4–31.4, mean 28.1, *vs* 24.5 or less). Only *Guyanancistrus niger* has dentaries nearly as large (22.5–26.3, mean 25.0% of HL) but its premaxillaries are shorter (21.7–23.6, mean 22.6% of HL).

*Guyanancistrus nassauensis* is distinguished from *G*. *longispinis* and from *G*. *niger* by a much shorter pectoral-fin spine (in % of SL: 22.2–26.3, mean 24.4, *vs* 31.9–45.5, mean 40.2, and 33.3–48.0, mean 42.8, respectively), and by color pattern (body and fins uniformly brown or with indistinct medium sized paler spots, *vs* brown-black with either small roundish yellow spots for *G*. *longispinis*, or white dots for *G*. *niger*). It is further separated from all *G*. *brevispinis* group species by having, on average, the widest body, the deepest and longest head, the largest interbranchial distance, the shortest fins, and the highest number of teeth (see Table 5).

**Description.** Morphometric and meristic data in Table 5. Small-sized species (largest specimen observed 61.0 mm SL; holotype, 42.0 mm SL, likely a breeding male). Head and body dorsoventrally depressed and wide. Dorsal profile gently convex from snout tip to dorsal-fin origin, usually more flattened posterior to orbit, slightly convex and sloped ventrally from dorsal-fin origin to adipose fin, then slightly concave to procurrent caudal-fin rays, and rising to caudal fin. Ventral profile flat from snout to base of caudal fin.

A low median ridge from tip of snout to nostrils, sometimes bordered by lateral depression, a slight elevation anterior to orbits, supraoccipital slightly convex to flat. Dorsal margin gently flattened from base of first branched dorsal-fin ray to base of adipose fin between very slight ridges formed with lateral plates of dorsal series. First lateral plates of mid-ventral series forming low lateral ridge. Caudal peduncle roughly ovoid in cross section, flattened ventrally, and more compressed posteriorly.

Large, rounded and laterally flattened snout. Eye relatively small. Large oval mouth, lower lip wide, not or just reaching pectoral girdle, upper lip narrower. Lips forming an oval disk, covered with short papillae. Presence of a single narrow buccal papilla. Very short maxillary barbel. Teeth short and strong with a relatively long bicuspid crown, lateral lobe about half size of medial lobe.

Head and body plated dorsally, plates generally covered by short and uniformely distributed odontodes.Tip of snout largely naked. Lateral margin of snout covered with plates forming a rigid armor with short odontodes. Opercle supporting odontodes. A narrow unplated area bordering posterodorsal margin of opercle. Evertible cheek plates with enlarged odontodes in highly variable number, from fewer than ten up to approximately 35 in some large specimens. These cheek odontodes straight with tips curved, the longest usually reaching middle of the opercle, or beyond in large specimens. Two to four rows of plates between supraoccipital plate and dorsal-fin spinelet, nuchal plate often covered by skin. Five series of lateral plates extending to caudal fin. Odontodes on lateral series of plates not forming keels. Odontodes on posterior part of pectoral-fin spine enlarged, only slightly in small specimens, much more significantly in large specimens (presumably males). Abdominal region totally naked. No platelike structure before the anal fin. Ventral part of caudal peduncle covered with plates showing a highly reduced number of odontodes.

Dorsal-fin origin slightly anterior to pelvic-fin origin. Dorsal fin short; when adpressed, far from reaching preadipose unpaired plate. Adipose fin roughly triangular, preceded by one, or two fused into one, median unpaired raised plate. Adipose spine straight or slightly convex dorsally, membrane posteriorly convex. Pectoral-spine short, tip usually reaching the first quarter of pelvic spine, exceptionally extending up to the first third in large specimens (presumably males). Anal fin short with weak spine, its margin convex. Caudal fin slightly concave, ventral lobe longer than dorsal lobe. Fin-ray formulae: dorsal II,7; pectoral I,6; pelvic i,5; anal i,4 (except 1 specimen, 49.6 mm SL, with i,5); caudal i,14, i.

**Coloration.** In alcohol, dorsal part of body uniformly grey-brown, ventral part yellowish except usually patches of melanophores on lateral parts and in the anal region, and abdomen whitish. Fin-rays brownish, with medium-sized spots by some specimens, these spots forming or not forming bands; margin of caudal fin often orange- or red-brown; fin-membranes usually not pigmented, or pigment restricted to areas bordering rays. In life (based on a photograph of one specimen), dorsal coloration of body brown with some lighter ill-defined orange-brown spots; fins orange-brown, fin-membranes hardly pigmented (Fig 7E).

**Distribution and habitat.** *Guyanancistrus nassauensis* is known solely from Paramaka Creek and some of its tributaries, Marowijne River Basin, in the Surinamese Nassau Mountains (an area of approximately 20x20 km2) (Fig 4). At an elevation of 277 m, the type locality is located in a northern branch of Paramaka Creek, a medium-sized and shallow stream (3–7 m. width; less that 50 cm depth) with pools and some riffle habitat, a rocky substrate, and bordered by terra firme rainforest. Water was transparent, with a mean pH of 6.26, conductivity 24.2 μS/cm and temperature 23.2°C. Specimens were collected there by electrofishing with set seine, along with several *Harttiella crassicauda*, another species endemic to streams in the Nassau Mountains.

**Etymology.** The name *nassauensis* is a reference to the distribution of the new species which is only known in streams in the Nassau Mountains, an area now under threat of a proposed bauxite mine and illegal gold mining.

#### *Guyanancistrus brownsbergensis* Mol, Fisch-Muller & Covain, new species

**Fig 13:**
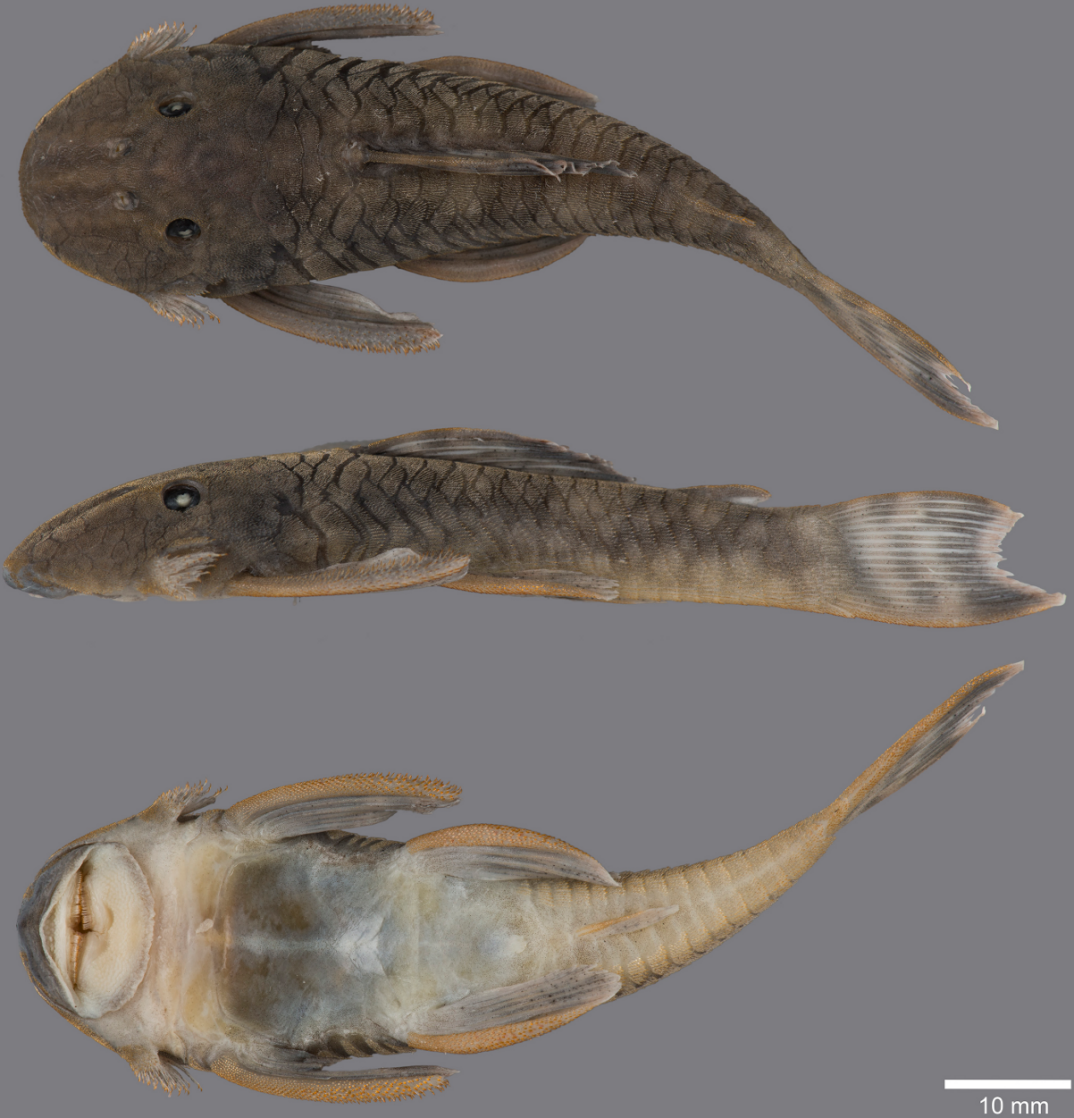
*Guyanancistrus brownsbergensis*. MHNG 2745.065 (JM14 01), holotype; Suriname: 63.8 mm SL; Suriname: Brokopondo: Kumbu Creek, Saramacca River Basin.

urn:lsid:zoobank.org:act: 02C600BB-5C91-4E0E-B25C-86738918BE28

(Figs 6F and 13; Table 5)

**Holotype.** MHNG 2745.065 (JM14 01), 63.8 mm SL; Suriname: Brokopondo: Kumbu Creek above Kumbu Falls, Saramacca River Basin, Brownsberg Nature Park, Brownsberg Mountains (4°56’57” N 55°11’07” W); K. Wan Tong You, 15–16 Feb. 2014.

**Paratypes.** NZCS F 7093 (JM14 02), 55.4 mm SL; MHNG 2745.066 (JM14 03), 49.2 mm SL; collected with the holotype. NZCS F 7094 (SU01-291), 1; MHNG 2723.037 (SU01-280), 1; MHNG 2724–008 (SU01-285), 1; MHNG 2724.009 (SU01-286), 1; MHNG 2724.011 (SU01-296): K. Wan Tong You, 01 July 2011 (all juveniles, 16.6–23.8 mm SL; not measured).

**Diagnosis.** *Guyanancistrus brownsbergensis* is differentiated from all congeners by its specific barcode sequences (GBOL697-14, GBOL689-14, GBOL690-14, GBOL691-14, GBOL726-14, GBOL725-14, and GBOL724-14). It is morphologically distinguished from *G*. *longispinis* and *G*. *niger* by a much shorter pectoral-fin spine (in % of SL: 27.7–29.7, mean 28.7, in SL *vs* 31.9–45.5, mean 40.2, and 33.3–48.0, mean 42.8, respectively), having shorter odontodes, and by color pattern (body and fins medium grey-brown with yellowish beige to light brown medium to large-sized spots, *vs* brown-black with either small roundish yellow spots for *G*. *longispinis*, or white dots for *G*. *niger*). *Guyanancistrus brownsbergensis* is distinguished from all species of the *Guyanancistrus brevispinis* group except *G*. *nassauensis* by a deeper caudal peduncle (11.4–11.6, mean 11.5, *vs* 11.3 or less % of SL). It is differentiated from *G*. *nassauensis* by smaller dentary and premaxillary tooth cusps (18.5 and 17.6% of head length, *vs* 24.2–31.9, mean 27.6, and 25.4–31.4, mean 28.1) by an anal fin with 5 branched rays (*vs* 4).

The caudal peduncle of *G*. *brownsbergensis* is not only deep, but it is also short compared to *G*. *tenuis* and *G*. *megastictus* (28.0–29.1, mean 28.5 in % of SL *vs* respectively 29.4–32.1, mean 31.1, and 31.0–31.6, mean 31.3). *Guyanancistrus brownsbergensis* can further be distinguished from *G*. *brevispinis* by longer evertible cheek odontodes (reaching beyond posterior end of opercle, *vs* not reaching its last quarter), and by a larger number of plates between adpressed dorsal fin and adipose fin (3–3.5, mean 3, *vs* 0.5–3, mean 2). It is separated from *G*. *teretirostris* and *G*. *megastictus* by a longer pelvic-fin spine (reaching beyond end of anal-fin base *vs*, respectively, not reaching origin of anal fin and not reaching beyond end of anal-fin base).

**Description.** Morphometric and meristic data in Table 5. Head and body strongly dorsoventrally depressed. Dorsal profile gently convex from snout tip to dorsal-fin origin, flattened posterior to orbit, slightly convex and sloped ventrally from dorsal-fin origin to end of adipose fin, then slightly concave and rising to caudal fin. Ventral profile flat from snout to base of caudal fin.

Very low median ridge from tip of snout to nostrils present, parallel to this, similar inconspicuous ridges from snout border to nostrils, then somewhat more elevated to orbits, supraoccipital nearly flat. Dorsal margin gently flattened along dorsal-fin base between very slight ridges formed with lateral plates of dorsal series. First lateral plates of mid-ventral series forming low lateral ridge. Caudal peduncle high, roughly ovoid in cross section, flattened ventrally, and more compressed posteriorly.

Snout rounded anteriorly. Eye relatively small. Lips forming an oval disk, covered with short papillae. Presence of a single small and narrow buccal papilla. Lower lip wide, not reaching pectoral girdle, upper lip narrower. Very short maxillary barbel. Teeth slender, bicuspid, lateral lobe about half size of medial lobe.

Head and body plated dorsally, plates generally covered by short and uniformely distributed odontodes. Tip of snout naked; small (small specimens) to minute (holotype, which is the largest specimen) naked area on each side of the latter; dorsolateral margin of the upper lip supporting patches of odontodes (very small in the smallest specimen). Lateral margin of snout covered with plates forming a rigid armor with short odontodes. Opercle supporting odontodes. A narrow unplated area bordering posterodorsal margin of opercle. Evertible cheek plates with approximately 25 to 50 enlarged odontodes. These cheek odontodes straight with tips curved, the longest reaching beyond end of opercle. Three rows of plates and a curved nuchal plate between supraoccipital plate and dorsal-fin spinelet. Five series of lateral plates extending to caudal fin. Odontodes on lateral series of plates not forming keels. Odontodes on posterior part of pectoral-fin spine moderately enlarged. Abdominal region totally naked. No platelike structure before the anal fin. Ventral part of caudal peduncle plated; presence of a large smooth area devoid of odontodes around anal fin.

Dorsal-fin origin slightly anterior to pelvic-fin origin. Dorsal fin relatively short; when adpressed, distant by one (holotype) or two plates from median unpaired plate preceding adipose fin. Adipose fin roughly triangular; spine slightly convex dorsally, membrane straight or slightly concave posteriorly. Pectoral-spine tip reaching first quarter of pelvic spine. Anal fin with weak spine, its margin convex. Caudal fin concave, ventral lobe longer than dorsal lobe. Fin-ray formulae: dorsal II,7; pectoral I,6; pelvic i,5; anal i,5; caudal i,14, i.

**Coloration.** Dorsal coloration pattern grey-brown in life, lighter in alcohol. Medium to large-sized spots irregularly distributed on dorsal part of body. These spots are yellowish-beige to light brown. Spots on fins, at least on caudal, forming bands. Border of dorsal and caudal fins orangish colored in life. Ventral coloration pale yellow and unspotted (Fig 7F).

**Distribution and habitat.** Collected only in the Upper Kumbu Creek in the Brownsberg Nature Park, Brownsberg Mountains, at altitude 200–430 m above mean sea level (Fig 4). The Upper Kumbu Creek at Kumbu Falls (430 m asl) is a small mountain stream (2.5–3.7 m wide, 28–50 cm water depth) with cool (23.1–23.2°C) water, high dissolved oxygen content (93–96% saturation; 7.08–7.72 mg/L), a pH of 7.0–7.5, conductivity 30.8–31.6 μS/cm, and a current strength of 0.29–0.56 m/s (12 July 2014). The bottom substrate consists of sand, gravel, pebbles, boulders and bedrock. The water is mostly clear. Overhanging vegetation, leaf litter and some woody debris offer shelter.

**Etymology.** Species named for the Brownsberg Nature Park in Brownsberg Mountains, in which it was found, and which is presently under threat from illegal gold mining [109].

#### *Guyanancistrus teretirostris*, new species

urn:lsid:zoobank.org:act:3D9F8677-4505-47EE-8111-7636ABF48A25

(Fig 14; Table 5)

**Fig 14:**
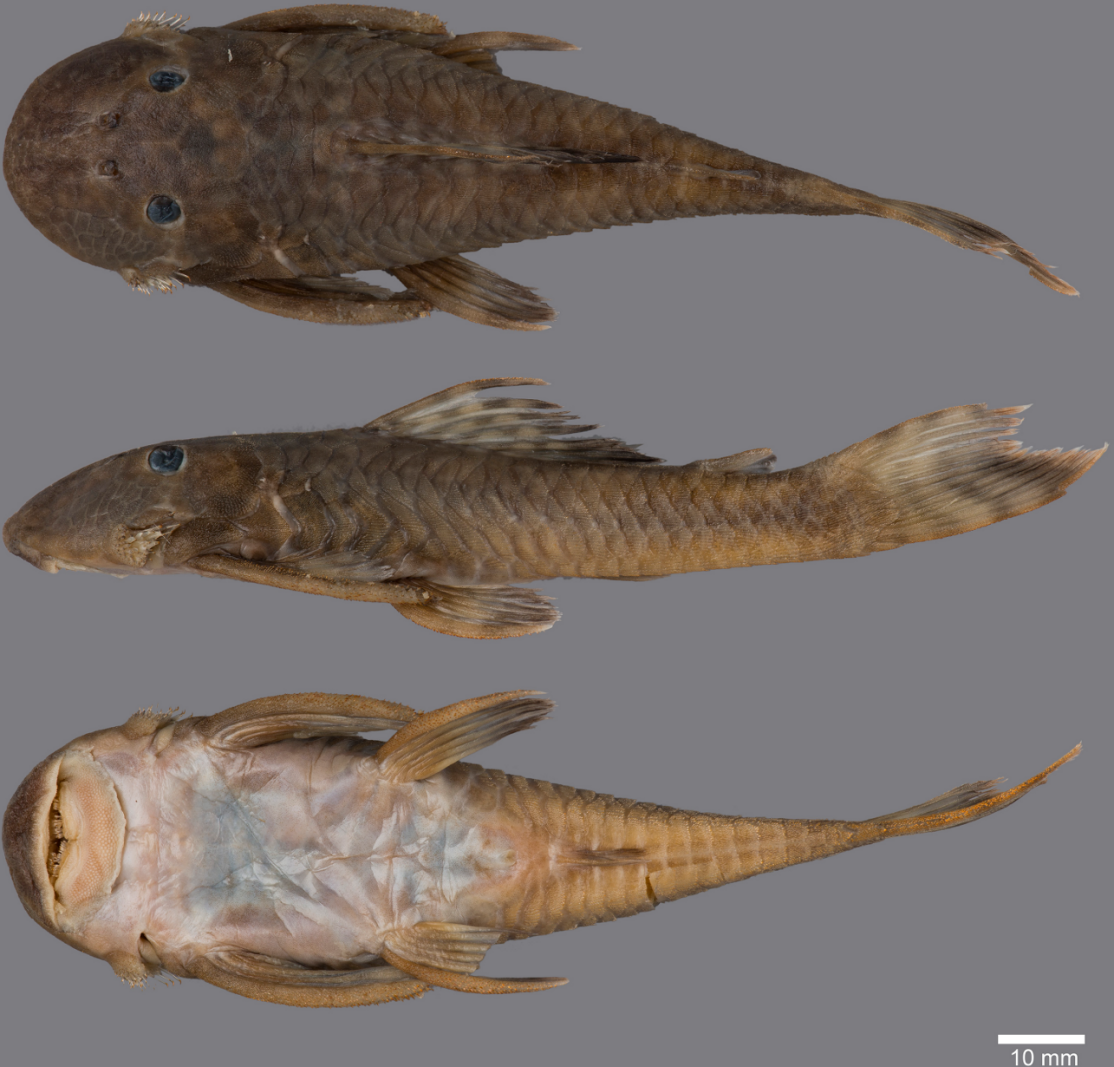
*Guyanancistrus teretirostris*. MZUSP 117149, holotype, 97.6 mm SL; Brazil: Sipaliwini/Parú Savannah in Trio Amerindian territory at the Suriname-Brazil border, tributary of Parú de Oeste River.

**Holotype.** MZUSP 117149 (ex MHNG 2723.004; SU07-654), 97.6 mm SL; Brazil: Sipaliwini-Parú Savannah in Trio Amerindian territory at the Suriname-Brazil border, Vier Gebroeders (Four Brothers) Mountains in a tributary of the Parú de Oeste River, gift of the Trio tribe in Sipaliwini, 20–21 Oct. 2007.

**Paratypes.** MHNG 2723.004 (SU07-652, 653), 2, 87.0 and 97.2 mm SL; same data as holotype.

**Diagnosis.** *Guyanancistrus teretirostris* is distinguished from all congeners by its specific barcode sequences (GBOL735-14, GBOL734-14, and GBOL733-14). It is morphologically distinguished from *G*. *longispinis* and from *G*. *niger* by a much shorter pectoral-fin spine (in % of SL: 27.4–29.7, mean 28.5, *vs* respectively 31.9–45.5, mean 40.2, and 33.3–48.0, mean 42.8, mean 44.5) supporting shorter odontodes, and by color pattern (yellow-beige to light brown small to medium-sized spots on body and fins, *vs* either small roundish yellow spots for *G*. *longispinis*, or white dots for *G*. *niger*). In the *brevispinis* group, *Guyancistrus teretirostris* is distinguished from *G*. *nassauensis*, *G*. *brownsbergensis*, and *G*. *megastictus* by a narrower body (cleithral width in % of SL 29.7–31.1, mean 30.5, *vs* respectively: 32.2–36.6, mean 34.3; 31.5–31.7, mean 31.6; and 31.8–32.7, mean 32.2), and, from the latter three species, by shorter dorsal- and pelvic-fin spines (dorsal spine in % of SL: 23.0–23.5, mean 23.3, *vs* respectively: 24.3–25.6, mean 25.0; 26.0; and 24.5–26.1, mean 25.3; pelvic spine in % of SL: 21.5–23.5, mean 22.5, *vs* respectively: 25.0–26.7, mean 26.0; 25.3; 24.6–26.3, mean 25.5). Pelvic-fin length discriminates *teretirostris* from *G*. *tenuis* (23.5–26.1, mean 24.8), from which it can futher be distinguished by caudal peduncle depth (10.5–10.8, mean 10.6% of SL, *vs* 8.9–9.6, mean 9.3), and by mean number of plates bordering supraoccipital (3–4, mean 3.5, *vs* 3–5, mean 4.5), and separating adpressed dorsal fin and adipose fin (2–3, mean 2.5, *vs* 3–4, mean 3). *Guyanancistrus teretirostris* is distinguished from *G*. *brevispinis* by longer evertible cheek odontodes (reaching end of opercle or beyond *vs* reaching first to third quarter).

A particularly short but also depressed head further discriminates G. *teretirostris* from G. *nassauensis* (in % of SL, head length: 31.7–32.3, mean 31.9, *vs* 32.2–40.7, mean 36.4; head depth: 13.8–15.3, mean 14.7, *vs* 16.0–18.1, mean 17.1). On average, head length distinguishes it from all other species of the *brevispinis* group, including *G*. *brevispinis* (see Table 5).

**Description.** Morphometric and meristic data in Table 5. Head and body dorsoventrally depressed. Dorsal profile gently convex from snout tip to orbit level, then nearly flat, slightly convex and sloped ventrally from dorsal-fin origin to adipose fin, then slightly concave to procurrent caudal-fin rays, and rising to caudal fin. Ventral profile flat from snout to base of caudal fin.

Dorsal contour of head smooth, no ridge or keel, inconspicuous rounded elevations on the midline of the snout and anterior to orbits, supraoccipital nearly flat. Dorsal margin gently flattened from base of first branched dorsal-fin ray to base of adipose fin between very slight ridges formed with lateral plates of dorsal series. First lateral plates of mid-ventral series forming low lateral ridge. Caudal peduncle roughly ovoid in cross section, flattened ventrally, and more compressed posteriorly.

Snout fully rounded anteriorly. Eye moderately large. Lips forming an oval disk, covered with short papillae. Presence of a single narrow buccal papilla. Lower lip wide, not reaching pectoral girdle, upper lip narrower. Short maxillary barbel. Teeth bicuspid, lateral lobe about half size of medial lobe.

Head and body plated dorsally, plates generally covered by short and uniformely distributed odontodes. Tip of snout naked; very small area on each side of the latter is also naked in the smaller paratypes; dorsolateral margin of the upper lip supporting several platelets with short odontodes, or naked (smallest paratype). Lateral margin of snout covered with plates forming a rigid armor with short odontodes. Opercle supporting odontodes. A narrow unplated area bordering posterodorsal margin of opercle. Evertible cheek plates with approximaterly 25–35 enlarged odontodes. These cheek odontodes straight with tips curved, longest nearly reaching posterior end of opercle (smallest paratype) or beyond (holotype and second paratype). Three rows of plates and a curved nuchal plate between supraoccipital plate and dorsal-fin spinelet. Five series of lateral plates extending to caudal fin. Odontodes on lateral series of plates not forming keels. Odontodes on posterior part of pectoral-fin spine only slightly enlarged. Abdomen totally naked. No platelike structure before the anal fin. Ventral part of caudal peduncle plated; presence of a moderately large smooth area devoid of odontodes around anal fin.

Dorsal-fin origin slightly anterior to pelvic-fin origin. Dorsal fin relatively short; when adpressed, distant by at least one plate from median unpaired plate preceding adipose fin. Adipose fin roughly triangular; spine slightly convex dorsally, membrane posteriorly convex. Pectoral-spine tip nearly reaching third of pelvic spine (holotype), or less. Anal fin with weak spine, its margin convex. Caudal fin concave, ventral lobe longer than dorsal lobe. Fin-ray formulae: dorsal II,7; pectoral I,6; pelvic i,5; anal i,5 (i,4 in paratype 87 mm SL); caudal i,14,I.

**Coloration.** In alcohol, dorsal ground color of body medium grey-brown, somewhat darker on head and lighter on lower part of caudal peduncle. Body dorsally covered with yellow-beige to light-brown small to medium-sized spots, usually rounded anteriorly and more irregular in shape posteriorly. Body ventrally yellow-beige, with abdomen whitish, unspotted.

All fins of similar color to body, and spotted, anal fin excepted. Spots of dorsal-fin medium sized and rounded. Spots of paired fins similar but less distinct; anterior part of these fins clearly darker that posterior part. Spots of caudal fin forming three to four large light and irregular transverse bands; margin of fin apparently orangish colored.

**Distribution.** Known from the Upper Parú de Oeste River (Fig 4).

**Etymology.** The species name *teretirostris*, is derived from the Latin words *teres*, meaning rounded and smooth, and *rostris*, meaning snout; an allusion to the snout shape of this species.

#### *Guyanancistrus tenuis*, new species

**Fig 15:**
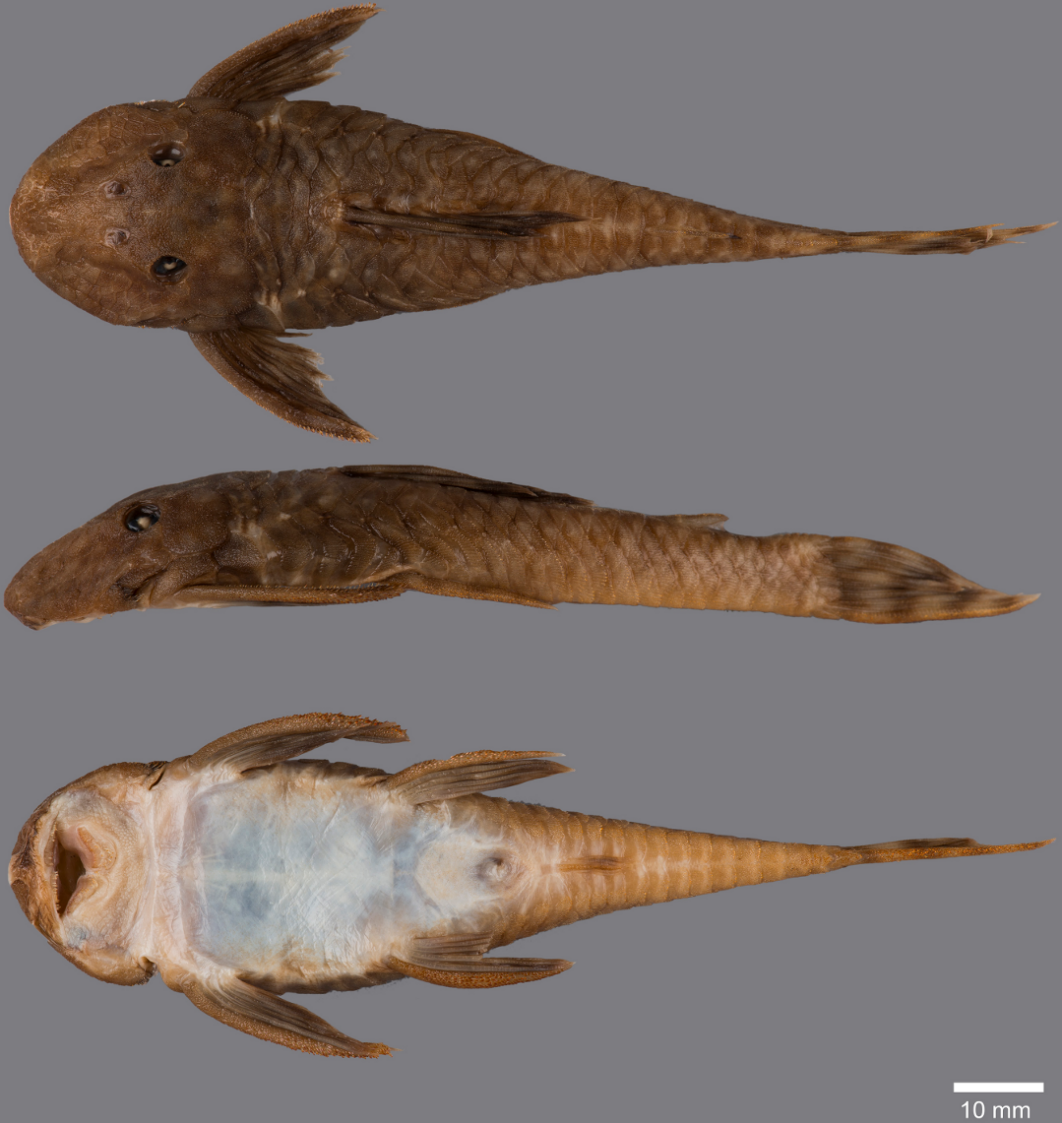
*Guyanancistrus tenuis*. MZUSP 117148, holotype, 90.9 mm SL; Brazil: Para: small tributary of Rio Mapaoni.

**Fig 16:**
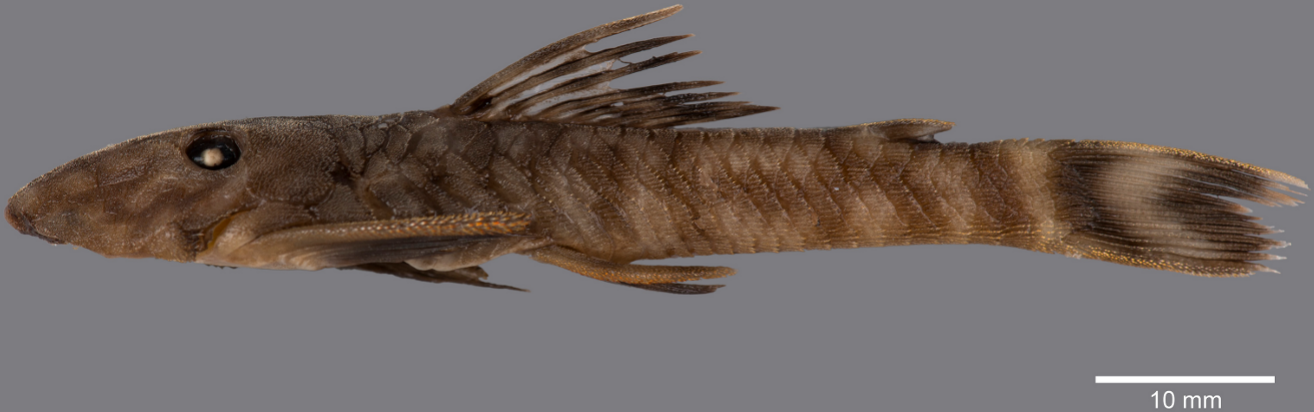
*Guyanancistrus tenuis*. MNHN 2002–3537, paratype, 57.4 mm SL.

urn:lsid:zoobank.org:act:225451B6-1C40-4DC0-BC5A-553A4B2530D4

(Figs 15 and 16; Table 5)

**Holotype.** MZUSP 117148 (ex MNHN 2002–3537; GF Mit06), 90.9 mm SL; Brazil: Para: small tributary of Rio Mapaoni, upper Jari River Basin, Massif du Mitaraka (2°16’45”N 54°32’39”W); P. Keith & P. Gaucher, 25 Oct. 2002.

**Paratypes.** MNHN 2002–3537, 19, 23.7–81.1 mm SL; MHNG 2745.067, 12, 25.0–89.8 mm SL (GF Mit01-05); same data as holotype.

**Diagnosis.** *Guyanancistrus tenuis* is distinguished from all congeners by its specific barcode sequences (GBOL739-14, GBOL738-14, and GBOL737-14). Morphologically, it is distinguished from *G*. *longispinis* and *G*. *niger* by a much shorter pectoral-fin spine (in % of SL: 26.0–28.1, mean 27.0, *vs* respectively 31.9–45.5, mean 40.2, and 33.3–48.0, mean 42.8) supporting shorter odontodes, and by color pattern (yellow-beige medium to large-sized spots and bands on body and fins, *vs* small roundish yellow spots for *G*. *longispinis*, or white dots for *G*. *niger*). *Guyanancistrus tenuis* is a particularly slender species, and, with *G*. *megastictus*, it is the most depressed of all *brevispinis* group species. *Guyanancistrus tenuis* can be separated from *G*. *nassauensis* and *G*. *brownsbergensis* by lower head depth values (13.5–14.8 mean 14.0% of SL *vs* 15.2 or more), and from all species but *G*. *brevispinis* by a lower caudal peduncle depth (8.9–9.6 mean 9.3% of SL *vs* 10.4 or more), but only mean head depth discriminates *G*. *tenuis* from *G*. *brevispinis* (14.1–19.6, mean 16.2% of SL in the latter). In the *brevispinis* species group, *G*. *tenuis* additionally shows the narrowest body, distinguishing it from *G*. *nassauensis* and *G*. *megastictus* (cleithral width in % of SL: 27.9–31.7, mean 30.1, *vs* respectively: 32.2–36.6, mean 34.3; and 31.8–32.7, mean 32.2). *Guyanancistrus tenuis* is distinguished from *G*. *brevispinis* by longer evertible cheek odontodes (reaching last quarter of opercle up to largely beyond end of opercle in specimens of approximately 60 mm SL *vs* reaching first to third quarter of it).

*Guyanancistrus tenuis* can further be distinguished from *G*. *brevispinis* by a higher number of plates bordering the supraoccipital (3–5, mean 4.5, *vs* 2–3, mean 3) and between the adpressed dorsal fin and the adipose fin (3–4, mean 3, *vs* respectively 0.5–3, mean 2), from *G*. *nassauensis* by smaller dentary and premaxillary tooth cusps (in % of head length, respectively 15.8–20.6, mean 17.4, *vs* 24.2–31.9, mean 27.6, and 15.1–21.1, mean 18.0, *vs* 25.4–31.4, mean 28.1) and by an anal fin with 5 branched rays (*vs* 4), and from *teretirostris* by a longer pelvic-fin spine (23.5–26.1, mean 24.8% of SL *vs* 21.5–23.5, mean 22.5).

**Description.** Morphometric and meristic data in Table 5. Head and body up to caudal peduncle very dorsoventrally depressed and narrow, resulting in a slender aspect. Dorsal profile gently convex from snout tip to orbit level, then nearly flat to dorsal-fin origin, slightly convex and sloped ventrally from that point to adipose fin, then slightly concave to procurrent caudal-fin rays, and rising to caudal fin. Ventral profile flat from snout to base of caudal fin.

Dorsal contour of head smooth, usually a very low median ridge from tip of snout to nostrils, slight elevation anterior to orbits, sometimes (including holotype) bordered by a shallow lateral depression, supraoccipital nearly flat. Dorsal margin gently flattened from base of first branched dorsal-fin ray to base of adipose fin between very slight ridges formed with lateral plates of dorsal series. First lateral plates of mid-ventral series forming low lateral ridge. Caudal peduncle roughly ovoid in cross section, flattened ventrally, and more compressed posteriorly.

Snout rounded anteriorly. Eye moderately large. Lips forming an oval disk, covered with short papillae. Presence of a single narrow buccal papilla. Lower lip wide, not reaching pectoral girdle, upper lip narrower. Short maxillary barbel. Teeth slender, bicuspid, lateral lobe about half size of medial lobe.

Head and body plated dorsally, plates generally covered by short and uniformely distributed odontodes. Tip of snout naked, and often (particularly in small specimens) also a very small naked area on each side of the latter, separated by a plated area which continues for a short distance on dorsolateral margin of upper lip. Lateral margin of snout covered with plates forming a rigid armor with short odontodes. Opercle supporting odontodes; on its ventral margin, odontodes usually slightly enlarged. A relatively large unplated area bordering posterodorsal margin of opercle. Evertible cheek plates with enlarged odontodes in highly variable number, from approximately 10 up to approximately 35 in large specimens. These cheek odontodes straight with tips curved, longest not reaching middle of opercle in smallest specimens, but reaching it or beyond in specimens of approximately 50 mm SL, and reaching last quarter to well beyond end of opercle in specimens of approximately 60 mm SL (beyond in holotype). Usually three rows of plates and a curved nuchal plate between supraoccipital plate and dorsal-fin spinelet. Five series of lateral plates extending to caudal fin. Odontodes on lateral series of plates not forming keels. Odontodes on posterior part of pectoral-fin spine very slightly enlarged. Abdominal region totally naked. No platelike structure before the anal fin. Ventral part of caudal peduncle plated; a moderately large smooth area devoid of odontodes around anal fin.

Dorsal-fin origin slightly anterior to pelvic-fin origin. Dorsal fin short; when adpressed, tip of fin very distant from adipose fin, and even far from reaching preadipose unpaired plate. Adipose fin roughly triangular, preceded by one, or two fused into one, median unpaired raised plate. Adipose spine straight or slightly convex dorsally, membrane posteriorly straight or slightly convex. Pectoral-spine tip reaching slightly over pelvic-fin origin. Anal fin with weak spine, its margin convex. Caudal fin concave, ventral lobe longer than dorsal lobe. Fin-ray formulae: dorsal II,7; pectoral I,6; pelvic i,5; anal i,5; caudal i,14, i.

**Coloration.** In alcohol, dorsal ground color of body brown, covered with yellow-beige medium-sized spots on head, becoming gradually much larger spots up to end of caudal peduncle. In small specimens, some spots are roundish and large (but covering fewer than four plates) but spots usually coalesce to form large and highly contrasted stripes on posterior part of body (Fig 16). Ventrally, color of body more or less uniformly light brown apart from the abdomen, which is mainly whitish, sometimes with diffuse brown pigmentation.

All fins colored similarly to dorsum, and spotted. Spots of dorsal fin medium sized, forming transverse bars or not; usually a dark spot on membrane between origin of spine and first branched ray. Spots of other fins less distinct. Spots of caudal fin forming two to three highly constrasted, large and irregular light-colored transverse bands; tips of fin light-colored.

**Distribution.** *Guyanancistrus tenuis* is known solely from a small forest tributary of the Mapaoni River, Upper Jari River Basin, in the Massif du Mitakara, a mountain range in the far southwest of French Guiana (Fig 4). This north-south oriented mountain creek was essentially rocky, shallow (20–60 cm depth), with medium to strong currents, and some pools.

**Etymology.** The name *tenuis* is a Latin word meaning thin, in reference to the slender body of the species.

#### *Guyanancistrus megastictus*, new species

**Fig 17:**
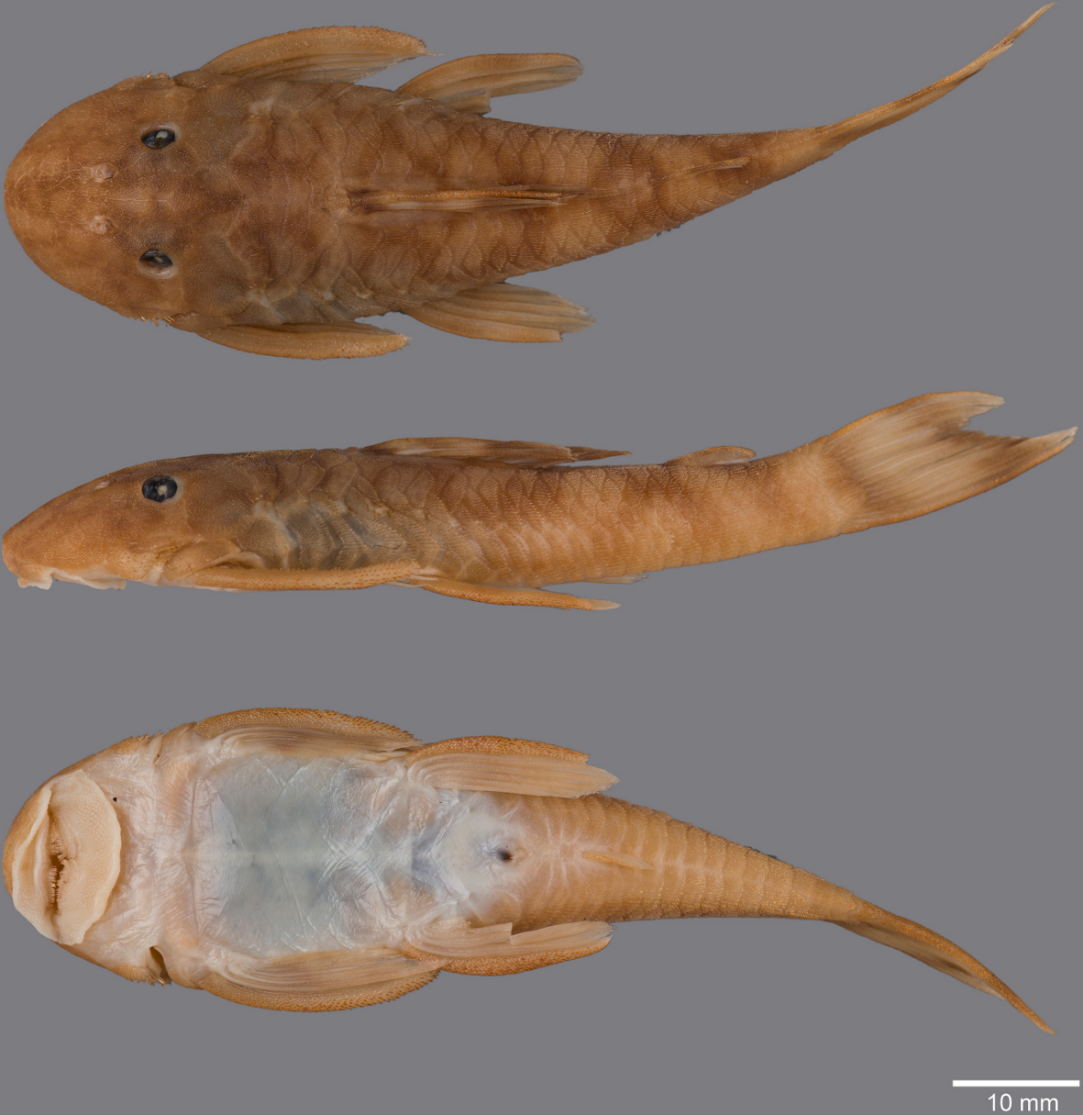
*Guyanancistrus megastictus*. MNHN 2002–3508, holotype, 62.7 mm SL; French Guiana: Crique Alama.

urn:lsid:zoobank.org:act:AAB3CE9E-055D-4DD6-8B9C-682FC8F5E50B

(Figs 6C and 17; Table 5)

**Holotype.** MNHN 2002–3508, 62.7 mm SL; French Guiana: Crique Alama, tributary of Crique Saranou, Maroni River Basin, Massif du Mitaraka (2°18’08”N 54°32’00”W); P. Keith & P. Gaucher, 25 Oct. 2002.

**Paratype.** MHNG 2745.068 (ex MNHN 2002–3508), 1, 57.1 mm SL; same data as holotype.

**Diagnosis.** *Guyanancistrus megastictus* is distinguished from all congeners by specific barcode sequences (GBOL897-15 and GBOL898-15). Morphologically, it is distinguished from *G*. *longispinis* and *G*. *niger* by a shorter pectoral-fin spine (in % of SL: 28.1–28.3, mean 28.2, *vs* respectively 31.9–45.5, mean 40.2, and 33.3–48.0, mean 42.8), supporting shorter odontodes, and by color pattern (pale yellowish medium to very large sized spots or bars on body and fins, *vs* small roundish yellow spots for *G*. *longispinis*, or white dots for *G*. *niger*). Color pattern, with particularly large spots on body posterior to dorsal fin, and a caudal fin mainly light colored by the presence of a single very large yellowish bar (*vs* several light spots or bands), also distinguish *G*. *megastictus* from all *brevispinis* group species.

*Guyanancistrus megastictus* is distinguished: from *G*. *brevispinis* by longer evertible cheek odontodes (reaching last quarter of opercle or beyond its posterior end, *vs* not reaching last quarter of opercle); from *G*. *nassauensis* by smaller dentary and premaxillary tooth cusps (in % of head length, respectively: 16.8, *vs* 24.2–31.9, mean 27.6, and 17.4–17.8, mean 17.6, *vs* 25.4–31.4, mean 28.1) and by an anal fin with 5 branched rays (*vs* 4); from *G*. *brownsbergensis* by less deep head (13.9–15.1, mean 14.5% of SL *vs* 15.2–15.7, mean 15.5) and lower caudal peduncle (10.4–10.9, mean 10.7% of SL *vs* 11.4–11.6, mean 11.5); and from *G*. *teretirostris* and *G*. *tenuis* by a larger body (in % of SL, 31.8–32.7, mean 32.2 *vs* respectively 29.7–31.1, mean 30.5, and 27.9–31.7, mean 30.1).

**Description.** Morphometric and meristic data in Table 5. Dorsal profile gently convex from snout tip to orbit level, then nearly flat to dorsal-fin origin, slightly convex and sloped ventrally from that point to adipose fin, then slightly concave to procurrent caudal-fin rays, and rising to caudal fin. Ventral profile flat from snout to base of caudal fin.

Dorsal contour of head smooth, a very low median ridge from tip of snout to nostrils, slight elevation anterior to orbits, bordered (paratype) or not (holotype) by a shallow lateral depression, supraoccipital nearly flat. Dorsal margin gently flattened from base of first branched dorsal-fin ray to base of adipose fin between very slight ridges formed with lateral plates of dorsal series. First lateral plates of mid-ventral series forming low lateral ridge. Caudal peduncle roughly ovoid in cross section, flattened ventrally, and more compressed posteriorly.

Snout rounded anteriorly. Eye relatively small. Lips forming an oval disk, covered with short papillae. Presence of a single triangular buccal papilla. Lower lip wide, not reaching pectoral girdle, upper lip narrower. Short maxillary barbel. Teeth slender, bicuspid, lateral lobe about half the size of medial lobe.

Head and body plated dorsally, plates generally covered by short and uniformely distributed odontodes. Tip of snout naked, and a minute naked area on each side of the latter, separated by a plated area which continues for a short distance on dorsolateral margin of upper lip. Lateral margin of snout covered with plates forming a rigid armor with short odontodes. Opercle supporting odontodes. A straight unplated area bordering posterodorsal margin of opercle. Evertible cheek plates with approximately 25–30 enlarged odontodes, straight with tips curved, longest nearly reaching posterior end of opercle or beyond. Three rows of plates and a curved nuchal plate between supraoccipital plate and dorsal-fin spinelet. Five series of lateral plates extending to caudal fin. Odontodes on lateral series of plates not forming keels. Odontodes on posterior part of pectoral-fin spine very slightly enlarged. Abdominal region totally naked. No platelike structure before the anal fin. Ventral part of caudal peduncle plated; a moderately large smooth area devoid of odontodes around anal fin.

Dorsal-fin origin slightly anterior to pelvic-fin origin. Dorsal fin short; when adpressed, tip of fin very distant from adipose fin, and even far from reaching preadipose unpaired plate. Adipose fin roughly triangular, preceded by one, or two fused into one, median unpaired raised plate. Adipose spine relatively long compared to other *Guyanancistrus* species, slightly convex dorsally, membrane posteriorly straight. Pectoral-spine tip reaching slightly beyond pelvic-fin origin or nearly one fifth of fin spine (holotype). Anal fin with weak spine, its margin convex. Caudal fin concave, ventral lobe longer than dorsal lobe. Fin-ray formulae: dorsal II,7; pectoral I,6; pelvic i,5; anal i,5; caudal i,14, i.

**Coloration.** In alcohol, dorsal ground color of body a bleached brown, covered with yellow-beige medium-sized spots on head, then large spots, and very large roundish spots (covering at least six lateral plates) and bars posterior to dorsal-fin origin level and up to end of caudal peduncle. Ventrally, color of body more or less uniformly yellowish apart from mainly whitish abdomen.

All fins except anal similarly colored to dorsum, and lightly spotted. Spots of dorsal fin large, forming one or two large transverse bars. Spots on paired fins less distinct. Anal fin yellowish. Caudal fin mainly light colored: narrow brownish base, followed by a very large lightly colored transverse bar, then a narrower brownish transverse bar, and tips of fin light.

In life (Fig 7C), background color greenish-brown, with darker areas surounding the light spots, and caudal-fin base also darker.

**Distribution.** *Guyanancistrus megastictus* is known from a small forest tributary of the Upper Maroni River Basin in the Massif du Mitaraka, a mountain range in the far south-west of French Guiana (Fig 4). The only two known specimens were caught poison fishing in a shallow (20–60 cm depth) and mainly sandy portion of this river named Crique Alama.

**Etymology.** The Latin word *megastictus* is derived from the Ancient Greek *mega*, meaning large, and *stictos*, meaning spotted, in reference to the presence of very large size spots on body and fins.

#### Key to species of *Guyanancistrus*

1 - Presence of distinct spots on body and fins, all spots roundish and smaller than size of a lateral dermal plate; pectoral-fin spine length 31.9–45.5% of SL...........................2
      - Absence of distinct spots on body and fins, or presence of spots at least as large as a dermal plate, or coalescing, or forming bands on posterior part of body and fins; pectoral-fin spine length 22.2–34.4% of SL...............................................3
2 - Body and fins covered with small roundish yellow spots; odontodes on dorsolateral margin of the upper lip minute; dorsal-fin base length 29.0–32.0% of SL............ *G*. *longispinis*
      - Body and fins covered with minute white dots; odontodes on dorsolateral margin of the upper lip elongated (Fig 7D); dorsal-fin base length 24.8–28.8% of SL.........................*G*. *niger*
3 - Anal fin with 4 branched rays; dentary tooth cup 24.2–31.9% of head length *G*. *nassauensis*
      - Anal fin with 5 branched rays; dentary tooth cup 23.6% or less of head length...................................4
4 - Longest evertible cheek odontodes reaching the first half of the opercle (except in some large specimens surpassing 70 mm SL reaching the third quarter but not reaching its last quarter)....................................*G*. *brevispinis*
      - Longest evertible cheek odontodesreaching the last quarter of opercle or beyond its posterior end (except in very small specimens),...........................5
5 - Pelvic-fin spine not reaching origin of anal fin........................*G*. *teretirostris*
      - Pelvic-fin spine reaching beyond origin of anal fin....................................6
6 - Orbital diameter 1.7–2.1 times in interorbital width; depth of caudal peduncle 3.1–3.6 times in its length...............................*G*. *tenuis*
      - Orbital diameter 2.2–2.4 times in interorbital width; depth of caudal peduncle 2.5–3.0 times in its length..............................................7
7 - Pelvic-fin spine reaching beyond end of anal fin base; depth of caudal peduncle 2.5 times in its length................................... *G*. *brownsbergensis*
      - Pelvic-fin spine not reaching beyond end of anal fin base; depth of caudal peduncle 2.9–3.0 times in its length...................................... *G*. *megastictus*

#### *Cryptancistrus* new genus

urn:lsid:zoobank.org:act:F5ADD7D1-7A87-41A2-9DFE-78F04612C4BA

**Type-species**. *Cryptancistrus similis*, new species

urn:lsid:zoobank.org:act:6FE8FA29-E129-4CCA-BA43-27A51DB314E2

**Fig 18:**
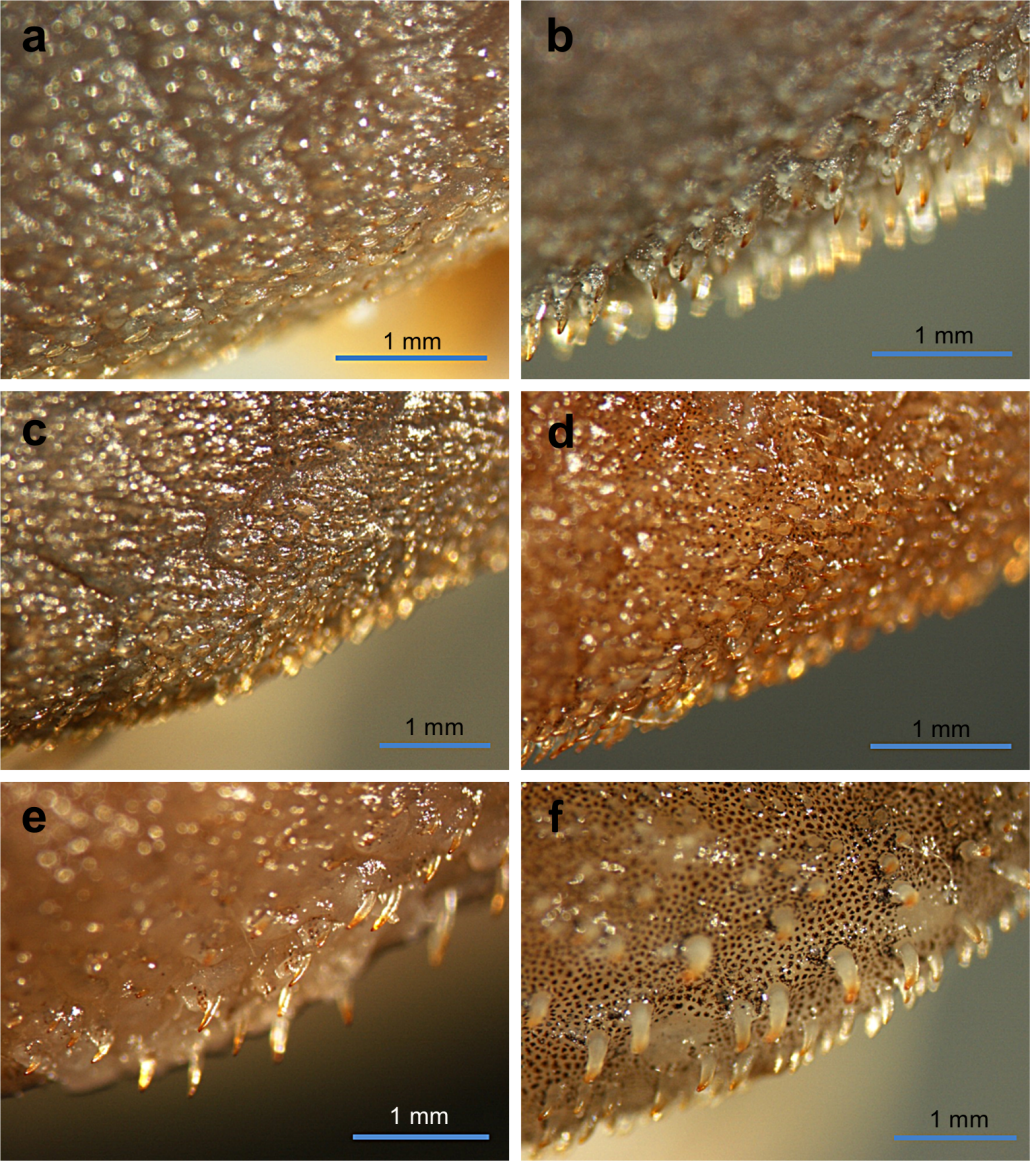
Posterior part of left margin of snout of *Guyanancistus* spp. **a**, *Guyanancistrus brevispinis*, MHNG 2683.029, 63.0 mm SL; **b,** *G*. *nassauensis* AUM 50763, 61.0 mm SL; **c,** *G*. *longispinis*, MHNG 2680.049, 73.3 mm SL; **d,** *G*. *tenuis*, MHNG 2765.067, 64.7 mm SL; **e,** *Cryptancistrus similis*, holotype, MZUSP 117150, 61.7 mm SL; **f,** *Hopliancistrus tricornis*, MHNG 2588.051, 61.6 mm SL.

**Diagnosis.** *Cryptancistrus* is characterized by its unique barcode sequence (GBOL736-14). No unique morphological character was found to diagnose the genus which belongs to the Ancistrini tribe of the Hypstominae subfamily. The following combination of characters distinguishes *Cryptancistrus* from all other Hypostominae genera: head and body dorsoventrally depressed; head and body plates not forming prominent ridge or crest; snout rounded, and covered with contiguous plates except tip region, and posterior part of lateral margin of snout; latter area forming a soft fleshy border, and bearing slightly enlarged odontodes associated with small fleshy tentacules sensu Sabaj et al. [102]); presence of odontodes over a broad area on the opercle; presence of numerous enlarged cheek odontodes supported by evertible plates; these odontodes straight with tips slightly curved, as opposed to strongly hook-shaped; absence of whisker-like cheek odontodes; absence of enlarged odontodes along snout margin; presence of a dorsal iris operculum; lips forming an oval disk; dentary and premaxillary with numerous viliform and bicuspid teeth; presence of a small buccal papilla, no enlarged dentary papilla; seven branched dorsal-fin rays; presence of an adipose fin; no membranous extension between end of dorsal fin and adipose fin; five series of lateral plates extending to caudal fin; lateral plates not keeled and not bearing enlarged odontodes; lateral plates of ventral series on caudal peduncle angular but not keeled; abdominal region entirely naked. *Cryptancistrus* is externally mostly similar to *Guyanancistrus*. It is distinguished from *Guyanancistrus* primarily by the fleshy posterior part of lateral margin of snout bearing slightly enlarged odontodes associated with small fleshy tentacules (*vs* plates along margin of snout forming a rigid armour covered with minute odontodes, absence of tentacules) (Fig 18E). It can additionally be distinguished from *Gruyanancistrus* by a skin region bordering the exposed portion of opercle roughly as large as the latter (*vs* distinctly narrower than the latter).

**Etymology**. The name *Cryptancistrus* is derived from the Greek names *kryptos*, meaning hidden, and *ankistron*, meaning hook, in reference to the genera *Ancistrus*, type genus of the tribe Ancistrini to which it and *Guyanancistrus* Isbrücker, 2001, to which it is externally the most similar, belong.

**Distribution**. Known only from type species locality, Upper Parú de Oeste River basin, Brazil.

#### *Cryptancistrus similis*, new species

**Fig 19:**
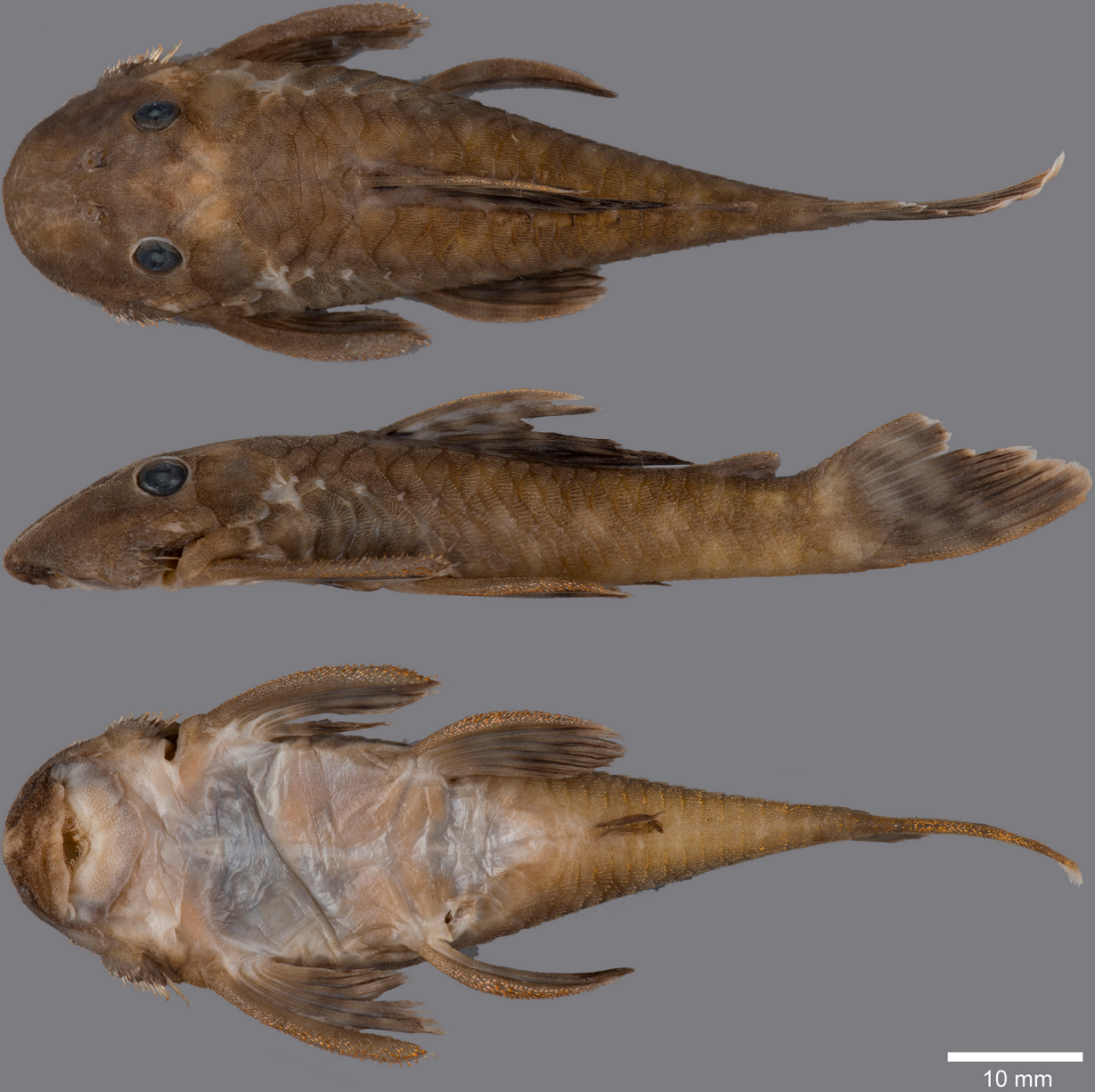
*Cryptancistrus similis*. MZUSP 117150, holotype, 61.7 mm SL; Brazil: Sipaliwini/Parú Savannah in Trio Amerindian territory at the Suriname-Brazil border, tributary of Parú de Oeste River.

(Figs 18 and 19; Table 5)

**Holotype.** MZUSP 117150 (ex MHNG 2723.005; SU07-672), 61.7 mm SL; Brazil: Sipaliwini-Parú Savannah in Trio Amerindian territory at the Suriname-Brazil border, Vier Gebroeders (Four Brothers) Mountains in a tributary of the Parú de Oeste River, gift of the Trio tribe in Sipaliwini, 20–21 Oct. 2007.

**Diagnosis.** As given for genus.

**Description.** Morphometric and meristic data of the holotype (only known specimen) in Table 5. Head and body dorsoventrally depressed. Dorsal profile gently convex from snout tip to orbit level, then nearly flat, slightly convex and sloped ventrally from dorsal-fin origin to adipose fin, then slightly concave to procurrent caudal-fin rays, and rising to caudal fin. Ventral profile flat from snout to base of caudal fin.

Dorsal contour of head smooth, no ridge or keel, inconspicuous rounded elevations on the midline of the snout and anterior to orbits, supraoccipital nearly flat. Dorsal margin gently flattened from base of first branched dorsal-fin ray to base of adipose fin between very slight ridges formed with lateral plates of dorsal series. First lateral plates of mid-ventral series forming low lateral ridge. Caudal peduncle roughly ovoid in cross section, flattened ventrally, and more compressed posteriorly.

Snout rounded anteriorly. Eye relatively large. Lips forming an oval disk, covered with short papillae. Presence of a single narrow buccal papilla. Lower lip wide, not reaching pectoral girdle, upper lip narrower. Very short maxillary barbel. Teeth bicuspid, lateral lobe about half size of medial lobe.

Head and body plated dorsally, plates generally covered by short and uniformly distributed odontodes. Snout plated except tip naked. Anterior margin of snout carrying slightly enlarged odontodes; meeting the latter, dorsolateral margin of upper lip supporting plates and short odontodes. Posterior part of lateral margin of snout forming a soft fleshy border bearing slightly enlarged odontodes with small tentacules sensu Sabaj et al. [102], cutaneous sheath surrounding base of odontodes being enlarged and partially detached from odontodes. Opercle supporting odontodes, those on inferior margin slightly enlarged. A large unplated area bordering posterodorsal margin of opercle. Evertible cheek plates with approximately 40 enlarged odontodes, straight with tips curved, longest reaching beyond the end of opercle (on right side of the holotype; longest odontodes missing on left side). Usually three rows of plates and a curved nuchal plate between supraoccipital plate and dorsal-fin spinelet. Five series of lateral plates extending to caudal fin. Odontodes on lateral series of plates not forming keels. Odontodes on posterior part of pectoral-fin spine enlarged. Abdominal region totally naked. No platelike structure before the anal fin. Ventral part of caudal peduncle plated; presence of a small smooth area devoid of odontodes around anal fin.

Dorsal-fin origin slightly anterior to pelvic-fin origin. Dorsal fin relatively large; when adpressed, nearly reaching adipose fin. Adipose fin roughly triangular, preceded by a median unpaired raised plate. Adipose spine nearly straight, membrane posteriorly convex. Pectoral-spine tip reaching approximately one-fifth of pelvic spine. Anal fin with weak spine, its margin convex. Caudal fin obliquely truncate, very slightly concave, inferior part longer (part of upper lobe damaged by holotype). Fin-ray formulae: dorsal II,7; pectoral I,6; pelvic i,5; anal i,5; caudal i,14, i.

**Coloration.** In alcohol, dorsal ground color of body medium brown, covered with yellowish-beige spots. Spots roundish, medium to large-sized, larger on posterior part than on anterior part, and non-coalescent. Ventrally, plated parts of body yellow-beige, abdomen whitish with some yellow-beige areas.

All fins of slightly darker color than body, and similarly spotted apart from anal. Spots of dorsal fin and paired fins medium sized; a dark spot on membrane between origin of spine and first branched ray. Spots of caudal fin larger, some of them coalescent, more or less forming two large light and irregular transverse bands; margin of fin light.

**Distribution.** Known from a single specimen from the Upper Parú de Oeste River (Fig 4), and collected along with *Guyanancistrus teretirostris* n. sp. and an unidentified *Hypostomus* species, and with two recently described Loricariinae, *Cteniloricaria napova* and *Harttia tuna*.

**Etymology.** The Latin name *similis*, meaning similar, refers to the strong morphological resemblance between the new species of *Cryptancistrus* and the type species of *Guyanancistrus*, *G*. *brevispinis*.

### Biogeography of *Guyanancistrus* members

**Fig 20:**
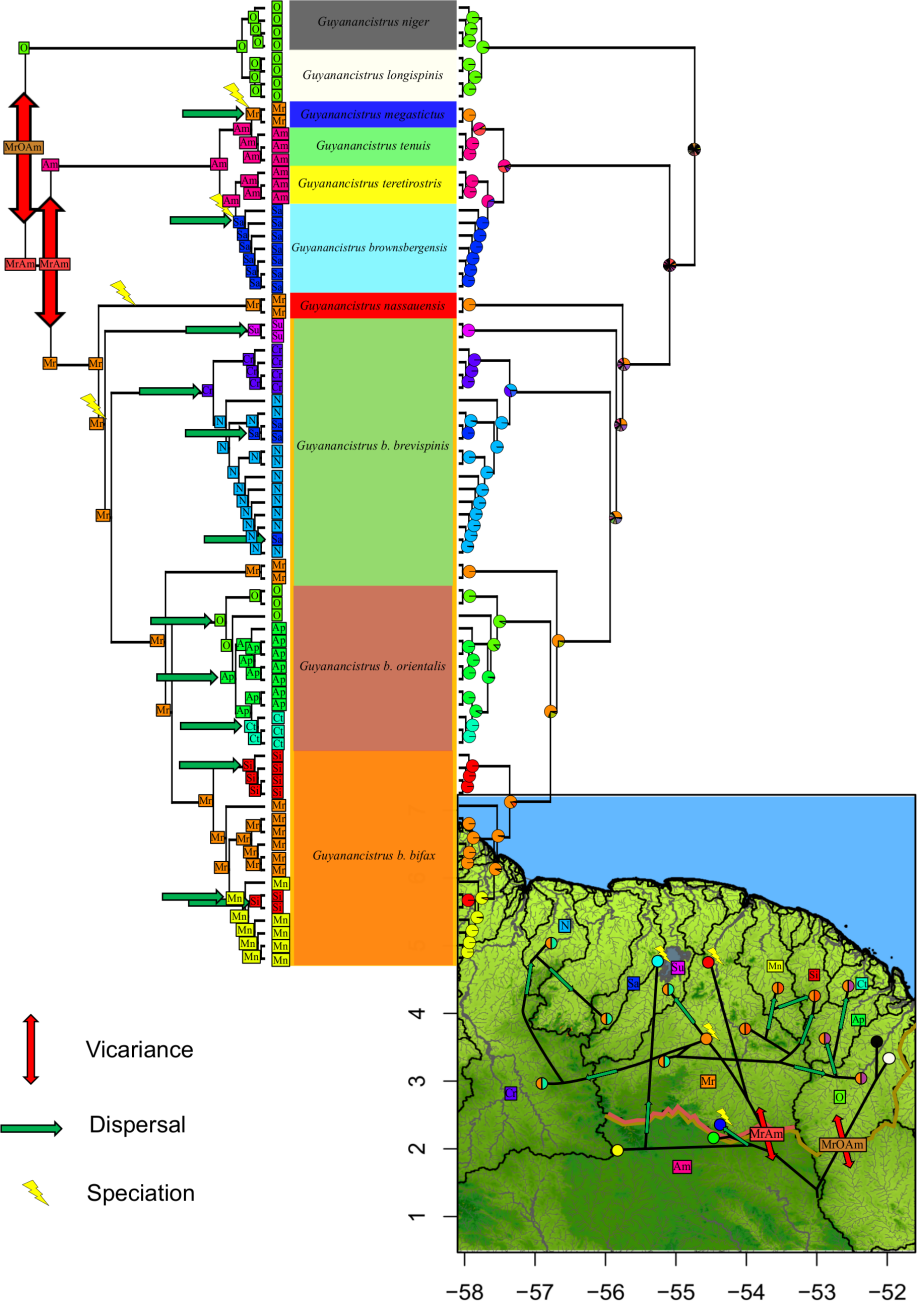
Biogeographical analysis of *Guyanancistrus* spp. using BioGeoBEARS under DEC + *j* model of ancestral area reconstruction. Eleven biogeographical areas corresponding to catchment areas were retained: **Am:** Upper reaches of Amazonian tributaries (including headwaters of Jari and Paru de Oeste rivers); **Cr:** Corantijn River; **N:** Nickerie River; **Sa:** Saramacca River; **Su:** Suriname River; **Mr:** Maroni River; **Mn:** Mana River; **Si:** Sinnamary River; **Ct:** Comté-Orapu River; **Ap:** Approuague River; **O:** Oyapock River. Pie charts at nodes indicate maximum likelihood of ancestral area reconstructions. Vertical double arrow indicates vicariant events (area fragmentation), horizontal simple arrow indicates dispersal events (gain of an area), and lightning identifies speciation events. A map provides interpretation of general displacements of species and populations within Eastern Guianas. Colored chips refer to species and subspecies following color scheme of Fig 4.

Comparison between likelihoods of ancestral area reconstructions along the phylogenetic tree using DEC and DEC + *j* models showed that the latter had significantly better fit (Table 6). Resolutions of the different polytomies of the phylogenetic tree placed the population of *Guyanancistrus brevispinis brevispinis* of Tapanahony River in sister position to *G*. *b*. *bifax* and *G*. *b*. *orientalis* members, and split the population of *G*. *b*. *brevispinis* of Saramacca River in two subpopulations. Even though apparently contradictory to previous results, these resolutions did not impact ancestral area reconstructions, and reinforced the power of the analysis. The biogeographic analysis of *Guyancistrus* members under DEC + *j* model reconstructed a broad ancestral area comprising Amazonian headwaters including Upper Jari and Paru de Oeste rivers, the Oyapock, and Maroni rivers (Fig 20) at the root of the phylogenetic tree, even though this reconstruction was ambiguous (see pie charts of states probabilities in Fig 20). From this ancestral area, the *G*. *niger* and *G*. *longispinis* lineages split from all other ancestral *Guyanancistrus* by vicariance of the Oyapock Basin. Then a second vicariant event occurred between Amazonian headwaters and the Maroni Basin, splitting the *G*. *megastictus*, *G*. *tenuis*, *G*. *teretirostris* and *G*. *brownsbergensis* lineages from that of *G*. *nassauensis* and *G*. *brevispinis*. In the Amazonian group, two dispersals followed by speciation occurred. From an Amazonian ancestor (likely from Jari River), the ancestors of *G*. *megastictus* dispersed toward the Maroni Basin, whereas ancestors of *G*. *brownsbergensis* likely dispersed from the Paru de Oeste River toward the Saramacca River. In the Maroni group, two speciation events occurred, leading on one side to *G*. *nassauensis* and on the other to *G*. *brevispinis*. Dispersal patterns of *G*. *brevispinis* members appeared more complex, with multiple dispersals among Guianese rivers. From the central Maroni River, a first dispersal occurred to the west toward the Suriname River. Then, a second dispersal from headwaters of the Surinamese Maroni (i.e. Marowijne River) took place toward the headwaters of the Corantijn River, whereas part of the ancestral population of the Maroni River stayed in this basin (now present in the Tapanahony River). From the Upper Corantijn River, ancestral populations spread toward the Lower Corantijn, and then dispersed again to the East toward the Nickerie River, and from the latter to the Saramacca River. All these movements lead to the differentiation of the western form *G*. *brevispinis brevispinis*. A second dispersal, again from the Maroni, occurred to the east toward the Oyapock River. From there, ancestral populations successively dispersed to the west toward the Approuague and Comté-Orapu rivers, leading to the establishment of the present eastern form *G*. *brevispinis orientalis*. Finally, once more from the Maroni, ancestral populations dispersed toward the Upper Sinnamary River, and then toward the Mana River, and from the latter toward the Sinnamary leading to the establishment of the present Central form *G*. *brevispinis bifax*.

**Table 6:** Comparision of DEC and DEC+*j* models using Likelihood Ratio Test (LRT). Max nb of areas: maximum number of areas allowed in ancestral geographic range. LnL: log likelihood of the ancestral reconstruction. Numparams: number of parameters included in the model. *d*: rate of range expansion ("dispersal"). *e*: rate of range contraction ("extinction"). *j*: weight of jump dispersal event ("founder effect"). *D* statistic: 2*ΔLnL. DF: number of free parameters.

			Parameter estimates				LRT			
Model	Max nb of areas	LnL	numparams	*d*	*e*	*j*	*D* statistic	*P*-value	test	DF
DEC	4	-91.54	2	5	5	0				
DEC+*j*	4	-78.78	3	3.06680	4.99999	0.00867	25.52	4.40E-07	chi-squared	1

## Discussion

### The genus *Guyanancistrus*

A general similarity of head and body shape, hardly elevated and describing smooth contours, seems to unite all *Guyanancistrus* species. However no shared characteristic was found to be unique for the group. *Guyanancistrus longispinis* and *G*. *niger* appear morphologically quite distinct compared with species of the *brevispinis* group. Even so, mitochondrial and nuclear sequences of the various species unambiguously showed *Guyanancistrus* to be monophyletic ([49, 50, 110], present results), confirming the validity of the genus as it was originally separated from *Lasiancistrus* by its author (Isbrücker in [52]). Nevertheless, while the latter is well diagnosed [111], it is not very closely related to *Guyanancistrus* [50] (see also Armbruster [54]: 12, based on *G*. *brevispinis*). The sister clade of *Guyanancistrus* contained three genera: *Hopliancistrus* confirming results of Covain & Fisch-Muller [50], *Corymbophanes* as in Lujan et al. [49], and the new genus *Cryptancistrus*.

*Hopliancistrus* is globally similar to *Guyanancistrus* but clearly distinguished by the diagnostic presence of up to three strongly hook-shaped cheek odontodes and of enlarged odontodes on the sides of the snout. *Corymbophanes* is also easily distinguished from *Guyanancistrus* by the absence of evertible cheek odontodes, the absence of an adipose fin, and by the presence of an elongate postdorsal ridge of 13–17 raised unpaired platelets [112]. In contrast, *Cryptancistrus* is only distinguished from *Guyanancistrus* by the posterior part of lateral margin of its snout forming a soft fleshy border and bearing slightly enlarged odontodes with small tentacules ([102]; see Fig 18). It is interesting to note that this organisation of odontodes on sides of snout is reminiscent of the condition observed in *Hopliancistrus*, one of its sister genera. These odontodes grow larger in large specimens of *Hopliancistrus* (even becoming stout at the corner of the snout in males), a condition that might be hypothesized for *Cryptancistrus* in the absence of material apart from the single holotype.

Despite the absence of obvious morphological characteristics for *Guyanancistrus*, a unique combination of external characters allows genus recognition with respect to its sister genera, and species assignation within the genus, was found for the diagnosis presented. Armbruster ([54]: 12) suggested that *Guyanancistrus* (which he placed in synonymy of *Pseudancistrus*) was not likely to be a monophyletic entity because of divergence of external characters between species. He particularly cited the development of « at least small hypertrophied odontodes on the snout » of *G*. *niger* (*vs* lack in the others), however these enlarged odontodes are present solely in two lateral tufts on the dorsolateral edges of the upper lip (Fig 7D), not on the plates outlining the snout contour as in the case of *Pseudancistrus*. *Hopliancistrus tricornis* also has two lateral regions of the snout with enlarged odontodes, that suggest similarity with *G*. *niger* [50]; again, however, they are not on the lip, but on the dorsolateral margin of the snout. Odontodes are present to a variable extent on the dorsal margin of the upper lip in several Hypostomines, including other *Guyanancistrus* species (Fig 7A, 7B and 7C) and *Hopliancistrus tricornis*, but they are usually small. Tufts of enlarged odontodes on the dorsolateral edge of the upper lip are thus characteristic of *G*. *niger*. The degree of evertibility of the cheek plates in *G*. *brevispinis* and *G*. *niger* was also found to be divergent by Armbruster (loc. cit.), but we saw no significant difference in this character between *Guyanancistrus* species.

*Chaetostomus megacephalus*, described by Günther in 1868 [113], is an additional but currently insufficiently known species that might also belong to *Guyanancistrus* (see Fig 1). Long considered as an *Hemiancistrus* species, it was moved to *Pseudancistrus* by Armbruster [53, 54], who recently stated that this taxon needs further work [114], but it is evident that it does not correspond to *Pseudancistrus* as defined by Covain & Fisch-Muller [50] (*= Pseudancistrus barbatus* group of de Chambrier & Montoya-Burgos [115]). Indeed, the holotype has evertible cheek odontodes and no enlarged odontodes along the snout margin despite its large size (122.8 mm SL). Morphologically it is similar to *G*. *longispinis* group members, but is quite distinct from the nominal species (Fig 1). The type locality of *C*. *megacephalus* was indicated as “Surinam” in the original description, but Günther [116] later added that it was obtained from the collection of Dr. van Lidth de Jeude specifying that it was “probably from Surinam”. Unfortunately we can only refer to the holotype. Specimens collected in the Essequibo River Basin in British Guiana described by Eigenmann ([117]: 231) as *Hemiancistrus megacephalus* appear more likely a distinct and probably new species [20] morphologically very close to *G*. *longispinis* (Fig 1). Despite extensive field collecting in Suriname, we have been unable to find any additional specimens (as well as other teams in Guyana; J. W. Armbruster, pers. com.).

The case is similar for *Chaetostomus macrops* Lütken, 1874, also known from a single specimen from “*aquis Surinamensibus*”, and considered a synonym of *megacephalus* from the early 20^th^ century [117] until recently [114, 118]. It has a particularly wide and elevated orbital rim reminiscent of that observed in *Hemiancistrus medians*, from which, however, it is easily distinguished by the absence of keeled and rough-toothed trunk plates [20] and by the presence of odontodes over a broad area on the opercle [114]. Morphologically, *C*. *macrops* is most similar to the species collected in the Potaro River by Eigenmann than to *P*. *megacephalus* (see S1 Fig) and could correspond to a distinct species. The collection of fresh material is essential before making taxonomic decisions concerning these species.

### The *G*. *brevispinis* complex

The integrative taxonomy methodology reviewed in Padial et al. [25] was sufficient to congruently discriminate eight species of *Guyanancistrus*, including five new species, and a new genus of the Loricariidae (see Table 2). However, *G*. *brevispinis* could not be significantly distinguished from all other species using the different approaches except phylogeny alone, and its different subspecies could not be clearly delineated regardless of the method employed (morphometry, DNA barcodes, phylogeny, or distribution). In this context, use of the multi-table approach integrating all available information was particularly suitable. This method allowed the evaluation of the amount of common information present in the different datasets and its significance through RV tests, and demonstrated that half of the variation recorded in the different tables was significantly linked. Indeed, the unifying structure provided by the MCOA simultaneously revealed significant covariations between morphometric characteristics, phylogenetic structure, and distributional patterns in all available populations of *G*. *brevispinis*, and clearly highlighted three groups of infraspecific rank. Surprisingly, the presence of two lineages of *G*. *brevispinis* within the Maroni Basin was revealed. One lineage included all populations of the eastern Maroni grouped with other populations of *G*. *b*. *bifax*, and the second was located in the Upper Tapanohony River, a western tributary of the Maroni River, grouped with the populations of *G*. *b*. *brevispinis* close to populations from Upper Suriname and Corantijn rivers. This unexpected result highlights the central role played by the Maroni Basin in the distributional pattern of *G*. *brevispinis* members, with an east-west partition of this drainage. This central role was also confirmed by the biogeographic reconstruction, which resolved no fewer than five successive dispersal events originating from the Maroni Basin toward other drainages of the Eastern Guianas. Two of them concerned ancestral populations of *G*. *brevispinis* which dispersed to the west toward the Upper Suriname and Corantijn rivers respectively (leading to establishment of the future *G*. *b*. *brevispinis*), a third eastward toward the Upper Oyapock (future *G*.*b*. *orientalis*), and a fourth and fifth toward the Sinnamary and Mana respectively (future *G*.*b*. *bifax*), but with the persistence of two distinct lineages within the Maroni Basin.

These results only partially corroborate the findings of Cardoso and Montoya-Burgos [60] who recovered five lineages (instead of three) among *G*. *brevispinis* including distinct lineages from: (1) Oyapock-Comté-Approuague basins, (2) Maroni-Mana-Sinnamary basins, (3) Suriname River, (4) Corantijn River, and (5) Nickerie River. However, genetic distances between the Nickerie and Suriname rivers’ representatives in their phylogenetic tree were not markedly greater than those within their Maroni-Mana-Sinnamary lineage (our *G*. *b*. *bifax*), such as the representatives of the Sinnamary and Mana basins. These authors also highlighted the central role played by the Maroni Basin as the gateway of ancestors of *G*. *brevispinis* from the Amazon Basin. Using phylogenetic topological tests, they hypothesized a single entrance from headwaters of the Maroni River followed by a first westward dispersal, assuming a stepping-stone pattern of dispersal. Then dispersal strategies were evaluated based on haplotypic diversity and genetic-geographic structure comparisons between populations here described as *G*. *b*. *bifax*, revealing favoured dispersal routes through coalescing river mouths during low sea level periods. If the entrance of ancestral forms of *Guyanancistrus* originating from the Amazon Basin in the Maroni River is confirmed by the present study, the reconstructed dispersal pattern of *G*. *brevispinis* members is much more complex than a simple stepping-stone process, probably related to river capture events (direct dispersal from the Maroni to the Corantijn and Oyapock rivers). In addition, Cardoso and Montoya-Burgos [60] reported a single Amazonas lineage which has been shown in the present study to contain three distinct species: *G*. *teretirostris* from Paru de Oeste River (Pb.BR652, Pb.BR653, and Pb.BR654 in Cardoso and Montoya-Burgos, 2009 [60]), *G*. *tenuis* from Jari River (Pb.MIT03, and Pb.MIT04 in Cardoso and Montoya-Burgos, 2009 [60]), and *G*. *megastictus* from Maroni River (Pb.MIT02 in Cardoso and Montoya-Burgos, 2009 [60]), leading to two dispersal events from the Amazonian tributaries toward the Maroni Basin for their study. The present study also revealed a third dispersal between Paru de Oeste and Saramacca rivers. All these dispersals resulted in speciation within the Eastern Guianas, with the particularity of the Maroni River hosting three newly formed species; two hyperendemics restricted to montaneous areas (*G*. *nassauensis* and *G*. *megastictus*), and one widely distributed (*G*. *brevispinis*) and comprising two subspecies (*G*. *b*. *brevispinis* and *G*. *b*. *bifax*). The Maroni Basin was thus a center of speciation for *Guyanancistrus* members resulting in increased local endemicity, as well as a source of dispersal to other drainages of the Eastern Guianas.

Cardoso and Montoya-Burgos [60] tentavely provided diagnostic characters to distinguish their different lineages, but most of them relyed on global estimates of shape and color patterns. Conversely, the MCOA used here, by unifying different variables contained in different data sets within the same analysis, allowed the extraction of diagnostic characteristics for each group. Moreover, the ability to include phylogeny with the other data sets allowed the interpretation of covariations between morphometric, phylogenetic, and distributional variables in an evolutionary perspective. Evolution of shape of *G*. *brevispinis* members was thereby linked to genetic and geographic divergences. *Guyanancistrus brevispinis bifax* evolved a mean shape intermediary between *G*. *b*. *brevispinis* to the west and *G*. *b*. *orientalis* to the east, characterized by more numerous plates on the caudal peduncle, which was also less deep in this subspecies. This result contrasted with that of Cardoso and Montoya-Burgos [60], who characterized the same group as having the highest body shape. *Guyanancistrus b*. *brevispinis* evolved a broader head and anterior body in the west whereas *G*. *b*. *orientalis* evolved a slender appearance with a longer caudal peduncle and more numerous plates along the body in the east, a result in general agreement with Cardoso and Montoya-Burgos [60]. It is interesting to note that *G*. *b*. *bifax* possesses two morphs (see Fig 10), both present in the whole area of distribution of the subspecies. Even though not statistically supported, morphotypes with broad mouth (identified by the letters BM in Fig 1) were all placed closer to *G*. *nassauensis* in the morphometric analysis, and appeared clearly distinct from other morphotypes with a normal mouth. Given that *G*. *nassauensis* was introgressed by *G*. *brevispinis* (see Fig 2), implying hybridization between these two co-occuring (at least in the Nassau Mountains) sister species, this morphological characteristic may result from retention of genes from *G*. *nassauensis* in the genome of *G*. *b*. *bifax*.

Color patterns also appeared highly variable in *G*. *brevispinis*. Polychromatism is not rare in fish and can be related to sexual selection driving the appearance of strong sexual dimorphism ([119–122], reviewed in [123]). Numerous genera of the Loricariidae exibit strong sexual dimorphism through the development of hypertrophied odontodes (e.g. in *Peckoltia*, *Panaque*, *Neblinichthys*, *Sturisoma*, *Farlowella*, *Spatuloricaria*, *Rineloricaria*), development of fleshy tentacles on snout (e.g. in *Ancistrus*), lip enlargement (e.g. in *Loricariichthys*, *Hemiodontichthys*), or teeth characteristics (e.g. in *Loricaria*), but dichromatism has not previously been reported, even though several genera display colorful patterns (e.g. *Pseudacanthicus*, *Leporacanthicus*, *Hypancistrus*, *Scobinancistrus*, *Peckoltia*, *Panaqolus*). Color variations in Loricariidae appear sex-independent, and rather related to natural selection (e.g. for camouflage over the substrate) or to random drift. However, such variations in *G*. *brevispinis*, with the appearance of very diverse patterns ranging from spots to marbling and reticulations imply rather relaxed selective constraints acting on phenotypes since different patterns can be observed within the same basin. Alternatively similar patterns can be observed between distant basins implying multiple convergent evolution and/or retention of ancestral patterns among populations. Cardoso and Montoya-Burgos [60] tentatively classified their different lineages using pattern characteristics, but if this criterion applied for a given population, it often failed to characterize other populations of the same basin or equally applied to other populations of a distinct lineage (see Fig 8). For example, Cardoso and Montoya-Burgos [60] distinguished their lineage from the Corantijn River (Sipaliwini River; Fig 8A and 8B), from all other populations by the head having small light vermiform marks and the body faint parallel light bands becoming highly visible on the caudal peduncle. However, Fig 8C, corresponding to another population from the Corantijn Basin (Kabalebo River), shows that this population was particularly dark, without such obvious markings. The same situation occurred with the Comté-Approuague-Oyapock lineage (*G*. *b*. *orientalis*), which was supposed to be distinguished from all other lineages by the head having small light dots and the body faint, parallel, light bands becoming highly visible on the caudal peduncle; the specimen from the Comté-Orapu Basin (Fig 8I) and the one from the Oyapock River (Fig 8J), are clearly distinct from each other. Moreover, the characteristics of the Oyapock population better reflected the definition provided for the Maroni-Mana-Sinnamary lineage (*G*. *b*. *bifax*), theoretically distinguished by large light dots, irregular in shape [60]. Given the high variability of the species, color patterns do not appear to be relevant for identification purposes.

### Notes on the ecology of *Guyanancistrus* species

*Guyanancistrus brevispinis* occurs in the lowland rivers and large tributaries in the interior of Suriname and French Guiana (i.e. upstream of the most downstream rapids), mainly in strong currents in or immediately downstream of rapids. During the day adult *G*. *brevispinis* were observed foraging on the algal biofilm on boulders and bedrock in the Middle Suriname River together with *Cteniloricaria platystoma* and *Harttia surinamensis*. Adult *G*. *brevispinis* are well camouflaged when feeding on these substrates. Juveniles of *G*. *brevispinis* were collected in a mountain stream (400 m above mean sea level) in Lely Mountains [56] with cool (23.3°C), clear (Secchi disc visibility 150 cm) water with low conductivity (24 μS cm^-1^) and neutral pH of 7.5. Postlarvae of *G*. *brevispinis* (15 mm TL) were collected in a headwater tributary of the Upper Palumeu River in a deep (> 1 m) pool under a 60-m high waterfall (Fig 8.1. in Mol & Wan Tong You [124]); the water was cool (23.5°C), clear (turbidity 5 NTU), slightly acidic (pH 5.9) with low conductivity (20 μS cm^-1^), low alkalinity (4.75 mg CaCO_3_ L^-1^) and some tannins (2.6 mg L^-1^) [125].

*G*. *brevispinis* has the largest distribution within Eastern Guianas, with an area of distribution ranging from the Corantijn River in western Suriname to the Oyapock River in eastern French Guiana (Fig 4). Alternatively, most of the other Guianese species (the Amazonian species are insufficiently known) appear highly restricted to mountains, having a similar distributional pattern to *Harttiella* [19, 55, 56], a group of hyperendemic dwarf loricariids restricted to mountainous forest creeks. At least two species of *Guyanancistrus* (*G*. *nassauensis* and *G*. *brownsbergensis*) have developed adaptations to this kind of biotope (small streams, cool water temperature, low productivity…) including dwarfism.

*Guyanancistrus nassauensis* and *G*. *brownsbergensis* are each known from a single mountain stream, in the Nassau Mountains (Paramaka Creek) and Brownsberg Mountains (Kumbu Creek), respectively. With this very restricted distribution (< 20x20 km^2^) both species can be considered hyperendemics and currently the two species are threatened with extinction by proposed and ongoing mining activities.

In Paramaka Creek, *Guyanancistrus nassauensis* occurs syntopically with juvenile *Guyanancistrus brevispinis* and with *Harttiella crassicauda*, a second endemic species from the Nassau Mountains. However, *G*. *nassauensis* occurs both on the plateau in perennial flowing headwaters and in the upper mainstem of Paramaka Creek (lower slopes of the plateau; altitude range 120–530 m amsl), whereas *H*. *crassicauda* only occurs on the plateau proper (230–530 m amsl; [126]). In the IJs Creek tributary of Paramaka Creek on the Nassau plateau (467 m amsl) both *G*. *nassauensis* and *H*. *crassicauda* occur in cool (22.6°C), shallow (40 cm water depth), clear (Secchi transparency > 40 cm) water with low conductivity (28 μS cm^-1^), neutral pH of 7, low inorganic N (0.067–0.120 mg L^-1^), relatively high organic N (0.307–0.592 mg L^-1^), low total P (0.002–0.010 mg L^-1^) and high organic C (2.916–4.972 mg L^-1^) [56]. The bottom substrate is gravel with boulders and bedrock (with the red filamentous algae *Batrachospermum* sp. attached to it) and near the edge of the plateau in slightly deeper water (approximately 50 cm) stands of the emergent macrophyte *Thurnia sphaerocephala* occur [56]. In the upper mainstem of Paramaka Creek, as well as in some upstream branches on the plateau, *G*. *nassauensis* occurs syntopically with *G*. *brevispinis* [126].

*Guyanancistrus brownsbergensis* was collected only in the upper reaches of Kumbu Creek on the Brownsberg bauxite plateau at an altitude of 200–430 m amsl. The habitat of *G*. *brownsbergensis* seems very similar to that of *G*. *nassauensis* in the Upper Paramaka Creek on the Nassau bauxite plateau. The upper Kumbu Creek is a small (2.5–3.7 m wide, 26–52 cm deep) mountain stream with moderate to strong flow (0.3–0.56 m s^-1^) and cool (23.1–23.2°C), mostly clear water with high dissolved oxygen content (7.1–7.7. mg L^-1^), neutral pH of 7–7.5 and low electrolyte content (30.8–31.6 μS cm^-1^). The bottom substrate is mainly gravel, boulders and bedrock. We observed no aquatic vegetation in the stream, but overhanging terrestrial vegetation, submersed root masses, woody debris, leaf litter and rock crevices offered ample hiding places for *G*. *brownsbergensis*. During the day, adult *G*. *brownsbergensis* were observed on several occasions throughout the year resting in moderate current in front of a rock crevice in a relatively deep (50 cm) pool upstream of the 50-m high Kumbu Falls.

We have no information on the ecology of *G*. *teretirostris*, *C*. *similis*, *G*. *tenuis* and *G*. *megastictus*, although the latter two species apparently also occur in small mountain streams, perhaps comparable to the habitat of *G*. *nassauensis* and *G*. *brownsbergensis*.

Other species are larger, particularly when they inhabit the main stream of rivers as does *G*. *niger*, the largest species of *Guyanancistrus*, which lives in the rapids of the Oyapock River along with *Pseudancistrus barbatus*. *Guyanancistrus niger* is much less abundant than other species, and we collected only adult specimens, suggesting that adults and juveniles may not be syntopic. *Guyanancistrus brevispinis* and *G*. *longispinis* were collected together in the Oyapock drainage, but while *G*. *brevispinis* seems to prefer small forest streams, *G*. *longispinis* was more often found in the main channel, on the rocky bottom of riffles, where it can be relatively abundant ([59]; personal observations).

## Supporting information

S1 Text Supplementary material examined for this study. Species are listed in alphabetical order, followed by country, river basin, catalog number, number of specimens examined in the lot, locality, collector, and date of sampling. Specimens included in morphometric analyses are indicated by an asterisk followed by number when needed.
(DOCX)

S1 Table Raw morphometric and meristic data for the 269 specimens analysed in the morphometric study. Measurements are in mm. Variables labelled as in Tables 4 and 5, and species, populations, and morphs labelled as in Fig 1.
(XLSX)

S1 Fig Between Group Analysis (BGA) of the different species of *Guyanancistrus*, *Cryptancistrus*, and other putatively related species. *Hemiancistrus medians* was added to the dataset to evaluate the similarity between *Chaetostomus macrops* Lütken 1874 and *Guyanancistrus* or *Hemiancistrus*. **a**: projection of 284 specimens distributed in 12 species and 3 subspecies onto the first factorial plane of the BGA (axis 1 horizontal, axis 2 vertical); **b**: projection of the morphometric (n = 24) and meristic (n = 14) variables onto the first factorial plane of the BGA; variables labelled as in Tables 4 and 5. **c**: eigenvalues of the BGA. *C*. *macrops* appeared closer to members of the *G*. *longispinis* group, and particularly the species of *Guyanancistrus* from Potaro River (GspPotaro) identified as `*Pseudancistrus’ megacephalus* by Eigenmann in 1912. `*P*.*’ megacephalus* (positive score on axis 2) also appeared distinct from *C*. *macrops* (negative score on axis 2), and both of them distinct from *H*. *medians*, type species of *Hemiancistrus*.
(TIF)
