# Supplementary material for: An integrative framework to reevaluate the Neotropical catfish genus Guyanancistrus (Siluriformes: Loricariidae) with particular emphasis on the Guyanancistrus brevispinis complex
Source: PLoS One. 2018 Jan 3;13(1):e0189789. doi: 10.1371/journal.pone.0189789 (PMC5752014; doi:10.1371/journal.pone.0189789)
Supplement: S1 Text — Species are listed in alphabetical order, followed by country, river basin, catalog number, number of specimens examined in the lot, locality, collector, and date of sampling. Specimens included in morphometric analyses are indicated by an asterisk followed by number when needed. (DOCX) [file pone.0189789.s001.docx]

***G. brevispinis brevispinis***: from West to East: **Suriname: Corantijne River Basin**: – MHNG 2704.008, 6* of 16, Manicouni River among confluence with Sipaliwini River; Covain et al., 20 Oct. 2007. – MHNG 2708.41, 1, Pidi Touman Creek, right bank tributary of Manicouni River; same collectors and date.– MHNG 2704.010, 6* of 19, Rapids of the Sipaliwini River; Covain et al., 22 Oct. 2007. – MHNG 2704.013, 18; MHNG 2723.006, 2, Sipaliwini River, at Sipaliwini; Covain et al., 19 Oct. 2007. – MHNG 2704.011, 1, Sipaliwini River downstream of Sipaliwini; Covain et al., 21 Oct. 2007. – MHNG 2704.012, 13, left bank tributary of Sipaliwini River downstream of Sipaliwini; Covain et al., 23 Oct. 2007. – MHNG 2704.009, 4, Tipiteu Eku Creek, tributary of Sipaliwini River, 1 hour walk East of Sipaliwini; Covain et al., 24 Oct. 2007.– MHNG 2736.039, 12*of 84, Moi Moi falls, Kabalebo River tributary; Covain & Fisch-Muller, 29 Oct. 2012. **Nickerie River Basin:** – MHNG 2722.041, 1*; MHNG 2722.044, 2*; MHNG 2722.051, 1*; MHNG 2722.055, 1*, tributaries of Nickerie River in Bakhuis Mountains, Mol, 31 May-8 June 2006. – MHNG 2621.073, 2 postlarves, rapids of upper Nickerie River upstream of Blanche Marie Falls; Weber, Commergnat & Mol, 31 Oct. 2001. – MHNG 2767.074, 2 juveniles, Bakhuis Mountains; Mol, 1 June 2006. – MHNG 2767.075, 2 juveniles, Bakhuis Mountains; Mol, 2 June 2006. – MHNG 2753.091, 13* on 22, Moses Creek, tributary of Nickerie River; Covain et al., 17-19 Oct. 2014. **Saramacca River Basin**: - MHNG 2758.058, 2* on 3 juveniles, Latambo Creek, tributary of Upper Saramacca River upstream Poesoegroenoe; Covain et al., 14 Oct. 2014. **Suriname River Basin:** – MHNG 2673.034, 16* of 48, Cajana Creek, tributary of Gran Rio River, MZUSP 117152, 3, same locality; Covain et al., 2 Nov. 2005. – MHNG 2673.048, 20, downstream of latter locality; Covain et al., 31 Oct. 2005. **Maroni River Basin:** – MHNG 2717.035, 9* of 11; MHNG 2726.060, 1*; MHNG 2717.049, 6, Wayu Camp, Palomeu River, tributary of Tapanahony River; Covain et al., 28 Nov. 2008.

***G. brevispinis bifax*:** French Guiana, from West to East: **Maroni River Basin**: – MHNG 2593.088, 1, tributary of Grand Inini River upstream of Saut “S”; Le Bail et al., 30 Sep. 1997. – MNHN 2015-230 4, reaches of Grand Inini River; Le Bail et al., 28. Sep. 1997. – MHNG 2752.025, 3; MNHN 2015-231, 3; Saut Nicole, Grand Inini River; Le Bail et al., 26-28 Sep. 1997. – MHNG 2752.026, 5; MNHN 2015-232, 5 ; Creek tributary of Grand Inini ; Le Bail et al., 27 Sep. 1997. – MHNG 2589.056, 2, Inini River at confluence of Grand Inini and Petit Inini rivers; Bingelli, Sep. 1993. – MHNG 2727.032, 1, Saut Sonnelle, Inini River; Vigouroux, 10 Sep. 2009. - MHNG 2752.027, 1, Saut Gostou, Maroni River; Planquette, 12.10.1979. – MHNG 2725.099 (ex MNHN 2000-5728), 1 (measured); Litani River upstream Antecume Pata; Fisch-Muller et al., 17 Oct. 2000. – MHNG 2752.004, 1, Saut Tula Lapata, Marouini River; Fisch-Muller et al., 7 Oct. 2000. – MHNG 2752.005, 2; MHNG 2752.006, 1; MHNG 2752.007, 6; Marouini River at Antecume Pata; S. Fisch-Muller et al., 19-20 Oct. 2000. – MHNG 2757.027, 9; MHNG 2758.016, 3; Saut Wayo, Marouini River; Covain et al., 23-27 Sep. 2014. – MHNG 2752.028, 1; MNHN 2015-233, 1; Saut Singatetei, Litani River; Planquette & Boeseman, 15.10.1979. - MNHN 2000-5734, 6; rapids of Litani River west of Antecume Pata; Jégu et al., 24.10.2000. – MNHN 2000-5742, 1, reach of the Litani River downstream of Antecume Pata; Jégu et al., Oct. 2000. – MNHN 2000-5770, 1, Saut Pierkuru, Tampoc River; Jégu et al., 13.10.2000. – MHNG 2683.029, 6 (measured); MHNG 2683.043 (GF06-480, 481), 26 (10 measured); MNHN 2015-222, 5; Crique Voltaire at Voltaire camp, tributary of Lower Maroni; Fisch-Muller et al., 12 Nov. 2006. **Mana River Basin:**– MHNG 2683.050, 5; MHNG 2683.056, 8; Crique Portal, tributary of Mana River; Fisch-Muller et al., 13-14 Nov. 2006.– MHNG 2683.079, 25, tributary of Crique Petit Laussat; Covain & Fisch-Muller, 14 Oct. 2012. – MHNG 2683.069, 2, Crique Grand Laussat, tributary of Mana River; Fisch-Muller et al., 14 Nov. 2006. – MHNG 2699.060, 22 (10 measured); MHNG 2699.053, 1; MHNG 2699.056, 3; MHNG 2700.045, 3, Crique Aya near Aya camp, tributary of Crique Baboon; Montoya-Burgos & Melki, 28 Nov. 2007.– MHNG 2723.017, 3, Mana River, Saut Ananas; Le Bail et al., 20 Sep. 1995. – MHNG 2752.029, 2; MNHN 2015-235, 3; Mana River, Saut Capiai; Le Bail et al., 25 Sep. 1995. –MNHN 2015-236, 2, Citron, Crique Lézard, tributary of Mana River; Le Bail & Planquette, 7 Oct. 1995. - MHNG 2727.039, 7, tributary of Mana River; Vigouroux, 28 Oct. 2009. **Sinnamary River Basin:** – MHNG 2723.015, 8 (6 measured), MNHN 2015-226, 9 ; Crique Coeur Maroni, near Petit Saut; Le Bail et al., 2 Feb. 1983. – MNHN 2015-237, 2, Petit Saut, Sinnamary River; Le Bail et al., 11 March 1983. – MHNG 2723.007, 3 (measured), Upper Sinnamary River at Saut Vata; Lauzanne & de Morais, 23 Oct. 1992. – MHNG 2723.008, 7 (measured); MHNG 2723.009, 7 (measured), Crique Maïpouri; Lauzanne & de Morais, 24 Oct. 1993. – MHNG 2751.073, 1 V, Upper Sinnamary River at Saut Deux Roros; IRD, 18 Nov. 1999. – MHNG 2767.079, 7, Upper Sinnamary River at Saut Parasol; IRD. – MHNG 2585.018, 1 V, Crique Grégoire; Ponton & Copp, 28 August 1994. – MHNG 2722.068, 17 (3 measured), Crique Grégoire; Weber et al., 7 Nov. 2003. – MHNG 2737.040, 1, Crique Grégoire just downstream dam; Quartarollo et al., 25 Oct. 2012. – MHNG 2722.071, 1, Sinnamary River, Saut Takari Tanté; Vigouroux, 15 Oct. 2003.

***G. brevispinis orientalis*:** French Guiana, from West to East**: Mahury River Basin:** – MHNG 2682.074, 7 (4 measured), Crique Nuage at Facouzi, tributary of Orapu River; Fisch-Muller et al., 10 Nov. 2006. – MHNG 2682.047, 14 (6 measured), Crique Grillon near carbet ONF, tributary of Orapu River; Fisch-Muller et al., 7-8 Nov. 2006. – MHNG 2722.065, 11; MHNG 2722.066, 69 (2 measured), MNHN 2015-223, 40, same locality, MZUSP 117151, 3, same locality; Weber et al., 8 Nov. 2003. – MHNG 2680.020, 1, Crique Maripa, tributary of Orapu River; Rauschert, 1952– MNHN 2015-238, 5, Crique Boulanger, tributary of Orapu River; Planquette, 4.10.1979,.- MHNG 2752.031, 6, Crique Fourgassié, tributary of Orapu River; Fisch-Muller et al., 12.10.1999. – MHNG 2712.090, 1, Crique Georgeons, tributary of Comté River; Vigouroux, 2008. – MHNG 2752.032, 3, pisciculture Gabriel, Comté River; Fisch-Muller et al., 8.10.1999. **Kaw River Basin:** – MHNG 2723.011, 7 (6 measured); MNHN 2015-229, 4; Crique Diamant, tributary of Kaw River, around 300 meter downstream of Patawa camp, Fisch-Muller & Montoya-Burgos, 7 Oct. 1999. – MHNG 2723.010, 4; MNHN 2015-224 , 5; Crique Diamant; Keith et al., 19 Oct. 2000.  **Approuague River Basin:** – MNHN 1994-0784, 2 (measured), rapids of Saut Mapaou, Approuague River; Boujard et al., March 1990. – MHNG 2621.099, 2 (measured), same locality; Weber et al., 5 Nov. 2001. – MNHN 2015-239, 1, Approuague River; Planquette, Sep. 1995. – MHNG 2522.072, 1*, Crique Arataï; Boujard et al., 14 Nov. 1988. - MHNG 2480.046, 1 (measured), Saut Japigny, Crique Arataï, tributary of Upper Approuague River; Boujard et al., 16 Nov. 1988 – MHNG 2662.089, 4 (3 measured); MHNG 2662.094, 1*, same locality; Fisch-Muller et al., 20 Nov. 2003. – MHNG 2662.092, 3 (2 measured), Saut Pararé, Crique Arataï at; Fisch-Muller et al., 21 Nov. 2003. – MHNG 2662.098, 1 (measured), Crique Nourague, tributary of Crique Arataï; Fisch-Muller et al., 21 Nov. 2003. – MHNG 2701.090, 2 (1 measured), Crique Arataï; Vigouroux, 4 Nov. 2007. – MHNG 2701.088, 2, Crique Balenfois, tributary of Approuague River; Vigouroux, 3 Oct. 2007. – MHNG 2757.035, 10; MHNG 2559.037, 2; Crique Kapini, tributary of Approuague River; Covain et al., 27 Oct. 2014. **Oyapock River Basin:** – MHNG 2723.014, 5 (2 measured); MHNG 2744.039, 6, postlarvae; MNHN 2015-220, 3; Crique Minette, tributary of Lower Oyapock River; Fisch-Muller et al., 21 Oct. 1999. Brazil, Amapá: ZSM 27836, 4, Cachoeira Grande Rocha, about 7 km. upstream from Oiapoque; R. Stawikowski & P. Ludwig, 18 Sep. 1989. – ZSM 27838, 8, Rio Pantanari, Cachoeira about 4km upstream from confluence with Rio Oiapoque; R. Stawikowski & P. Ludwig, 18 Sep. 1989.

***Guyanancistrus longispinis***: All from French Guiana: Oyapock River Basin: – MHNG 2725.100, 7; MNHN 2015-228, 3; Crique Gabaret, tributary of Lower Oyapock River; Fisch-Muller et al., 21 Oct. 1999. - MHNG 2680.100, 3, Oyapock River at Alicoto; Fisch-Muller et al., 3 Nov. 2006. – MHNG 2681.049, 3; MHNG 2681.074, 1, Oyapock River at Saut Kamalawa; Fisch-Muller et al., 4-5 Nov. 2006. – MHNG 2681.059, 1, Fifine Creek, tributary of Oyapock River; Fisch-Muller et al., 5 Nov. 2006. – MHNG 2681.067, 3, Oyapock River at Saut Wakarayou; Fisch-Muller et al., 5 Nov. 2006. – MHNG 2752.035, 1, Oyapock River; Planquette, 1986.

***Guyanancistrus niger***: French Guiana: Oyapock River Basin: – MHNG 2752.035, 1, Oyapock River; Planquette, 1986. – MNHN 2015-240, 2, Oyapock River; Planquette, Dec. 1986. – MNHN 2015-241, 1, Oyapock River; Hohler, Dec. 1986. – MHNG 2722.089, 1, Oyapock River upstream Saut Maripa; Fisch-Muller et al., 20 Oct. 1999. – MHNG 2682.037, 1, confluence of forest creek tributary of Oyapock River in front of Roche mon Père; Fisch-Muller et al., 6 Nov. 2006 . – MHNG 2753.072, 1, Oyapock River, Saut Maripa; Covain et al, 28 Oct. 2014. – MHNG 2682.037, 1, confluence of forest creek tributary of Oyapock River in front of Roche mon Père; Fisch-Muller et al., 6 Nov. 2006. – MHNG 2727.049, 1, Camopi River, tributary of Oyapock River, at Saut Alexis; Vigouroux, 15 Oct. 2009. Brazil, Amapá: ZSM 27833, 4, Cachoeira Grande Rocha, about 7 km. upstream from Oiapoque; R. Stawikowski & P. Ludwig, 18 Sep. 1989.

***Guyanancistrus megacephalus***: Holotype: BMNH 1978.9.12: 3, 122.8 mm SL*; (probably from) Surinam; Lidth van de Jeude (on label: Suriname, British Guiana; Damon).

***Guyanancistrus* sp**. (*Guyanancistrus megacephalus* sensu Eigenmann, 1912): MCZ 30196, 1*; Guyana, Lower Potaro River at Amatuk Fall; Eigenmann, 30 Oct. 1908. – ZMB 17953, 1*; Guyana, Potaro River; Eigenmann.

***Hopliancistrus tricornis*.** Brazil: – MHNG 2585.065, 1, Tapajós River, Cachoeira Credo; Stawikowski et al., 28 Sep 1992. – MHNG 2547.003, 1, Tapajós River, Alter do Chao; Harnoss, Sep. 1992. – MHNG 2585.066, 2, Tapajós River, 2 km upstream Pimental ; Harnoss, 15 Sep 1992. - MHNG 2547.002, 4, Tapajós River drainage, Santa Cruz River,  Balneário Lisote; Werner et al., 25 July 1993. – MHNG 2497.061, 1, Xingú River, Altamira; aquarium trade, 1989. – MHNG 2588.051, 6; aquarium trade (Manaus), 1992.
