# Supplementary figures and images for: An integrative framework to reevaluate the Neotropical catfish genus Guyanancistrus (Siluriformes: Loricariidae) with particular emphasis on the Guyanancistrus brevispinis complex
Source: PLoS One. 2018 Jan 3;13(1):e0189789. doi: 10.1371/journal.pone.0189789 (PMC5752014; doi:10.1371/journal.pone.0189789)

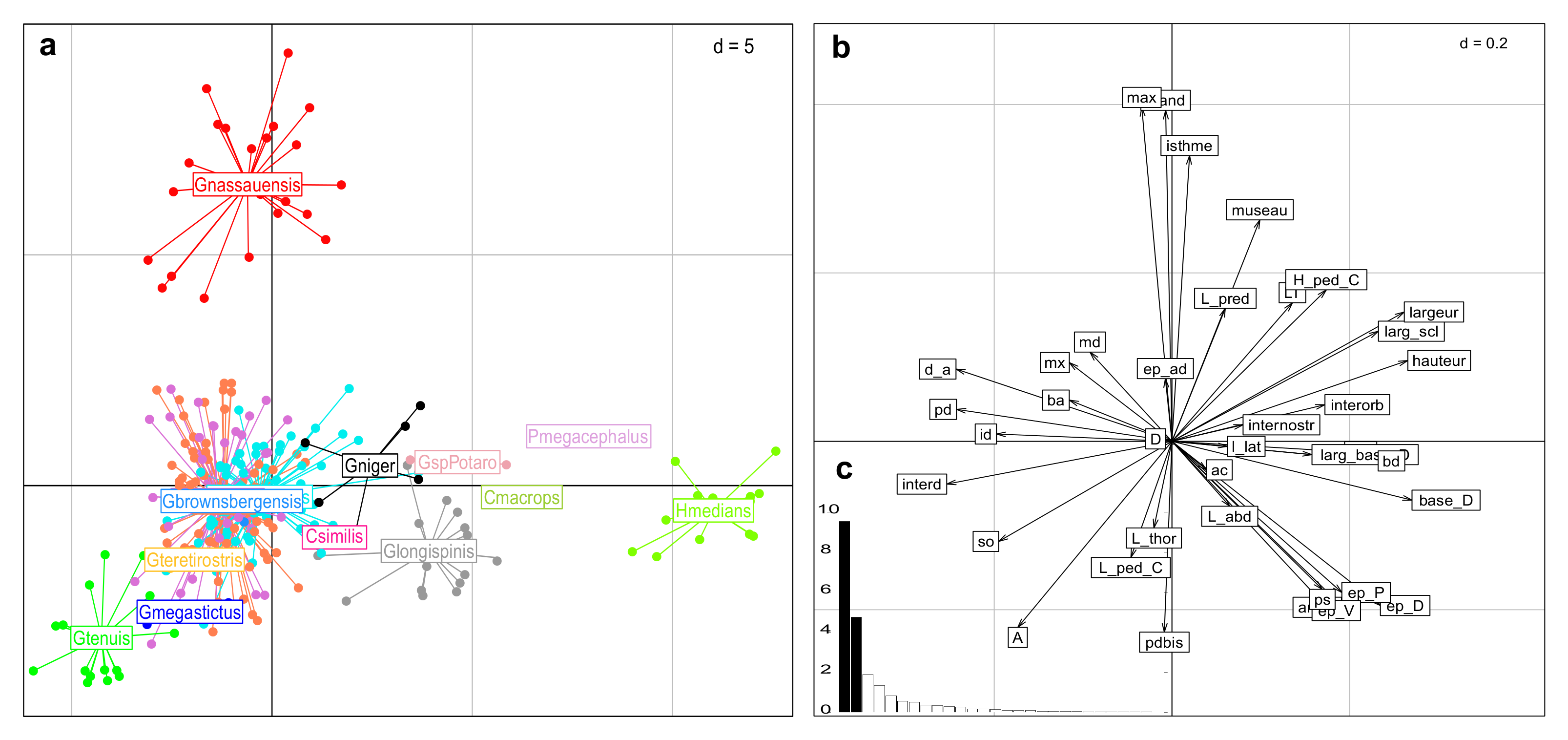

Supplement: S1 Fig — Hemiancistrus medians was added to the dataset to evaluate the similarity between Chaetostomus macrops Lütken 1874 and Guyanancistrus or Hemiancistrus. a: projection of 284 specimens distributed in 12 species and 3 subspecies onto the first factorial plane of the BGA (axis 1 horizontal, axis 2 vertical); b: projection of the morphometric (n = 24) and meristic (n = 14) variables onto the first factorial plane of the BGA; variables labelled as in Tables 4 and 5. c: eigenvalues of the BGA. C. macrops appeared closer to members of the G. longispinis group, and particularly the species of Guyanancistrus from Potaro River (GspPotaro) identified as `Pseudancistrus’ megacephalus by Eigenmann in 1912. `P.’ megacephalus (positive score on axis 2) also appeared distinct from C. macrops (negative score on axis 2), and both of them distinct from H. medians, type species of Hemiancistrus. (TIF) [file pone.0189789.s003.tif]
